# The RNA-binding protein DAZL functions as repressor and activator of mRNA translation during oocyte maturation

**DOI:** 10.1038/s41467-020-15209-9

**Published:** 2020-03-13

**Authors:** Cai-Rong Yang, Gabriel Rajkovic, Enrico Maria Daldello, Xuan G. Luong, Jing Chen, Marco Conti

**Affiliations:** 1 Center for Reproductive Sciences, University of California, San Francisco, CA 94143 USA; 2 USA Eli and Edythe Broad Center of Regeneration Medicine and Stem Cell Research, University of California, San Francisco, CA 94143 USA; 3 Department of Obstetrics and Gynecology and Reproductive Sciences, University of California, San Francisco, CA 94143 USA

**Keywords:** Developmental biology, Embryology

## Abstract

Deleted in azoospermia-like (DAZL) is an RNA-binding protein critical for gamete development. In full-grown oocytes, the DAZL protein increases 4-fold during reentry into the meiotic cell cycle. Here, we have investigated the functional significance of this accumulation at a genome-wide level. Depletion of DAZL causes a block in maturation and widespread disruption in the pattern of ribosome loading on maternal transcripts. In addition to decreased translation, DAZL depletion also causes translational activation of a distinct subset of mRNAs both in quiescent and maturing oocytes, a function recapitulated with YFP-3′UTR reporters. DAZL binds to mRNAs whose translation is both repressed and activated during maturation. Injection of recombinant DAZL protein in DAZL-depleted oocytes rescues the translation and maturation to MII. Mutagenesis of putative DAZL-binding sites in these mRNAs mimics the effect of DAZL depletion. These findings demonstrate that DAZL regulates translation of maternal mRNAs, functioning both as the translational repressor and activator during oocyte maturation.

## Abstract (web summary)

The RNA binding protein DAZL plays a critical role during germ cell development. Here the authors provide evidence that DAZL functions both as activator and repressor of translation during oocyte maturation in mouse.

## Introduction

In most species, male and female gamete production is a developmental process that spans across embryonic, fetal, and postnatal life—a process essential for the transfer of genetic information to the progeny^1^. Germline lineage specification, expansion of the gamete precursors (PGCs), meiosis, and terminal differentiation into functional gametes are all elaborate processes that require extensive regulation of gene expression^1,2^. Together with unique transcriptional and epigenetic mechanisms, regulation of translation plays a critical role in the differentiation of germ cells^3–5^.

In a mature mRNA molecule, several domains contribute to the regulation of translational efficiency and stability^6^—the 5′ cap region of the mRNA, 5′ and 3′UTRs, and poly(A) tail^6–8^. Translation of an mRNA is modulated through interaction with proteins, often aggregating in large macromolecular complexes, as well as with non-coding RNAs^9,10^. Indeed, the assembly of ribonucleoprotein (RNPs) modulates every aspect of mRNA translation and stability. A subgroup of RNA-binding protein (RBPs) interacts with the 3′UTR of an mRNA during the formation of RNPs that are critical for the control of mRNA translational efficiency, stability, and subcellular localization^11^, thus underscoring their critical role in the control of protein synthesis. Among the several RBPs uniquely expressed in the germ line, DAZ, DAZL and BOULE are germ-cell specific RBPs essential for gametogenesis from worms to humans^12,13^.

DAZL gene inactivation prevents differentiation of PGCs^12,14,15^. Mechanistically, it has been proposed that DAZL functions as a translational activator by recruiting poly(A) binding proteins^13^. In turn, these complexes promote and stabilize interaction with mRNA cap, a loop conformation thought to promote stability and translational efficiency^16^. However, additional studies in PGCs suggest a repressive function for this RBP in the control of fetal gonad development and embryonic stem cell in the mouse^17,18^. Moreover, depletion of DAZL during postnatal spermatogenesis has been associated with mRNA destabilization^19^ or translational activation^20^.

We have previously reported that acute depletion of DAZL from fully grown mouse oocytes using morpholino oligonucleotides causes disruption of the progression through meiosis^21,22^. Here we have used this in vitro model to define the pattern of translation dependent on the function of DAZL. DAZL depletion causes increases and decreases in translational efficiency of a wide range of transcripts expressed in the oocyte. These effects are reversible and recapitulated by regulation of reporter translation of candidate DAZL targets. Thus, DAZL serves function in the program of maternal mRNA translation which are dependent on the 3′UTR context and interacting RBPs, a preeminent one being CPEB1.

## Results

### Inhibition of Dazl mRNA translation decreases Dazl protein levels

**Fig. 1:**
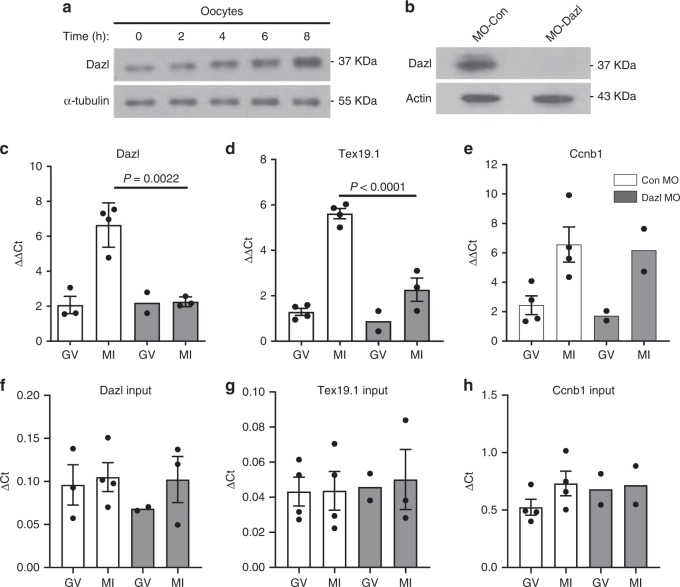
Interference with Dazl mRNA translation depletes oocytes of the DAZL protein and inhibits translation of a specific downstream target. **a** DAZL protein accumulation during the transition from the GV-to-MI stages of oocyte maturation. GV stage oocytes from wild type mice were cultured in vitro up to 8 h of maturation and used for Western blot analysis. 150 oocytes per lane was loaded on the gel and accumulation of α-tubulin was used as a loading control. The complete time course was performed once, while changes between the first and last time point confirmed in two additional independent experiments (see Supplementary Fig 1a). **b** Morpholino oligonucleotide (MO) down-regulation of DAZL protein. GV stage oocytes from *RiboTag*^*fl/fl*^*:Zp3-Cre:WT* or *RiboTag*^*fl/fl*^*:Zp3-Cre:DAZL*^*+/−*^ mice were injected with CON-MO or DAZL-MO as described in the methods. After 6 h, oocytes were collected and used for Western blot analysis. A representative experiment of the three performed is reported. **c**–**e** GV stage oocytes from *RiboTag*^*fl/fl*^*:Zp3-Cre* or *RiboTag*^*fl/fl*^*:Zp3-Cre:Dazl*^*+/−*^ mice were injected with CON-MO or DAZL-MO and preincubated overnight in 2 µM milrinone, then cultured in inhibitor-free medium for maturation. Oocytes were collected at 0 and 6 h for RiboTag IP followed by qPCR analysis. Ribosome loading of the endogenous *Dazl* mRNA (unpaired two tailed *t*-test, *p* = 0.0022) and *Tex19.1* (unpaired two tailed *t*-test, *p* < 0.0001), but not *CcnB1* (unpaired two tailed *t*-test, *P* = 0.8567) mRNA is disrupted after DAZL depletion. (GV: germinal vesicle; MI: Meiosis I. Each dot represents an independent biological sample collected from different experiments done in different days. **f**–**h** Input data for the immunoprecipitation reported in **c**–**e**. When *N* > 2, data are presented as Mean ± SEM and each dot represents an independent biological observation. Statistical significance among the different samples was evaluated with the Brown-Forsythe ANOVA test, assuming unequal SD. Calculated *P* values: Dazl = 0.6680, Tex19.1 = 0.9713, CcnB1 = 0.5404.

We reported that DAZL protein is detectable in full-grown GV-arrested oocytes with protein levels increasing up to MII^21^, which is at odds with the data of others where DAZL protein was only borderline detectable or undetectable^23,24^ in full-grown mouse oocytes. We re-evaluated the expression of DAZL during maturation using a newly developed monoclonal DAZL antibody (see “Methods”). Western blot analysis of extracts from oocytes at different stages of maturation (0, 2, 4, 6, 8 h) reveals an immunoreactive polypeptide with mobility corresponding to that of DAZL (37 kDa) in GV oocytes, with a three-to-four fold increase in protein levels up to 8 h. of in vitro maturation (MI) (Fig. 1a, Supplementary Fig. 1a). This finding complements our previous reports using in vivo matured oocytes^21^. Note that the identity of the immunoreactive band is further confirmed by morpholino knockdown experiments (see Fig. 1b, Supplementary Fig. 1b). In addition, a progressive shift in mobility of the polypeptide on SDS-PAGE was frequently observed (Fig. 1a). Thus, two different antibodies document that DAZL is expressed in GV oocytes and accumulation of the protein increases with maturation. These conclusions are consistent with our *Dazl* mRNA translation data with in vivo and in vitro matured oocytes (Supplementary Fig. 1c–e).

To determine whether preventing *Dazl* mRNA translation effectively depletes the oocytes of the DAZL protein, GV-arrested oocytes from *RiboTag*^*fl/fl*^*:Zp3-Cre:Dazl*^*+/+*^ or *Dazl*^*+/−*^ mice were injected with a scrambled (CON-MO) or DAZL targeting morpholino (DAZL-MO), respectively, to maximize DAZL protein removal. Blockage of *Dazl* mRNA translation by this specific MO markedly reduces (94.23% decrease ± 0.025, Mean ± SEM, *N* = 3) the endogenous DAZL protein expression compared to a CON-MO (Fig. 1b, Supplementary Fig. 1b). To further assess the effectiveness and specificity of the treatment, we used RiboTag IP/qPCR to evaluate the MO effect on ribosome loading onto endogenous mRNAs during the transition from germinal vesicle stage (GV) to Meiosis I stage (MI). We observed a significant decrease in ribosome recruitment onto the *Dazl* mRNA, confirming the effectiveness of the MO in blocking initiation of translation with consequent depletion of the protein from oocytes (Fig. 1c). We show that mRNA loading onto ribosomes of *Tex19.1*, an established target of DAZL^22^, is significantly decreased after injection of DAZL-MO at the MI stage (Fig. 1d). Conversely, knockdown of DAZL had no effect on ribosome loading onto the non-target *CcnB1* (Fig. 1e)—consistent with our previous report^21,22^. No detectable effect on total transcript levels was detected under these conditions (Fig. 1f–h). Confirming our previous reports, DAZL depletion disrupts oocyte maturation to MII (see below). Further control experiments where immunoprecipitation was performed with WT rather than RiboTag mice yield only background signal – confirming the specificity of the RiboTag immunoprecipitation (Supplementary Fig. 1f). These pilot experiments document that DAZL knockdown specifically disrupts DAZL target loading onto ribosomes with high selectivity. Additionally, it validates that RiboTag IP depleted of DAZL in oocytes is an effective strategy to assess the role of this RBP in endogenous maternal mRNA translation.

### Ribosome loading onto mRNAs is disrupted in oocytes depleted of DAZL

**Fig. 2:**
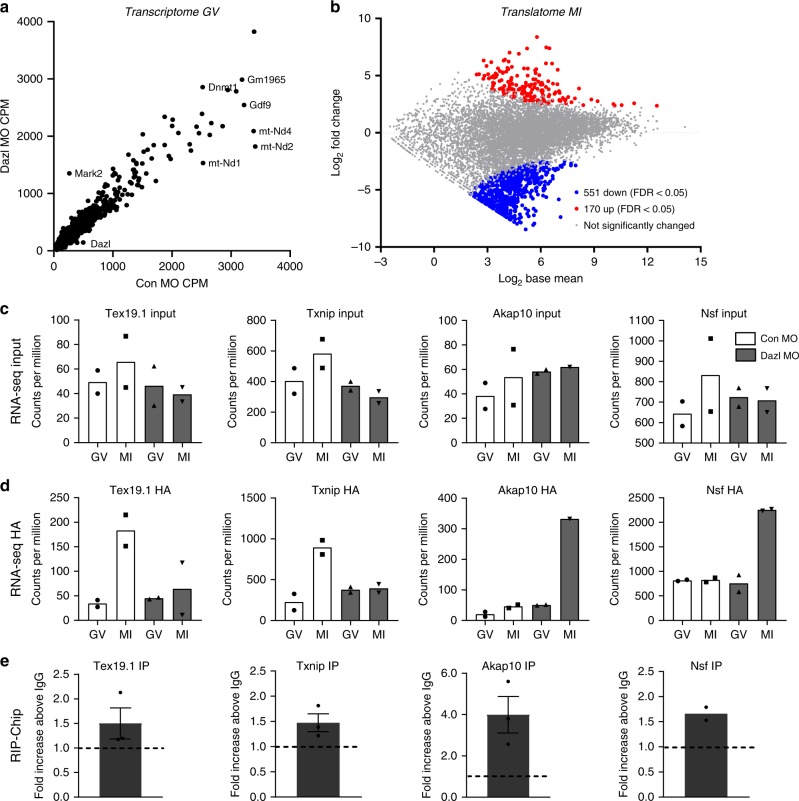
Maternal mRNA loading onto ribosome is disrupted in oocytes depleted of DAZL. **a** Comparison of transcriptomes of MO injected oocytes. Oocytes from *RiboTag*^*fl/fl*^*:Zp3-Cre:WT* mice were injected with a CON-MO whereas oocytes from *RiboTag*^*fl/fl*^*:Zp3-Cre:Dazl*^*+/−*^ mice were injected with a DAZL-MO. Oocytes were preincubated overnight in the presence of milrinone and the following morning were collected for RiboTag IP/RNA-Seq as described in the “Methods”. The average counts per million reads (CPM) for input (total transcripts) from two biological replicates is reported. **b** Comparison of transcripts recovered by RiboTag IP/RNA-Seq in Control and DAZL-MO injected oocytes (MI). GV stage oocytes from *RiboTag*^*fl/fl*^*:Zp3-Cre:WT* or *RiboTag*^*fl/fl*^*:Zp3-Cre*:*Dazl*^*+/−*^ mice were injected with CON-MO or DAZL-MO. After overnight preincubation with 2 µM milrinone, oocytes were cultured in inhibitor-free medium to allow reentry into the meiotic cell cycle. Oocytes were collected at 6 h for RiboTag IP and RNA-Seq analysis as detailed in the methods. Ribosome loading of significantly decreased transcripts (551 transcripts; FDR < 0.05, 2-fold change) is reported in blue, while ribosome loading of transcripts significantly increased (170 transcripts; FDR < 0.05, 2-fold change) after DAZL removal is reported in red. Transcripts not significantly changed are depicted in grey. Two independent biological samples were used in this analysis. **c**–**e** Effect of DAZL depletion on total RNA levels and ribosome loading of representative DAZL interacting targets. **c**, **d** GV stage oocytes from *RiboTag*^*fl/fl*^*:Zp3-Cre:WT* or *RiboTag*^*fl/fl*^*:Zp3-Cre:Dazl*^*+/−*^ mice were injected with CON-MO or DAZL-MO. The experimental conditions and data from analysis are as in **b**. The bar represents the mean and each dot represents the CPM from the two biological replicatesRNA-Seq data from supernatants (input) before IP (**c**) and RiboTag IP of CON-MO and DAZL-MO (**d**) are reported. **e** DAZL RIP-Chip of oocyte extracts of selected mRNAs is reported as the fold enrichment DAZL AB/IgG; Data are presented as mean values ±SEM of three biologically independent samples except for NSF1 where *N* = 2.

For genome-wide analysis of the effect of DAZL depletion on translation of oocyte endogenous mRNAs, GV oocytes from *RiboTag*^*fl/fl*^*; Zp3-cre:Dazl*^*+/+*^ or *Dazl*^*+/−*^ mice were injected with CON-MO or DAZL-MO. After overnight recovery, oocytes were collected at 0 h (GV) or cultured in inhibitor-free medium to mature for up to 6 h (MI). Although the changes in translation would be more pronounced if measured in fully matured MII oocytes, this shorter maturation time was selected to monitor early effects of DAZL depletion, thus avoiding the potential confounding effects of the blockage of maturation to MII and a potential decrease in oocyte viability. When we compare total mRNAs from CON-MO and DAZL-MO in GV-arrested oocytes (overnight incubation with PDE inhibitors), few differences are detected (Fig. 2a). Also, comparison of ribosome loading in the CON-MO at 0 and 6 h shows changes in ribosome loading qualitative similar to those reported previously with polysome array or other RiboTag IP/RNA-Seq data sets with non-injected oocytes (Supplementary Fig. 2a). Conversely, comparison of the RiboTag IP/RNA-Seq data in the 6 h DAZL-MO versus 6 h CON-MO group displayed complex changes in maternal mRNA ribosome loading (Fig. 2b). Although ribosome loading onto the majority of transcripts present in the oocyte is not significantly affected by DAZL depletion (grey dots in Fig. 2b), we detect a decrease in ribosome loading onto a group of transcripts (blue dots in Fig. 2b, 551 transcripts, >2-fold change, FDR < 0.05) (Supplementary Data 1)—a finding consistent with the established theory that DAZL functions as a translational activator. Surprisingly, we identified a group of transcripts whose translation increases (red dots in Fig. 2b, 170 transcripts, FDR < 0.05) after DAZL removal. This finding indicates that DAZL has a role in repression of translation, either directly or indirectly. As an example of the RNA-Seq data, ribosome loading onto *Tex19.1*, *Txnip*, *Akap10*, and *Nsf* mRNAs at 0 and 6 h of maturation are reported in Fig. 2c, d. We found no clear pattern in the changes of total mRNA levels after DAZL depletion (Fig. 2c). RiboTag IP/RNA-Seq shows an increase in ribosome loading (HA immunoprecipitation) for transcripts *Tex19.1* and *Txnip* during maturation in CON-MO injected oocytes, whereas the recruitment is blunted after DAZL KD in MI stage (Fig. 2d). Conversely, ribosome loading onto *Akap10* and *Nsf* mRNAs is increased after DAZL depletion (Fig. 2d). These data provide an initial indication that DAZL functions not only as a translational activator, but also a translational repressor during the GV-to-MI transition. These mRNAs are significantly immunoprecipitated by DAZL antibody as shown in our DAZL RIP-Chip Supplementary Data 2 (Fig. 2e) (see below). Knowing that no significant differences in total mRNA were detected between the CON-MO and DAZL-MO groups, calculation of the translational efficiency (HA-IP:input ratio) shows the same trend (Supplementary Fig. 2b).

### RiboTag IP/qPCR of candidate mRNA confirms Dazl dual action

**Fig. 3:**
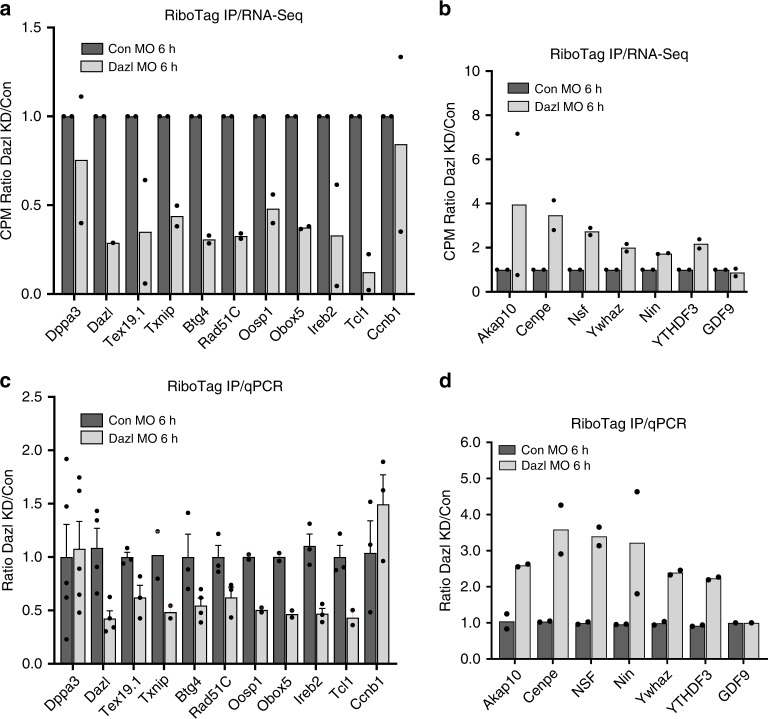
RiboTag IP/qPCR confirms the presences of a subset of transcripts whose translation is upregulated and downregulated in oocytes depleted of DAZL. Representative mRNA targets affected by DAZL removal. RiboTag/RNA-Seq data are reported in **a** down and **b** up. Ribosome loading DAZL-MO/CON-MO for the same transcripts assessed in independent biological replicates by RiboTag IP/qPCR is reported in **c**, down and **d** up. Data are reported as ratio DAZL KD/control. *Dppa3* and *CcnB1* are used as negative control in **a**, **c**. *Gdf9* mRNA is used as negative control in **b**, **d**. Experimental conditions are identical to those described in Fig. 2b except for the qPCR analysis. Bars are the average of two or more observations. *P* values in panel C were calculated with the Bonferroni-Dunn method, with alpha = 0.05. Each gene was analyzed individually, without assuming a consistent SD. *P* values are the following: Dppa3 = 0.851918, Dazl = 0.015042, Tex19.1 = 0.035962, Btg4 = 0.069233, Rad51C = 0.059755, Ireb2 = 0.006635, Tcl1 = 0.032710, CcnB1 = 0.327854.

To confirm the opposing effect of DAZL depletion on translation, we performed RiboTag IP/qPCR with independent biological samples to monitor the recruitment of representative transcripts to the ribosome/translation pool. GV stage oocytes *RiboTag*^*fl/f*^*;Zp3-Cre;Dazl*^*+/+*^, or *Dazl*^*+/−*^ mice were injected with CON-MO or DAZL-MO and processed following the same protocol of the RNA-Seq experiment. The RiboTag IP/qPCR RNA quantification confirms that the overall transcripts levels (input of RiboTag IP/qPCR) are not affected, including transcripts coding for *Dazl*, *Tex19.1*, *Txnip*, *Rad51C*, *Btg4*, *Ero1, Oosp1*, *Obox5*, *Ireb2*, and *Tcl1* (Supplementary Fig. 3a). However, RiboTag IP/qPCR shows a decrease in translation for these candidates after DAZL removal, similar to that observed with the RiboTag IP/RNA-Seq (Fig. 3a, c). *Dppa3* and *CcnB1* are used as negative controls, as they are not regulated by DAZL during oocyte maturation^21,22^. Conversely, translation of transcripts coding for *Akap10*, *Cenpe*, *Nsf*, *Ywhaz*, *Nin*, and *YTHDF3* is upregulated after DAZL removal (Fig. 3b, d), while the overall transcript levels are not changed (Supplementary Fig. 3b). *Gdf9*, used as negative control, is not affected by DAZL depletion. These results confirm our RiboTag IP/RNA-Seq data, indicating that DAZL may play a dual function (both translational activator and repressor) during oocyte maturation.

### DAZL interacts with mRNAs whose translation is disrupted

**Fig. 4:**
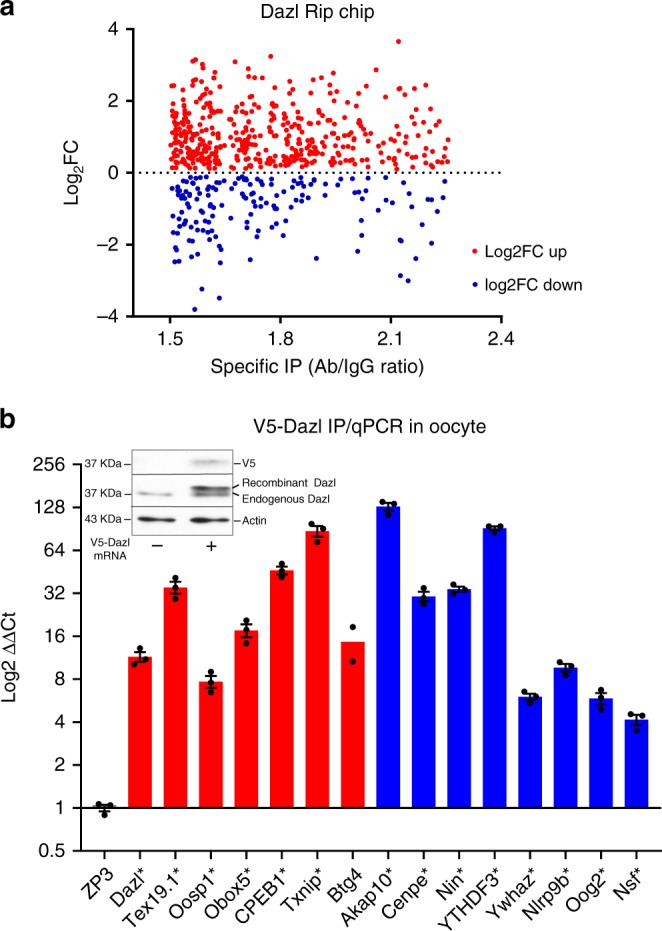
DAZL physically interacts with transcripts whose translation is upregulated or downregulated during oocyte maturation. **a** Comparison DAZL Rip-Chip and polysome array in oocytes. Changes in ribosome loading from 0 h (GV) to 16 h (MII stage) is reported in the y axes, while the interaction with DAZL assessed by RIP-Chip is reported in the X axes. Subset of transcripts whose translation increased from GV to MII stage are also enriched in DAZL immunoprecipitates of oocyte extracts (red); transcripts whose translation decreases during oocyte maturation and specifically immunoprecipitated by DAZL antibody (blue). The GV/MII ribosome loading data are those published in ref. ^21^. For DAZL RIP-Chip, oocytes were obtained from mice primed with PMSG and hCG. Oocyte lysates was immunoprecipited with DAZL-specific antibody or IgG and the mRNA recovered in the IP pellet measured by microarray hybridization. Each dot is the average of three independent biological replicates (**b**) Interaction of DAZL with selected targets upregulated or downregulated in oocytes after DAZL depletion. Oocytes were injected with a V5-tagged *Dazl* construct. After overnight incubation in milrinone, oocytes were extracted and an aliquot was used for WB (inset) or used for RIP/qPCR. Data are presented as bars with means ±SEM of *n* = 3 biologically independent experiments except for Btg4 where *N* = 2.

The above findings open the possibility that DAZL binding to maternal mRNA leads to an increase and decrease in translation. If true, DAZL should equally bind to maternal mRNAs, whose translation is upregulated or downregulated during oocyte maturation. To test this hypothesis, we compared a previously generated DAZL RIP-Chip dataset to our published GV/MII dataset^21^. In the DAZL RIP-Chip dataset, 847 transcripts are significantly immunoprecipitated by DAZL antibodies with a cutoff of >1.5 fold enrichment and *P* < 0.05, as compared to IgG during the GV to MII transition. A scatter plot (Fig. 4a) of this data shows DAZL binding to upregulated transcripts and downregulated transcripts. To verify whether the mRNAs recovered in the RIP pellet possess consensus sequences known to interact with DAZL, we performed an in silico scan of these 3′UTRs for the known consensus sequences defined by Selex^25^, DAZL crystal structure^26^, or by Clip/seq^19^. Of the 677 transcripts in the RIP datasets for which a 3′UTR could be identified, 637 sequences (94%) recovered in the IP pellet contain at least one consensus sequence, consistent with a direct Dazl interaction.

Next, we determined whether the mRNAs whose translation is positively and negatively affected by DAZL depletion contain DAZL consensuses in the 3′UTR. Since the minimal DAZL consensus sequences is common, we used the most stringent conditions possible by testing the null hypothesis. We determined whether a subgroup of transcripts that contain no discernible DAZL element is affected by DAZL depletion. We identified 66 transcripts with no DAZL consensus whose translation is downregulated (2-fold change and FDR < 0.05) and 8 transcripts whose translation is upregulated (2-fold change and FDR < 0.05) (Supplementary Fig. 4). These findings confirm that a small subset of transcripts is indirectly affected by DAZL depletion. Nevertheless, the majority of the 3′UTRs that are positively or negatively affected by DAZL depletion (597 out of 671 transcripts with identifiable 3′UTR) contain DAZL consensus sequences (Supplementary Fig. 4). Similarly, transcripts affected by Dazl depletion are recovered in the IP pellet (Supplementary Fig. 5). Of note, a gene ontology analysis of the genes affected by DAZL depletion shows an enrichment in transcripts coding for ribonucleoproteins or transcripts involved in translational regulation (Supplementary Fig. 6), consistent with possible indirect effects via Dazl control of downstream translational regulators.

Finally, we wished to independently confirm that candidate transcripts whose translation increases or decreases after DAZL depletion are indeed direct targets of DAZL. Because no sufficient signal could be obtained in RIP when using 200 oocytes with currently available DAZL antibodies, we have expressed a V5-tagged DAZL protein in the oocyte. We chose concentrations of the mRNA that afford expression of the recombinant protein at levels comparable to the endogenous DAZL (see inset Fig.4b). This recombinant tagged protein is efficiently immunoprecipitated by a V5 antibody. When a RIP assay is performed in extracts from oocytes injected with this tagged Dazl, we observe specific immunoprecipitation of *Dazl, Tex19.1, Oosp1, Obox5, Btg4, CPEB1, and Txnip* transcripts (Fig. 4b, red), whose translation is downregulated by DAZL removal, but also transcripts coding for *Akap10, Cenpe, Nin, Ytdf3, Ywhaz, Nsf, Nlrp9b and Oog2*, whose translation is upregulated by DAZL removal (Fig. 4b, blue). Zp3 mRNA was used as a negative control. Similar Dazl interactions were observed if we used extracts of mouse ES cells that express endogenous DAZL at high levels for the RIP/qPCR^18,27,28^ (see Supplementary Fig. 7).

### The 3′UTR of target mRNAs recapitulates the DAZL effects

**Fig. 5:**
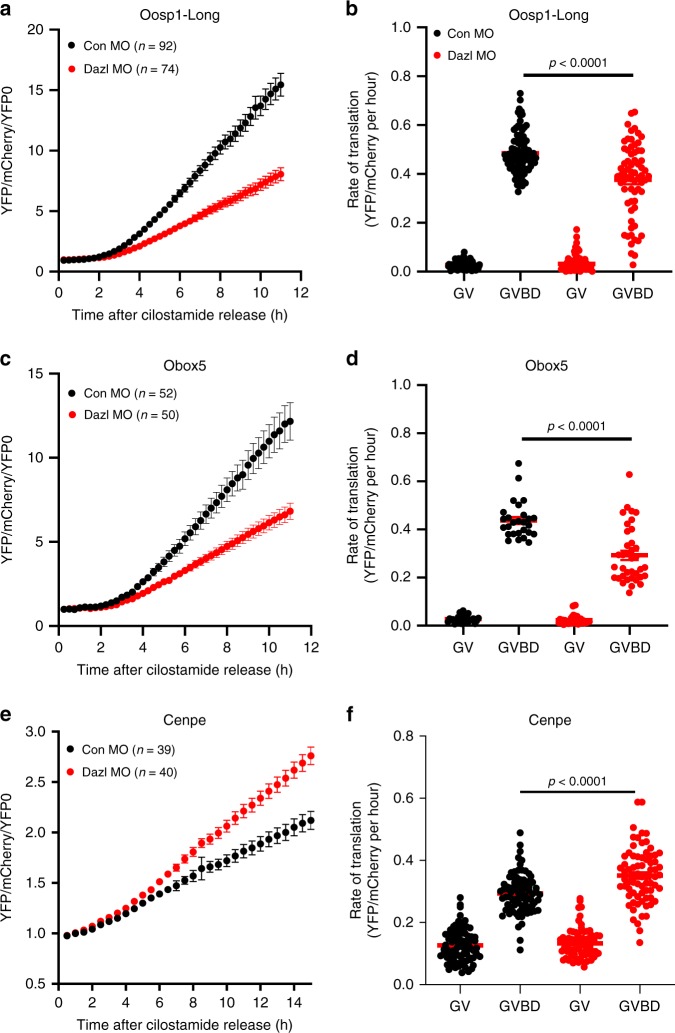
The 3′ UTR of Oosp1, Obox5 and Cenpe recapitulates the effect of DAZL depletion on endogenous mRNA translation. Oocytes were co-injected with ployadenylated *mCherry* mRNA and oligoadenylated *YFP-Oosp1 3*′*UTR*, *YFP-Obox5 3*′*UTR*, or *YFP-Cenpe 3*′*UTR* reporters with either CON-MO or DAZL-MO. Oocytes were then pre-incubated overnight to allow the mCherry signal to reach a plateau. At the end of the preincubation, oocytes were released in cilostamide-free medium for maturation and YFP and mCherry signal were recorded by time lapse microscopy. The YFP signal was analyzed using Metamorph software (version 7.8.13.0) corrected by the level of coinjected mCherry signal and normalized to the first recording of YFP/mCherry. Experiments were repeated at least three times and the data are the cumulative mean ± SEM of the three or more independent experiments. Individual oocyte YFP/mCherry signals were used to calculate the rate of translation of the reporters at the 0–2 h (prior to GVBD) and 4–8 h (after GVBD) as detailed in the Methods. The differences between rates of *YFP-Oosp1* (**b**) *YFP-Obox5* (**d**) and *YFP-Cenpe* (**f**) were analyzed by unpaired two tailed *t* test.

*Oosp1* and *Obox5* are two oocyte-specific transcripts whose translation is decreased by DAZL removal as determined by both the RiboTag IP/RNA-Seq and the RiboTag IP/qPCR. Using the same criteria, the mRNA coding for the centromeric protein *Cenpe* is upregulated. OOSP1 (oocyte secreted protein 1) was initially identified as an abundant novel oocyte-secreted protein^29^. It is expressed in the oocytes with two short and long 3′ UTRs generated by alternative cleavage and polyadenylation^21^. OBOX5 (oocyte-specific homeobox 5) is a member of the OBOX family of proteins but its function remains unclear^30^. To verify the effect of DAZL on translation of these candidate mRNAs, a YFP reporter was fused to the *Oosp1, Obox5, or Cenpe* 3′UTR and these constructs were injected in oocytes together with either CON-MO or DAZL-MO. A fully polyadenylated *mCherry* reporter was used as a control of the volume injected. The accumulation of YFP and mCherry in individual oocytes was recorded throughout meiotic maturation and YFP signals were corrected by the level of co-injected *mCherry*. Data are expressed as changes over 0 h (GV stage), as differences in reporter accumulation were detected in GV-arrested oocytes (see below). By measuring the average YFP signals throughout maturation, the accumulation of *YFP-Oosp1* and *YFP-Obox5* reporter in CON-MO group closely follows the corresponding ribosome loading onto the endogenous mRNA; DAZL depletion causes at least 50% decrease in translation in *Oosp1* and *Obox5* reporter during oocyte maturation (Fig. 5a, c). Conversely and upon DAZL depletion, the translation of the *Cenpe* reporter was upregulated during oocyte maturation (Fig. 5e). We further assessed the rates of translation during oocyte maturation of the reporters by fitting the YFP/mCherry data during GV (0–2 h) and after GVBD (4–8 h.) (Fig. 5b, d). We found a significant decrease of *Oosp1* (*p* < 0.0001) and *Obox5* (*p* < 0.0001) translation rates in DAZL-MO injected oocytes (Fig. 5b, d), as well an increase in *Cenpe* reporter rates of translation (p < 0.0001) (Fig. 5f), confirming that DAZL depletion affects the translation of these reporters during maturation. Consistent with our RiboTag IP/RNA-Seq data (Supplementary Fig. 8a–f), the translational efficiency of *Oosp1*, *Obox5* and *Cenpe* is affected by DAZL depletion (Supplementary Fig. 8g–i). *CcnB1* 3′UTR co-injected with either CON-MO or DAZL-MO shows no obvious changes in translational accumulation between the two groups, confirming the selective effect of the DAZL depletion (Supplementary Fig. 9a, b). Thus, the reporter assay with the *Cenpe* 3′UTR, as well as the experiments with the *Oosp1-short* 3′UTR (see below), confirm that DAZL functions also as repressor of translation during oocyte maturation.

In a gain-of-function paradigm, to confirm that DAZL interaction with a 3′UTR is sufficient to modulate translation during maturation, we generated a synthetic 3′UTR where the only element present is a PAS element. Expression of reporter driven by this synthetic 3′UTR is translated at a low rate in oocytes during maturation. However, if a tandem of DAZL elements is inserted in the sequence, that translation increases significantly (Supplementary Fig. 10).

**Fig. 6:**
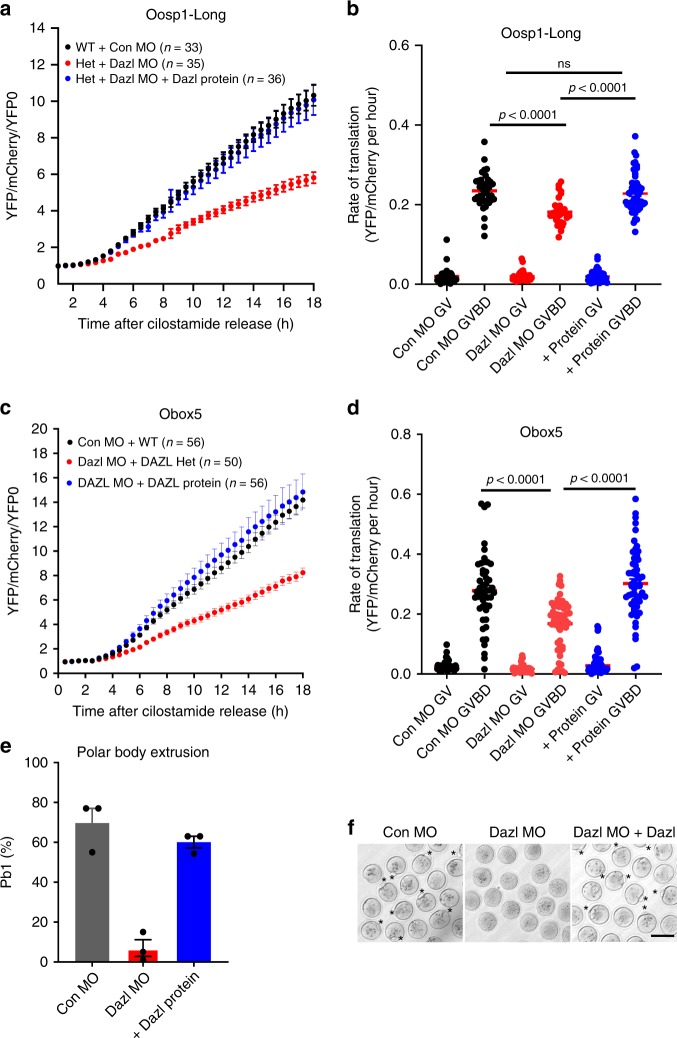
The translation of the YFP-Oosp1 or YFP-Obox5 reporter in the DAZL-depleted oocytes is rescued by injection of DAZL protein. **a** Human DAZL protein injection restores *Oosp1* translation during oocyte maturation in MO injected oocytes. Oocytes were injected with *mCherry* mRNA and *YFP-Oosp1 3*′ *UTR* reporter with either CON-MO or DAZL-MO, with or without recombinant human DAZL protein, and incubated in cilostamide containing medium overnight to allow mCherry signal to reach a plateau. At the end of the preincubation, oocytes were released in cilostamide-free medium for maturation and YFP and mCherry signal recorded by time lapse microscopy. YFP signal were corrected by the level of co-injected mCherry signal and were normalized to the first time point. Experiments were repeated three times and the data are the mean ± SEM of three independent biological samples. **b** Rates of translation measured at 0–2 h and 6–10 h of maturation for the time courses reporter in **a**. Statistical analysis was done by unpaired two tailed *t*-test, *p* < 0.0001). **c**, **d** Similar experiments done the with Obox5 reporter. Data in **d** were analyzed by unpaired two tailed *t*-test, *p* < 0.0001). **e** Microinjection of a human DAZL protein rescues the meiotic block of oocytes injected with DAZL- MO. Bars are the mean ± SEM from three biologically independent experiments. Significance was calculated by unpaired two tailed *t*-test, *p* < 0.0001). **f** Representative Image of oocytes from the three conditions reported in e, at least *n* = 3 biologically independent experiments were performed. Oocytes maturation was scored by counting the number of oocytes with a polar body. Scale bar = 100 µm.

To verify whether the depletion of DAZL protein with the specific MO is the sole cause of the decreased translation of the reporter, we performed the following rescue experiment. A human recombinant DAZL protein was injected together with the DAZL-MO and the *Oosp1* or *Obox5* reporter. As observed above, DAZL depletion causes a decrease in the rate of translation of the two reporters. This decrease in translation of both reporters is completely rescued when the recombinant DAZL protein is co-injected with the DAZL-MO (Fig. 6a–d). The rescue effect of the DAZL protein was not limited to the translation efficiency. As previously reported, DAZL depletion on a heterozygous background almost completely prevents oocyte maturation to MII (CON-MO: 69.7% versus DAZL-MO: 7%). Conversely, when the DAZL-MO is co-injected with a recombinant DAZL protein, oocytes complete maturation to MII at a rate (63%) similar to control injected oocytes (Fig. 6e, f). Taken together with the RiboTag IP/RNA-Seq and qPCR data (Fig. 2, Fig. 3), the reporter measurements further support the conclusion that DAZL protein plays a role in the translational activation of these three targets mRNAs, that the 3′UTR of these mRNAs mediates the effect of this RBP on translation, and that DAZL depletion is the cause of the altered translation.

### DAZL depletion upregulates translation in GV-arrested oocytes

**Fig. 7:**
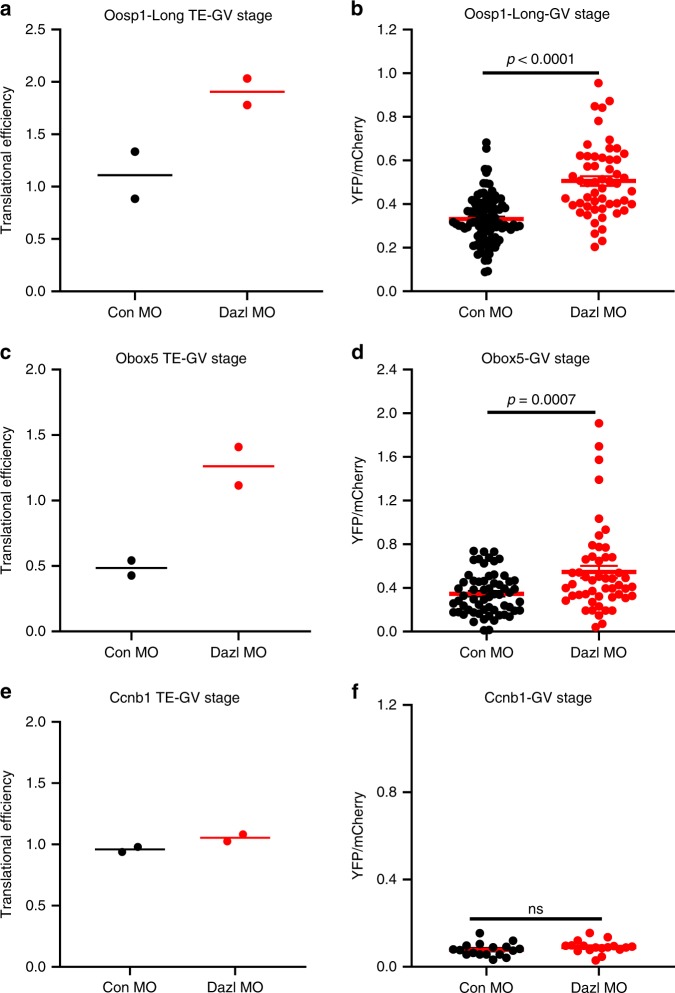
DAZL depletion increases ribosome loading of Oosp1 and Obox5 endogenous transcripts and translation of the Oosp1 and Obox5 reporters in GV-arrested oocytes. **a**, **c**, **e** GV stage oocytes from *RiboTag*^*fl/fl*^*:Zp3-Cre:WT* or *RiboTag*^*fl/fl*^*:Zp3-Cre:Dazl*^*+/−*^ mice were injected with CON-MO or DAZL-MO. Oocytes were pre-incubated overnight with 2 µM milrinone. In all, 0 h (GV stage) data from RiboTag IP/ RNA-Seq were used to calculate the Translational Efficiency (TE). TE is calculated as the ratio of the CPM for HA immunoprecipitated transcripts *Oosp1* or *Obox5* over the corresponding input at 0 h. The TEs are reported for *Oosp1* (**a**) and *Obox5* (**c**) or *CcnB1* (**e**). Data are presented as mean of two biologically independent experiments for **a**, **c**, **e**. **b**, **d**, **f** GV stage oocytes were injected with *mCherry*-polyadenylated mRNA and *YFP-*3′*UTR* reporter for *Oosp1* 3′ UTR or *Obox5* 3′UTR with either CON-MO or DAZL*-*MO. Oocytes were pre-incubated overnight to allow mCherry signal to plateau, then further incubated in cilostamide-containing medium. YFP signal were corrected by the level of coinjected mCherry signal. Reporters injected are: **b** *Oosp1* (unpaired two tailed *t*-test, *p* < 0.0001), **d** *Obox5* (unpaired two tailed *t*-test, *p* = 0.0007) and **f** *CcnB1* (unpaired two tailed *t*-test, *p* = 0.2274). Data are presented as mean values ± SEM of rates for each individual oocytes from *n* = 3 independent biologically experiments for **b**, **d**, **f**.

In the above experiments on reporter translation, we consistently observed that translation of *Oosp1* and *Obox5* reporters during the first two hours of incubation is significantly increased in the DAZL-MO injected group when the oocytes were still GV-arrested (*Oosp1*: *p* < 0.0001 and *Obox5*: *p* = 0.0007) (Fig. 7b, d). To verify this apparent de-repression in DAZL-depleted, quiescent oocytes, we reanalyzed the RiboTag IP/RNA-Seq data sets. We found ribosome loading on both Obox5 and *Oosp1* during GV-arrested is also increased in this dataset (Supplementary Fig. 11). To remove possible bias due to variation in the total mRNA, we calculated the translational efficiency (TE) of these two transcripts after DAZL depletion. Indeed, the TEs for *Oosp1* and *Obox5* significantly increased in GV oocytes depleted of DAZL (Fig. 7a, c), whereas *CcnB1* translation is not affected (Fig. 7e, f). To assess whether this effect of DAZL depletion is widespread, we reanalyzed the RiboTag IP/RNA-Seq data and found that ribosome loading of ~153 transcripts is significantly increased after DAZL removal in GV oocytes (Supplementary Fig. 11 red, FDR < 0.05).

As an alternative strategy to test the repressive function of DAZL in GV oocytes, we coexpressed the *Oosp1* reporter with increasing concentrations of tagged *Dazl* mRNA (Supplementary Fig. 12). This co-expression leads to a decreased translation of the reporter. At a concentration of 15 ng/ml, the repression of translation is evident if a DAZL element is present in the 3′UTR of the reporter. If this element is mutated to prevent DAZL binding, translational repression is no longer detected. Thus, both of loss-of-function or gain-of-function experiments are consistent with a repressive function of DAZL on specific 3′UTRs.

### DAZL cis-acting elements are required for DAZL action

**Fig. 8:**
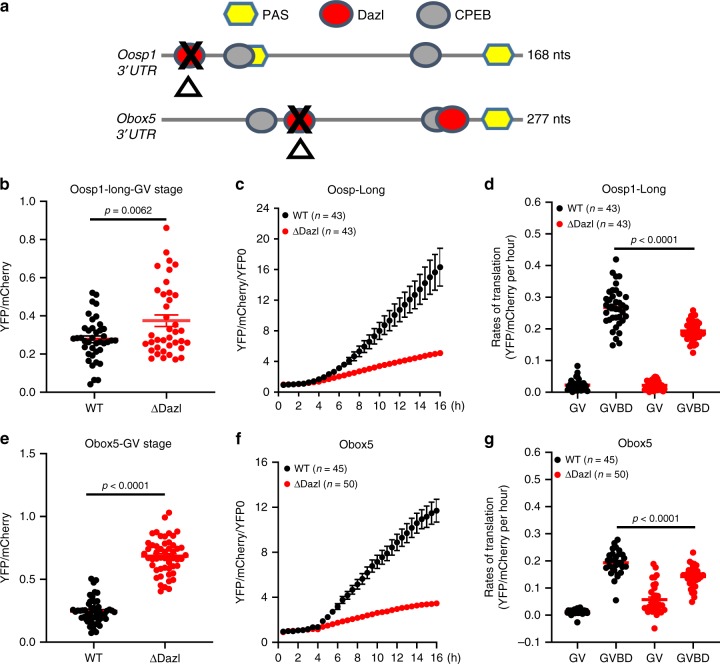
Translation of Oosp1 and Obox5 reporter is dependent on the presence of a DAZL binding element. Wild type *Oosp1 3*′*UTR* or *Obox5 3*′*UTR* as well as constructs with mutations in the DAZL binding site were injected along with *mCherry*-polyadenylated mRNA into GV stage oocytes. After overnight pre-incubation to allow mCherry signal to plateau, oocytes were released in cilostamide-free medium and signals recorded. YFP signal were corrected by the level of co-injected mCherry signal. Black symbols: control; Red symbols: mutant constructs. Experiments were repeated three times and the number of oocytes recorded is reported. **a** Scheme of the *Oosp1* and *Obox5* 3′ UTR and position of the PAS, putative CPEB1 and DAZL-binding elements. Mutagenesis of the putative DAZL-binding element was performed as detailed in “Methods” section. A red oval represents the DAZL consensus sequence in the 3′UTR of *Oosp1* and *Obox5*. A black cross indicates the mutated DAZL-binding consensus sequence. **b**, **e** The effect of DAZL-binding element mutation on *Oosp1* (unpaired two tailed *t*-test, *p* = 0.0062) or *Obox5* (unpaired two tailed *t*-test, *p* < 0.0001) translation in GV stage. Rates of reporter accumulation were calculated for each GV-arrested oocyte and plotted as individual dots. Mean and SEM values were calculated from three independent experiments. **c**, **f** Mutation of DAZL-binding element on 3′UTR of *Oosp1* or *Obox5* decreases translation of reporters during oocyte maturation. YFP signals were corrected by the co-injected mCherry signal. Each time point was normalized to the first YFP:mCherry ratio. **d**, **g** Rates of *YFP-Oosp1* or *YFP-Obox5* reporter accumulation compared to a wild type reporter calculated from **c**, **f** (unpaired two tailed *t*-test, *p* < 0.0001).

Collectively, the experiments strongly indicate that translational regulation of *Oosp1* and *Obox5* transcripts are affected by DAZL-MO injection during GV to MI transition. To test if this effect is due to DAZL binding to these target mRNAs, we mutated the DAZL-binding sites (UU[G/C]UU) by replacing critical nucleotides with adenosine in *Oosp1* 3′UTR or *Obox5* 3′UTR. This mutation was shown to disrupt DAZL binding^22,26^. A schematic representation of *Oosp1* and *Obox5* 3′UTR with mutated DAZL binding sequence is reported in Fig. 8a. When YFP reporters tagged with mutant *Oosp1* 3′UTR or *Obox5* 3′UTR were injected in oocytes with their rates of translation monitored during maturation, mutation of a single DAZL-binding site in either *Oosp1* or *Obox5* 3′UTR significantly decrease the rate of reporter accumulation (Fig. 8c, d, f, g) during meiotic resumption. Mutation of the DAZL binding site in *Oosp1* (Fig. 8b) and *Obox5* (Fig. 8e) also cause increased translation in GV stage oocytes compared to wild-type reporters. Increased rate in reporter translation was confirmed in another experimental paradigm where control and DAZL-depleted oocytes were maintained in GV stage. Under these conditions, translation rate of the *Oosp1* reporter increased, whereas *CcnB1* mRNA is not affected (Supplementary Fig. 13). This latter finding is consistent with the results of DAZL-MO of the RiboTag IP/RNA-Seq experiment (Fig. 7a, c, e), further consolidating the hypothesis that DAZL functions as a translational repressor in GV-arrested oocyte.

### DAZL regulation depends on the context of the target 3′UTR

**Fig. 9:**
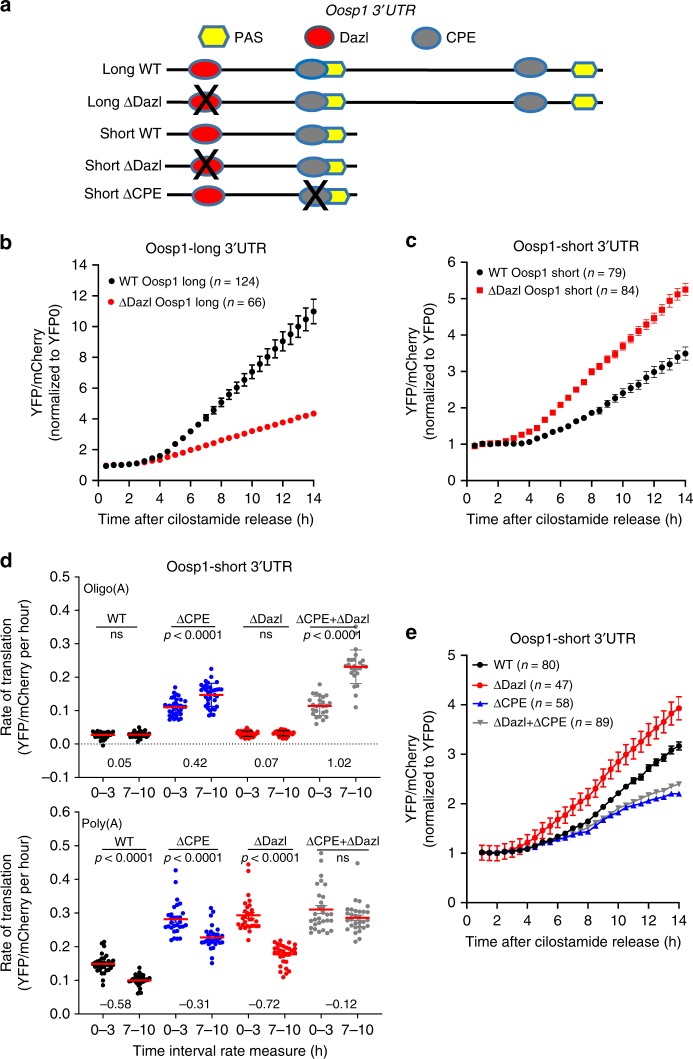
DAZL positive or negative effects on translation depend on the 3′UTR context and involve an interaction with the CPEB1-dependent adenylation. **a** Scheme summarizing the domain organization of the two long and short *Oosp1* variant 3′UTRs expressed in mouse oocytes. PAS: Cleave and polyadenylation signal; Dazl: consensus DAZL binding sequence; CPE: CPEB1 binding consensus sequence. The mutated element is marked by an X. **b**, **c** Comparison of the effect of DAZL element mutagenesis on the translational activation of *Oosp1* reporter during maturation. The *Oosp1* long and short wild type (WT, no mutation) and mutant (ΔDazl) reporters were generated by fusing the two 3′UTRs to a YFP sequence and were injected in GV oocytes together with the *mCherry* constitutive reporter. After overninght recovery, oocytes were transferred to maturation medium and fluorescence recorded by time lapse microscopy. Each experiment was repeated three or more times in different days and oocyte recordings were combined. Each point is the Mean ± SEM of single oocyte measurements. The total number of oocytes used for each construct is reported among brackets. **d** Effect of single or combined CPE and DAZL binding element mutagenesis on polyadenylation and translational rate of the *Oosp1-short* 3′UTR reporter. Oligo- or poly-adenylated wild type and mutant reporters were generated as described in the Methods and injected in GV oocytes. After 3 h recovery, fluorescent recordings were monitored by time lapse microscopy in oocytes maintained in GV. Rates of reporter translation were calculated for each injected oocyte at the beginning (0–3 h.) and at the end (7–10 h.) of the incubation (see Methods for details). The average ± SEM from two experiments was calculated for each group of measurements and the statistical significance was calculated by *T*-test with Welch correction. To estimate the adenylation or deadenylation of the reporter, the ratio between 0–3 h and 7–10 h was calculated for each oocyte and the log2 average reported below the measurements (Upper panel: unpaired two tailed *t*-test, WT *p* = 0.6985, ΔCPE *p* < 0.0001, ΔDazl *p* = 0.3536, ΔCPE + ΔDazl *p* < 0.0001; Lower panel: unpaired two tailed *t*-test, WT p < 0.0001, ΔCPE *p* < 0.0001, ΔDazl *p* < 0.0001, ΔCPE + ΔDazl *p* = 0.0912). **e** Consequences of single or combined mutation of the CPE and DAZL element on the translation of the *Oosp1-short* reporter during oocyte maturation. The experimental design is as in **c**. Each point is the mean ± SEM of single oocyte measurements from three independent experiments. The total number of oocyte used in the experiment is reported among brackets.

In-depth analysis of DAZL’s mode of regulation of *Oosp1* mRNA provides further insight on the duality of DAZL’s mode of action. The 3′UTR of *Oosp1* mRNA contains the above-mentioned DAZL element, two CPE elements, and two PAS elements (Fig. 9a). The presence of proximal and distal PAS predicts that Oosp1 mRNA is present in oocytes with two distinct short and long UTRs (Fig. 9a). Indeed, mRNAs’ presence with two 3′UTRs of varied lengths is verified by bioinformatic searches of ESTs expressed in the oocyte, revealing an additional boundary of cleavage downstream of the proximal PAS (Supplementary Fig. 14). Our previous bioinformatics scanning in oocytes with dynamic analyses of alternative polyadenylation (*DaPars* algorithm) also detected *Oosp1*’s mRNA with two distinct 3′UTRs^31^.

When YFP constructs driven by *Oosp1-short* and long 3′UTR are expressed in oocytes, both are repressed in GV and activated during maturation; however, the long form is activated more than the short. Injection of constructs with a deletion in the DAZL element detects an increase in translation for both short (Supplementary Fig. 15) and long (see Fig. 8b) constructs when the oocyte is maintained in GV, confirming the repressive function of DAZL in quiescent oocytes. However, when reporter accumulation is monitored during maturation, DAZL element deletion has opposing effects on translation of these two reporters (Fig. 9b, c). Translation of the long form with DAZL deletion is decreased during maturation, whereas translation of the DAZL mutant short form is consistently activated (*N* = 6 independent experiments). This finding supports DAZL’s duality function and indicates that the 3′UTR context, as well as the state of maturation of the oocyte, plays a determinant role on the repressive or activating translational outcome.

To obtain further insight into the mechanism of DAZL repression of translation, we used a novel strategy where the mutagenesis of RBP binding elements and translation of oligo-adenylated or poly-adenylated reporters are combined. If a reporter is repressed in the GV state, the translation rate of an oligo-adenylated probe remains constant^32^. If repression is relieved, as it happens when an RBP binding site is mutated, an increase in translation of oligo-adenylated probes becomes evident with time^32^. Moreover, forcing polyadenylation of a repressed reporter causes a transient increase in translation that is reversed over time because the reporter becomes de-adenylated^32^. Using this experimental paradigm, we find that combined mutagenesis of the DAZL and CPE element in *Oosp1-short* 3′UTR causes maximum increase in translation of the oligo-adenylated reporter (Fig. 9d top panel). Whereas translation of the double mutant does not significantly change if the reporter is artificially poly-adenylated (Fig. 9d, bottom panel). The increased translation of the oligo-adenylated double mutant indicates that preventing the DAZL and CPEB1 binding causes adenylation of the reporter and de-repression. This effect is absent when the reporter is already artificially poly-adenylated (Fig. 9d, bottom panel). Decreased translation of the reporter with time is detected in all other poly-adenylated constructs as it may become deadenylated and partially repressed (Fig. 9d, bottom panel). Since the effects in the double mutant are larger than in the single mutants, we argue for a synergism between the DAZL and CPE elements in exerting translational repression (Fig. 9d top panel).

When the interaction between DAZL and CPEB1 is monitored during maturation, activated translation of *Oosp1-short* form consequent to the DAZL element deletion is abolished if the CPE element is also mutated (Fig. 9e). Thus, repressive effects of DAZL on this *Oosp1-short* reporter continue during maturation and are exerted via inhibition of the CPEB1-activating function.

## Discussion

With the experiments described above, we demonstrate that the RNA Binding Protein DAZL plays a central role in translational regulation of maternal mRNAs in fully grown mouse oocytes. The DAZL protein is present and accumulates during oocyte maturation, and interfering with this accumulation causes a failure in oocyte progression to MII. Using different complementary strategies, we demonstrate that DAZL functions as both repressor and activator of translation, depending on the context of 3′UTR present in an mRNA. Moreover, we provide evidence that DAZL cooperates with CPEB1 in regulating translation of a subset of mRNAs. Given its overall repressive effects in GV quiescent oocytes, our findings establish that, like CPEB1, DAZL plays an important role in preventing translation of maternal mRNA synthesized during oocyte growth.

Our genome-wide analysis of ribosome loading onto mRNAs indicates that upon DAZL depletion a subset of maternal mRNA becomes translated at an increased rate in GV oocytes. In contrast, their translation in the absence of Dazl decreases after GVBD during maturation indicating that DAZL functions as a translational repressor in GV and as an activator after GVBD. Depletion of DAZL protein by MO or disruption of its binding to the translational reporters including *Obox5*, *Oosp1-Long* form and previously reported transcript *Tex19.1*^22^, summarizes this dual role of DAZL in prophase I and during oocyte meiotic reentry, implying a signal in the oocyte at the time of GVBD causes a switch in DAZL function from repressor to activator. During the GV-to-MI transition, two kinds of changes are observed for the DAZL protein. A shift in SDS-PAGE mobility of the DAZL immunoreactive polypeptide in Western blot suggests a post-translational modification of the protein. DAZL phosphorylation in the RMM domain by MAPK2 has been reported^33^; however, this phosphorylation suppresses the *Dazl* translational activity. If additional phosphorylation sites are used in oocytes, plausibly the de-phosphorylated DAZL is involved in recruitment of a repressive complex in GV oocytes, whereas phosphorylation of the protein either causes disassembly of this complex or promotes the formation of a new complex facilitating adenylation, ribosome recruitment, and translation. Although further exploration of the molecular details of this regulation is necessary, this mode of regulation would be identical to that observed for CPEB1. In frog oocytes arrested in prophase, a dephosphorylated CPEB1 assembles a complex that represses translation by maintaining mRNA deadenylation^34,35^; conversely, phosphorylation of CPEB1 by several kinases activated around GVBD causes disassembly of the repressive complex and formation of a new complex that promotes polyadenylation and increased translation^36^. The experiments combining mutagenesis and manipulation of the poly(A) in the *Oosp1* reporter are consistent with this view. Secondly, DAZL protein accumulation increases during oocyte maturation, opening the possibility that activated translation depends on the stoichiometry of DAZL interaction with an mRNA. It has been reported that multiple DAZL bindings to a 3′UTR increases the translation rate of a reporter, a property that we have confirmed for Tex19.1^22^. Moreover, insertion of two DAZL elements in a synthetic 3′ UTR causes an increased translation of the reporter, confirming that DAZL binding is necessary and sufficient to cause translational activation at GVBD. We have attempted to manipulate DAZL concentration in the oocytes by overexpression of a V5-tagged protein. In this paradigm, we can only detect the repressive function on translation associated with increased DAZL concentration. However, this experiment should be interpreted with some caution because it has been difficult to precisely titrate the DAZL protein concentration in the oocyte. In addition, the reporter used in these experiments contains only one DAZL binding site.

Our data documents the presence of a second subset of maternal mRNAs targets of DAZL (170, 2-fold change FDR < 0.05). As inferred from the MO experiments and the DAZL element mutagenesis, these mRNAs are repressed by DAZL both in GV and during GVBD. Together with the *Cenpe* reporter, the translational pattern of the *Oosp1*-short 3′UTR strongly supports this repressive behavior of DAZL. Disruption of the DAZL binding site in the context of the short 3′UTR of *Oosp1* causes an increased translation in GV as well as after GVBD. The combined mutagenesis of the DAZL and CPE elements present in the 3′ UTR causes a further increase in translation rate in GV oocytes, indicating a synergism between the two RBPs. Manipulation of the poly(A) length in these mutants points to a cooperative in maintaining a mRNA deadenylated. Conversely, mutation of the CPE element blocks the activating effects of DAZL mutation during maturation. We propose that DAZL represses translation in a subset of mRNAs by promoting deadenylation in concert with CPEB1 in GV oocytes. The DAZL effects continue during maturation, by counteracting the adenylating and activating effects of CPEB1. Since DAZL and CPE elements coexist on the majority of mRNAs, this interaction between the two proteins may be a mechanism mediating the translational repression by DAZL. It is most likely that Dazl assemble different macromolecular complexes on the 3′UTR of an mRNA in concert with other RBPs, ultimately leading to activation or repression of translation.

On another note, in silico analysis of oocyte mRNAs identifies a subset of transcripts whose translation is affected by DAZL depletion despite a lack of interaction with DAZL. These mRNAs do not contain a DAZL element and are not immunoprecipitated by DAZL antibody in a RIP-Chip paradigm, confirming that a small subset of mRNAs is indirectly affected by DAZL depletion. Since DAZL is involved in regulation of ribonuclear proteins, the translation of several regulators of the translational program may serve as an intermediate for these indirect effects. Although not supported by some of our findings, we cannot formally exclude that Dazl depletion also affects translation by altering the balance between the pools of free and translating ribosomes.

Recently, it was reported that DAZL is dispensable for oocyte maturation, and instead, its overexpression has deleterious effects on oocyte developmental competence^23^. This conclusion is based on the observation that DAZL protein is markedly decreased in the adult ovary in comparison with neonatal ovary; however, the variable ratio of somatic:germ cells in the gonad during development may account, at least in part, for these differences^23^. The DAZL protein expression detected with two distinct antibodies, the RIP-Chip data, and the translational regulations data are consistent with the expression and increased accumulation of DAZL in the final stages of oocyte development. Genetic manipulations led to the conclusion that the absence of DAZL does not produce overt phenotypes on oocyte maturation or fertility. The genetic background used in these experiments is a mixed background (ICR and C57BL/6N) whereas we use a pure C57BL/6 background. It has been noted that the penetrance of the phenotypes associated with *Dazl* gene ablation are sensitive to the mouse background used^14^. Nevertheless, the view that under physiological conditions DAZL needs to be expressed within a very narrow range of concentrations is consistent with our findings that DAZL has a dual effect on translation, functioning as a repressor and activator. Therefore, it is possible that increased DAZL levels favors translational repression that would be detrimental to developmental competence. Aside from the genetic background of the mice used, not immediately evident is the explanation of why DAZL KO in neonatal oocytes produces undetectable phenotype on fertility. Possible off-target effects of DAZL morpholinos are inconsistent with the rescue experiments we have performed. In several cases, it has been observed that morpholino oligonucleotide treatment is associated with induction of *p53*^37,38^ (*tp53* or *trp53*) or interferon response or toll-like receptor^39^. Since transcription is repressed in GV oocytes, it is unlikely that MO off-target effects include changes in transcription. Another possible explanation of the divergent observations is that the oocyte does not tolerate acute depletion of DAZL as in our experimental paradigm, while it has time to adjust to loss of DAZL early during the follicle growth phase that starts in the neonate ovary. Genetic compensation has been shown to be at the basis of differences in phenotypes produced by mutations but not knockdowns^40^. Since we have shown that DAZL functions in partnership with CPEB1^22^, it is possible that this latter RBP would compensate for the loss of DAZL. In this respect, it would be important to determine whether CPEB1 expression is affected in the DAZL KO and how the translational program is executed in the absence of DAZL in the fully grown oocyte.

In summary, our findings are consistent with a role of DAZL in the translation program executed during the final stages of oocyte maturation. The dual function as repressor and activator suggests complex changes in the proteome in fully grown oocytes and during maturation are dependent on DAZL action. These findings imply that spontaneous DAZL mutations found in humans may affect not only germ cell development in the fetal gonad but oocyte quality as well. Such a possibility has been proposed with the description of DAZL missense mutations in infertile women^41^.

## Methods

### Experimental animals

All experimental procedures involving the animal models used were approved by the Institutional Animal Care and Use Committee of the University of California at San Francisco (protocol AN101432). Mice was housed in 12 h light/12 h dark cycle in temperature of 18–23 °C with 40-60% humidity environment. Pure C57BL/6 female mice (21–24-days-old) carrying the DAZL TM1^Hgu^ allele (ΔDAZL) were generated as previously described^21,22^. Rpl22tm1.1Psam/J (RiboTag) mice, with a targeted mutation that provides conditional expression of the ribosomal protein L22 tagged with three copies of the HA epitope. Rpl22tm1.1Psam/J homozygous males were crossed with C57BL/6-TgN (Zp3-Cre) 82Knw (Jackson Laboratories) females to produce C57BL/6-Zp3-Cre-Rpl22tm1. 1Psam (*Zp3-Cre-RiboTag*) mice. For breeding *RiboTag*^*fl/fl*^*;Zp3-Cre;Dazl*^*+/+*^ or *RiboTag*^*fl/fl*^*;Zp3-Cre;Dazl*^*+/−*^, C57BL/6-*RiboTag*^*fl/fl*^*;Zp3-Cre;Dazl*^*+/+*^ or *RiboTag*^*fl/fl*^*;Zp3-Cre;Dazl*^*+/−*^ males were crossed with C57BL/6-*RiboTag*^*fl/fl*^*;Zp3-Cre;Dazl*^*+/+*^ or *Dazl*^*+/−*^ females to obtain C57BL/6-ΔDAZL-*RiboTag*^*fl/fl*^*;Zp3-Cre* mice.

### Oocyte collection and microinjection

Oocyte isolation and microinjection were performed using HEPES modified minimum essential medium Eagle (Sigma-Aldrich, M2645) supplemented with 0.23 mM pyruvate, 75 µg/mL penicillin, 10 µg/mL streptomycin sulfate, and 3 mg/mL BSA, and buffered with 26 mM sodium bicarbonate. To prevent meiosis resumption, 2 µM cilostamide (Calbiochem, 231085) was added in the isolation medium. Oocyte in vitro maturation was performed using Eagle’s minimum essential medium with Earle’s salts (Gibco, 12561-056) supplemented with 0.23 mM sodium pyruvate, 1% streptomycin sulfate and penicillin, and 3 mg/mL bovine serum albumin (BSA). For microinjection, cumulus cells were removed by mouth pipette from isolated cumulus oocyte complexes (COCs), and denuded oocyte were injected with 5–10 pL of 12.5 ng/µL mRNA reporter using a FemtoJet Express programmable microinjector with an Automated Inverted Microscope System (Leica, DM4000B). After washing and pre-incubating overnight in α-MEM medium supplemented with 2 µM cilostamide, oocytes were released in inhibitor-free medium for in vitro maturation at 37 °C under 5% CO_2_.

### Oocyte morpholino antisense oligonucleotide microinjection

Germinal vesicle (GV) stage oocytes were isolated from *RiboTag*^*fl/fl*^*;Zp3-cre;Dazl*^*+/+*^ or *RiboTag*^*fl/fl*^*;Zp3-cre;Dazl*^*+/−*^ mice. After pre-incubated in α-MEM medium supplemented with 2 μM cilostamide for 1 h at 37 °C under 5% CO2, 5–10 pl of 1 mM morpholino oligonucleotides (Gene Tools) of standard control (5′-CCTCTTACCTCAGTTACAATTTATA-3′) or against DAZL (5′-CCTCAGAAGTTGTGGCAGACATGAT-3′) were injected into *RiboTag*^*fl/fl*^*;Zp3-cre;Dazl*^*+/+*^ or *RiboTag*^*fl/fl*^*;Zp3-cre;Dazl*^*+/−*^ mice using a FemtoJet express microinjector. Following overnight incubation in α-MEM containing 2 μM cilostamide medium, oocytes were released in α-MEM medium without inhibitor for in vitro maturation or recording under the microscope.

### RiboTag IP RNA-Seq

Oocytes from *RiboTag*^*fl/fl*^*;Zp3-cre;Dazl*^*+/+*^ or *RiboTag*^*fl/fl*^*;Zp3-cre;Dazl*^*+/−*^ mice were collected as described above. *RiboTag*^*fl/fl*^*;Zp3-cre;Dazl*^*+/+*^ oocytes were injected with a CON-MO, while the *RiboTag*^*fl/fl*^*;Zp3-cre;Dazl*^*+/−*^ oocytes were injected with a DAZL-MO. Control experiments show that *Dazl*^+/*−*^ oocytes have maturation timing and PB extrusion rates identical to wild type oocytes but a 50% decrease in *Dazl* mRNA and protein. In addition, pilot experiments showed a dosage effect in DAZL depletion and MII stage block when comparing DAZL-MO injected in *RiboTag*^*fl/fl*^*:Zp3-cre: Dazl*^*+/+*^ oocytes versus DAZL-MO injected in *RiboTag*^*fl/fl*^*:Zp3-cre:Dazl* ±  oocyte.

Oocytes injected with CON-MO and DAZL-MO were preincubated overnight in the presence of 2 μM milrinone and the following morning transferred to maturation medium and incubated for 6 h. At the end of the incubation, only oocytes that had undergone GVBD were collected in 5 µl 0.1% polyvinylpyrrolidone (PVP) in PBS, flash frozen in liquid nitrogen, and stored at −80 °C. In parallel, GV oocytes were kept in milrinone, then harvested and processed together with the MI oocytes. A total of 2000 oocytes (0 and 6 h with either CON-MO or DAZL-MO injection) were injected and cultured for the duplicate determination of the effect of DAZL depletion on ribosome loading of endogenous mRNAs. On the day when the RiboTag IP was performed, oocytes were thawed, lysed and an aliquot of the oocyte extract was saved and stored to measure total transcript levels before the IP (input). RiboTag IP was performed as described in the section on Immunoprecipitation. After IP, all samples were used for RNA extraction using the RNeasy Plus Micro kit (Qiagen, 74034). The quality of the extracted RNA was monitored with Bioanalyzer chips (Agilent). RNA samples were transferred to the Gladstone Institutes Genomics Core for cDNA library preparation using the Ovarion RNA-Seq System V2 (NuGen). Samples were sequenced using the HiSeq400 platform.

### Real-time qPCR

Real-time qPCR was performed using Power SYBR PCR master mix with ABI 7900 Real-Time PCR system (Applied Biosystems). All oligonucleotide primers used in this project were designed against two exons flanking an intron to avoid amplification of genomic DNA (Supplementary Table 1). The specificity of each pair of primers was verified by using a unique dissociation curve, performed at the end of the amplification. Data was normalized to its corresponding input and IgG in RiboTag IP/qPCR for HA and DAZL antibody and expressed as the fold-enrichment of 2^−ΔΔCt^.

### Western Blots

Oocytes were lysed in 10 µl 2x Lammli buffer (Bio-Rad) supplemented with mercaptoethanol and a cocktail of phosphatase and protease inhibitors (Roche). The oocyte lysates were boiled for 5 mins at 95 °C and then transferred to an ice slurry, then separated on 10% polyacrylamide gels and transferred to a polyvinylidene difluoride (PVDF) membrane. Membranes were blocked in 5% milk for 1 h at room temperature and incubated with primary DAZL antibody (ab215718, Abcam, 1:1000) or V5 antibody (R960-25, Invitrogen overnight at 4 °C. An antibody against α-tubulin (T6074, Sigma-Aldrich; 1:1000) or Actin (ab14128, Abcam), was used as a loading control. After overnight incubation, membranes were washed in TBS-Tween 20 (0.05%) three times and incubated with HRP-conjugated secondary antibodies (anti rabbit IgG: LNA934V/AH, lot9761196, anti-mouse IgG: LNA934V/AH, lot 9739640 Sigma-Aldrich) (1:5000) for 1 h at room temperature. The signals were detected using Super Signal West Fremto (Thermo Scientific, 34095). Bands intensity was evaluated with ImageJ. The quantification of each immunoreactive signal was obtained by measuring pixel density, by subtracting the background intensity, followed by correcting each pixel density for the intensity of the loading controls.

### Immunoprecipitation

RiboTag IP or DAZL RIP analysis was performed on the basis of publications and our own modification for applicable to mouse oocytes measurement^21,22^. Briefly, GV-arrested or MI oocytes (200 oocytes/sample) were washed and collected in RNase-free PBS with 1% polyvinylpyrrolidone. After lysis in 300 µl of supplemented homogenization buffer S-HB; 50 mM Tris-HCl pH 7.4, 100 mM KCl, 12 mM MgCl_2_, 1% NP-40, 1 mM dithiothreitol, protease inhibitors, 40|U RNAseOUT, 100 μg/ml cycloheximide and 1 mg/ml heparin (Sigma-Aldrich, H3393)], samples were centrifuged at 12,000 g for 10 mins and supernatants were precleared with prewashed Protein G magnetic Dynabeads (Invitrogen, 10007D) for 30 mins at 4 °C. In all, 15 µl of precleared lysates was aliquoted for input (total transcripts) and stored at −80 °C for mRNA extraction the next day. The remaining precleared lysates were incubated with specific antibody (anti-HA antibody, anti-DAZL antibody) or its corresponding IgG (mouse IgG, Cat: 12-371, Lot: 2757162, Sigma-Aldrich); rabbit IgG, ab37415; Abcam) 4 h at 4 °C on a rotor. Then pre-washed Protein G magnetic Dynabeads were added to the lysates for overnight incubation at 4 °C on a rotator. The following day, bead pellets were washed three times in 500 µl homogenization buffer (HB) on a rotor at 4 °C for 10 mins. Two more washes were performed with 1 M urea/high-salt buffer for 10 min each. RNA eluted from the beads was either HA-tagged ribosome associated transcripts or IgG (no-specific binding transcripts), together with input for extraction. In some experiments, the specificity of the immunoprecipitation was determined by using WT rather than RiboTag mice. RNA was purified with RNeasy Plus Micro kit (Qiagen, 74034) according to manufacturer’s instructions and directly used for RNA-Seq analysis or reverse transcription for qPCR analysis. cDNA was prepared using SuperScript III First-Strand Synthesis system (Invitrogen, 18080-051) with random hexamer oligonucleotide primers. cDNA samples were stored at −80 °C for following experiments.

For the RiboTag/qPCR analysis, *CcnB1*, *Dppa3,* and *Gdf9* (transcripts not regulated by DAZL as previously reported^21,22^), were used to normalize the qPCR data. Zp3 contains no recognizable DAZL-binding element and was used as a negative control for DAZL immunoprecipitation. The data are reported as fold enrichment, with IgG values set to 1.

### Reporter mRNA preparation and reporter assay

The *Oosp1*, *Obox5*, *CenpE,* and *CcnB1* 3′UTR sequences were obtained by amplification of an oocyte cDNA library. After sequencing, the oocyte cDNA was cloned downstream of the YPet coding sequence. An oligo (A) stretch of 20 A was added in each construct. All constructs were prepared in the pcDNA 3.1 vector containing a T7 promoter, allowing for in vitro transcription to synthesize mRNAs, and fidelity was confirmed by DNA sequencing. mRNA reporters were transcribed in vitro to synthesize mRNAs with mMESSAGE mMACHINE T7 Transcription Kit (Ambion, AM1344); polyadenylation of *mCherry* and some of the YFP reporters in Fig. 9e as reported in the figure legend were obtained using Poly(A) Tailing Kit (Ambion, AM1350). All messages were purified using MEGAclear Kit (Ambion, AM1908). mRNA concentrations were measured by NanoDrop, and its integrity was evaluated by electrophoresis.

Time-lapse recordings were performed using a Nikon Eclipse T2000-E equipped with a mobile stage and environmental chamber of 37 °C and 5% CO_2_. *YFP-Oosp1*, *YFP-Obox5*, *YFP-Cenpe* or *YFP-CcnB1* were injected at 12.5 ng/µL with either CON-MO or DAZL-MO. Each *YFP-3*′*UTR* reporter was also co-injected with polyadenylated *mCherry* at 12.5 µg/µL in oocyte. After injection, oocytes were pre-incubated overnight in α-MEM medium supplemented with 2 µM cilostamide to allow expression of the reporters. mCherry signals did not change significantly in oocytes at different stages of maturation. Ratios of YFP reporter to the level of mCherry signal measured at plateau in each oocyte were calculated. In those cases where DAZL ablation had an effect in GV oocytes, the data were normalized to the signal of GV stage accumulation of corresponding proteins. Rates of translation associated with reentering into cell cycle (after GVBD versus before GVBD) were calculated by fitting YFP:mCherry data and calculating the slope of the interpolation obtained by linear regression (Prism) prior to GVBD or after GVBD when a new rate of translation had stabilized.

For the experiments where translation of oligo-adenylated and poly-adenylated reporters is compared, the following calculations were performed. The rates of translation of reporters were measured by fitting (linear regression) the yfp/mCherry values during the first 0–3 h or at 7–10 h of incubation. If accumulation of a reporter follows a linear pattern, rates measured by fitting the data at the beginning and the end of incubation are not significantly different, indicating that translation proceeds at a constant rate throughout the experiment, a conclusion verified by goodness of fit (R) of a linear regression through the entire recording interval (0–10 h). If, however, the accumulation of the reporter is not linear, a change is rates is observed. The significance of the changes is assessed by a *t*-test with Welch correction without assuming normal distribution of the measurements and by calculating the ratio of the rates for the oligo and poly adenylate reporter. We have shown that the ratios between the two measurements provide an estimate of the rate of adenylation or deadenylation of the reporter^13^. An increase in translation of the oligoadenylated reporter during the time course and no changes in translation of the polyadenylate reporter indicate an increase in polyadenylation by endogenous adenylases. Repression of translation shows no major changes in the oligo-adenylated reporter translation and these changes are amplified in the polyadenylated reporter.

### DAZL RIP-Chip analysis

DAZL RIP-Chip was performed as previously reported^21^. Briefly, C57BL/6 female mice (22–24-days-old) were primed with PMSG, and after 48 h mice were stimulated with hCG for 0, 6, or 14 h to collect GV, MI, and MII stage oocytes. Oocyte lysates were centrifuged at 12,000 g for 10 mins at 4 °C. Supernatants were used for RNA extraction. RNA was purified with RNeasy Plus Micro kit (Qiagen). RNAs in the RNP fractions were reverse-transcribed with SuperScript III (Invitrogen). Five micrograms of cDNA was fragmented and hybridized with Affymetrix Mouse Genome 430.2 array chips^42^. DNA-Chip Analyzer (dChip) was used for normalization and to quantify microarray signals with default analysis parameters. Comparison between samples was performed using dChip with a fold change of 1.5, FDR < 5%, and *P* < 0.05.

### Statistical analysis

Statistical analysis was performed using the GraphPad Prism 8 package. The statistical analysis performed depended on specific experiments and is reported in the figure legend. For comparison between the two groups, two-tailed unpaired t-test was used. The data collection software Metamorph 7.8.13.0, Image J bundled with 64-bit Java 1.8.0_112, were used for data collection and analysis. The method used for statistical analyses is reported in each figure legend. RNA-Seq cDNA library preparation using the Ovation RNA-Seq System V2 (NuGen, 0344). Samples were sequenced using the HiSeq4000 platform. The quality check of RNA-Seq reads was performed using FastQC and reads were then trimmed with Trimmomatic-0.33. Alignment of the reads to the mouse genome was performed by STAR 2.4.2a,.bam files were sorted and indexed using Samtools1.10, and count files were generated by HTSeq (0.9.1galaxy1). RNA-Seq statistical analyses were done through DESeq2 (Version 1.18) and other Bioconductor scripts.

### Reporting summary

Further information on research design is available in the Nature Research Reporting Summary linked to this article.

## Supplementary information

Supplementary Information

Description of Additional Supplementary Files

Supplementary Data 1

Supplementary Data 2

Reporting Summary

## Data Availability

All data generated or analyzed during this are included in this published article in its Supplementary Information. Raw data have been deposited in the Sequence Read Archive under the accession number PRJNA603993. Source data for Figs. 1–9 and Supplementary Figs. 1–13, 15 are provided.

## Source Data

Source Data
