# Supplementary material for: The RNA-binding protein DAZL functions as repressor and activator of mRNA translation during oocyte maturation
Source: Nat Commun. 2020 Mar 13;11:1399. doi: 10.1038/s41467-020-15209-9 (PMC7070028; doi:10.1038/s41467-020-15209-9)
Supplement: Supplementary file 1 — Supplementary Information [file 41467_2020_15209_MOESM1_ESM.pdf]

SUPPLEMENTARY INFORMATION

The RNA binding protein DAZL functions as repressor and activator of maternal mRNA translation during oocyte maturation

Cai-Rong Yang et al.

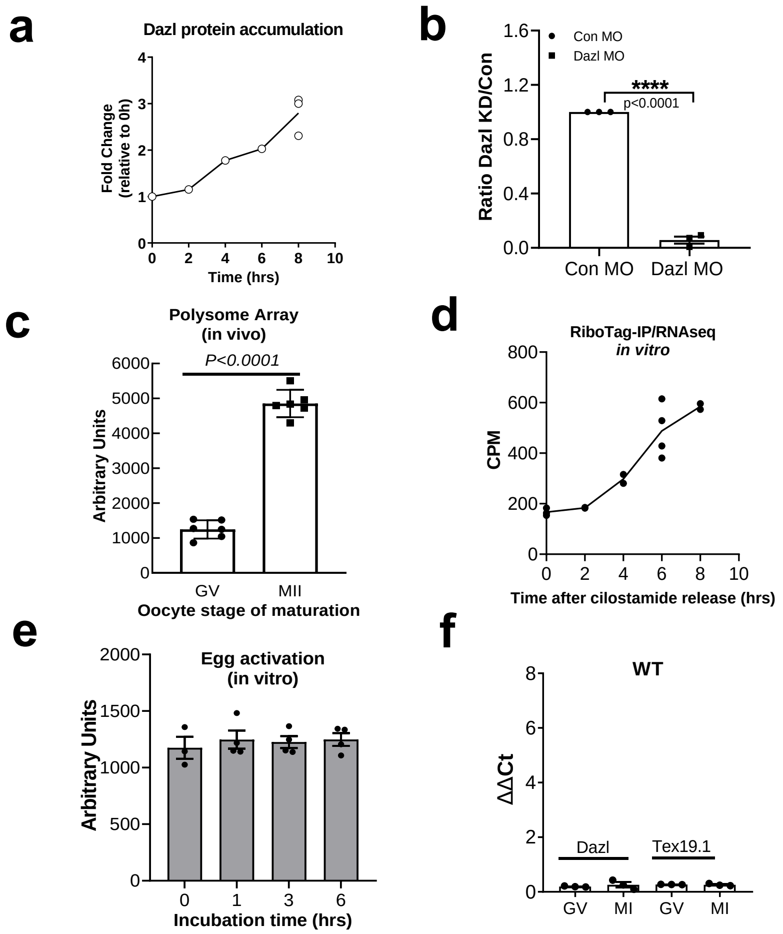

Supplementary Figure 1. Dazl mRNA translation is regulated during oocyte maturation.

(a) Quantification of Western Blot monitoring DAZL protein accumulation during oocyte maturation to MI. **(b)** Quantification of the immunoreactive DAZL expression in oocytes after CON-MO and DAZL-MO injection. Each bar is the Mean ± SEM of 3 biological replicates. Data was analyzed by unpaired two-tailed t-test, ($p < 0.0001$) **(c)** Dazl mRNA becomes associated with the polysome fraction at the GV-to-MII transition *in vivo*. Polysome array data described in our previous report[1] was mined for the Dazl mRNA association with polysomes. The data were analyzed by unpaired two-tailed t-test, are the Mean ± SEM of 6 independent biological replicates ($p < 0.0001$). **(d)** Ribosome loading on the *Dazl* mRNA increases during *in vitro* oocyte maturation. The details of the experiments are summarized in our recent published paper[2]. n=2 biologically independent experiments except for the 6 hrs point (n=4). **(e)** Polysome associated transcript upon egg activation *in vitro*. MII oocytes were treated *in vitro with* Sr2+ for the indicated times. At the end of the incubation, cells were harvested and processed for polysome arrays. Each bar is the Mean ± SEM of n=3 or 4 biological independent replicates and values are reported as dots. **(f)** RiboTag IP/qPCR of oocyte extracts from wild type mice. n=3 biologically independent experiments. The mRNA recovered is at background levels and is similar in all the experimental groups.

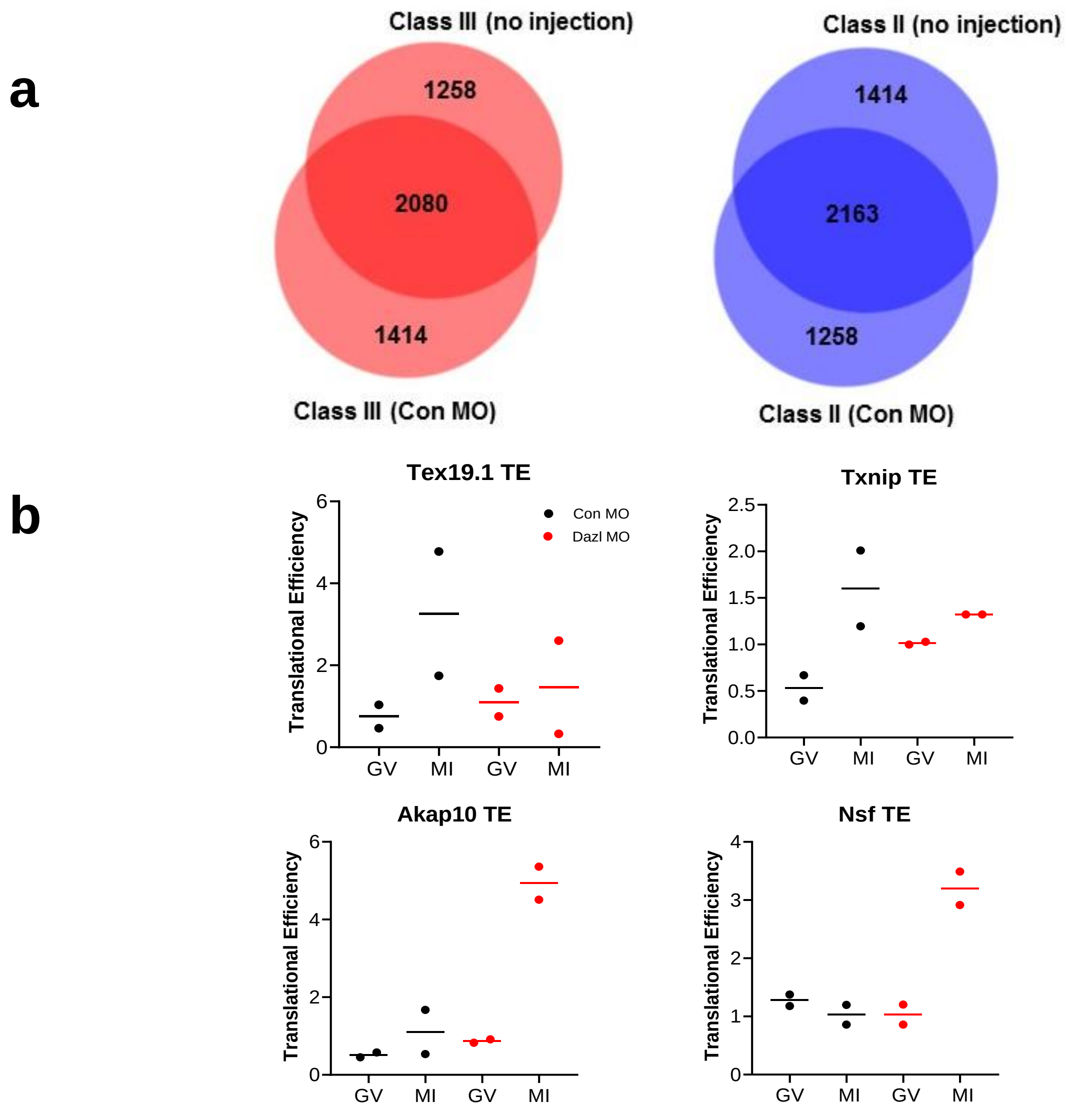

Supplementary Figure 2. Transcript translation is comparable in oocytes with or without CON-MO injection.

(a) Comparison of the RiboTag IP/RNA-Seq data from CON-MO injected oocytes with RiboTag IP/RNA-Seq data using non-injected oocytes. The RNA-Seq data from non-injected oocytes are from Ref 32. Increased and decreased ribosome loading onto mRNAs at 6 hrs is compared. Class III are the group of transcripts recruited to the polysome during maturation, implying an increase in translation. Class II are the group of transcripts released from the polysome imply translational repression. Quantitatively the data could not be directly compared because of batch effects. **(b)** Calculation of the translational efficiency (TE) for the candidate mRNAs reported in Figure 2C. Data are as mean values of n = 2 biologically independent experiments. TE is calculated as the ribosome-bound CPM divided by the total mRNA levels. CPM = Counts per million.

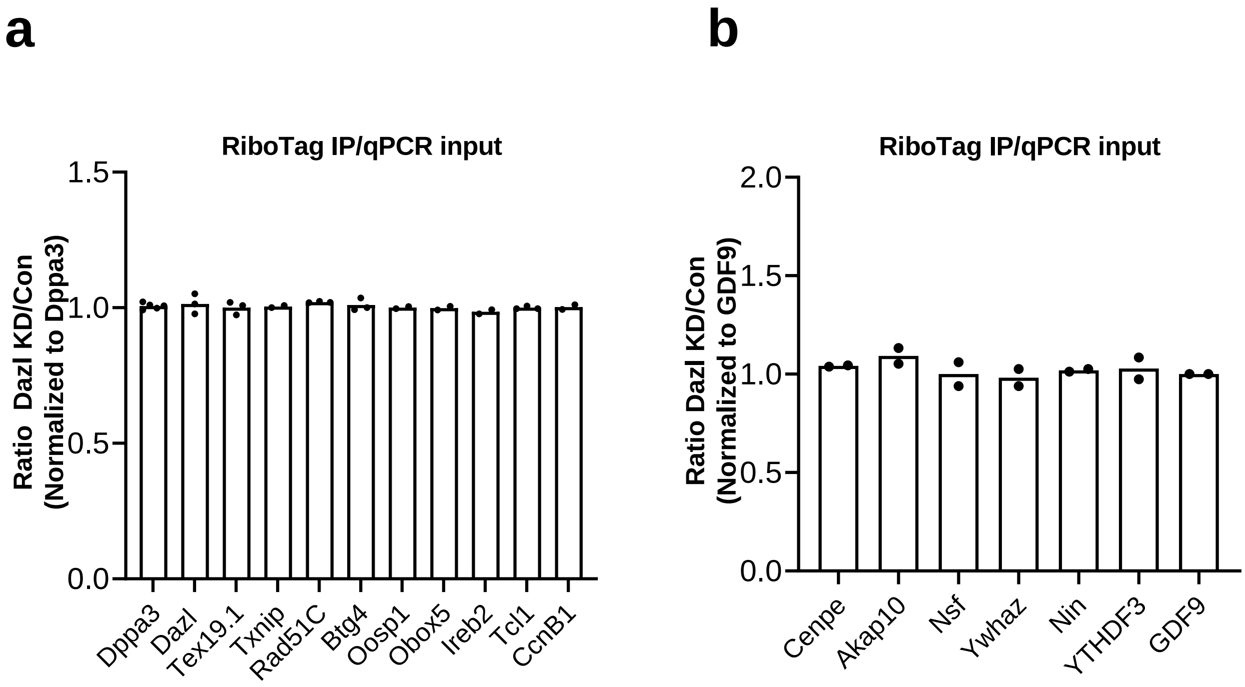

Supplementary Figure 3. Total transcript levels for selected transcripts is not affected by Dazl knockdown.

Total mRNA levels of representative Dazl targets reported in Figure 3 is included. GV stage oocytes from *RiboTag$^{fl/fl}$:Zp3-CRE* or *RiboTag$^{fl/fl}$:Zp3-CRE*:*Dazl$^{+/-}$* mice were injected with CON-MO or DAZL-MO. Oocytes were collected at 6 hrs of *in vitro* culture for RIP-qPCR analysis of total transcript levels prior to RiboTag immunoprecipitation (input). The ratio of Dazl-MO group and Con-MO group is reported. Data are presented as mean values ± SEM n=3 **(a)** or n=2 **(b)** biologically independent experiments.

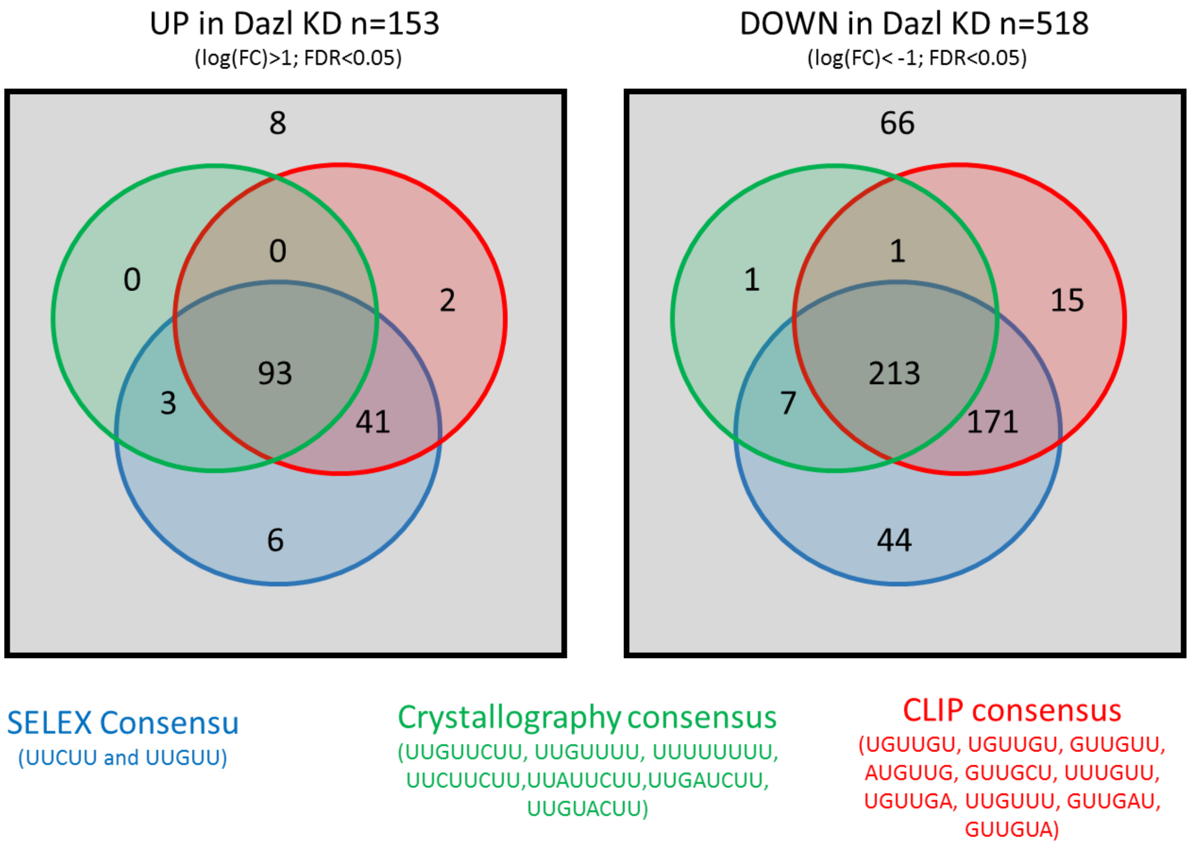

Supplementary Figure 4. A subset of mRNAs affected by Dazl depletion does not contain discernible Dazl elements.

The RiboTag IP/RNA-Seq analysis (Figure 2b) identified 170 transcripts upregulated (FDR<0.05 $Log_2FC \geq 1$) and 551 transcripts downregulated (FDR<0.05, $Log_2FC \leq -1$) in oocytes depleted of Dazl. The 3' UTR of 153 UP transcripts out of 170 and 518 DOWN transcripts out of 551 were retrieved from the UCSC database. The 3' UTRs of these transcripts affected by the Dazl depletion were scanned for the presence of Dazl consensuses as established using different experimental approaches (Selex in blue, Crystallography in green, and CLIP in red). Transcripts with overlapping and non-overlapping consensuses are represented as Venn diagrams color-coded for the consensus, with the number of transcripts identified for each group included inside the circle. 93 UP and 213 Down 3'UTRs are positive for putative Dazl binding elements according to all three sets of consensuses. Using this analysis, 8 UP and 66 DOWN transcripts do not contain any discernible Dazl element and are reported outside the overlapping circles.

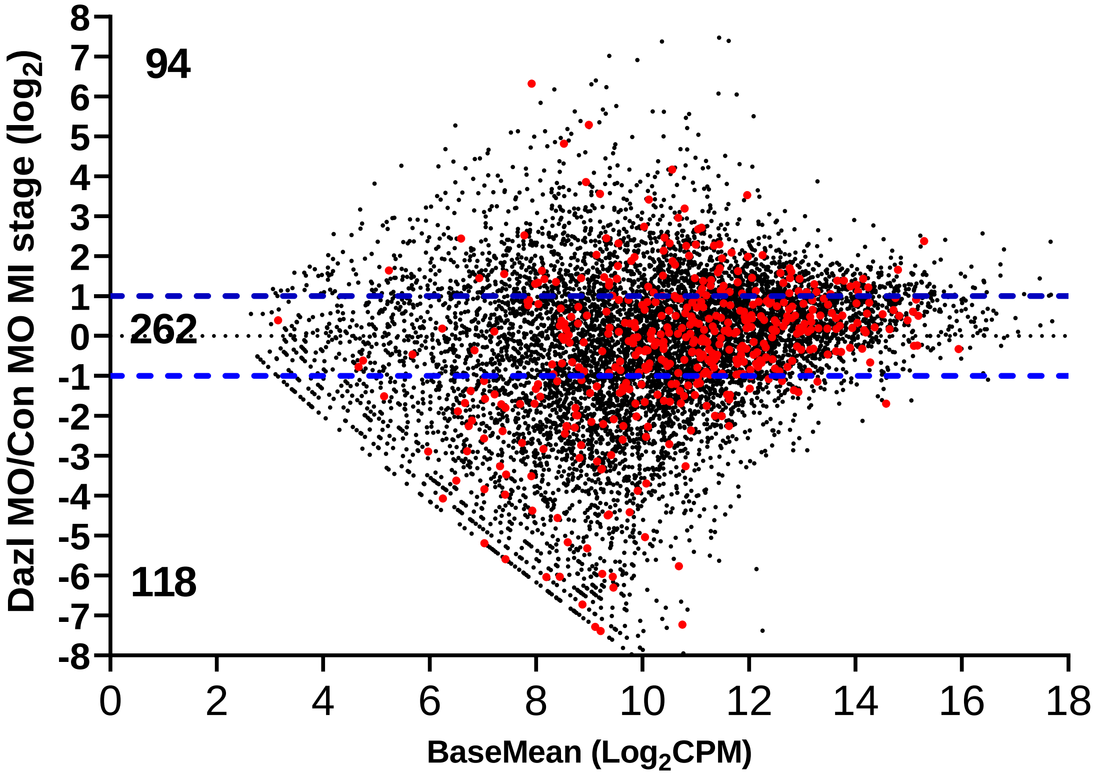

Supplementary Figure 5. Comparison between genes whose ribosome loading is affected by Dazl depletion and their interaction with Dazl in Rip-Chip.

When using a RIP-Chip assay, 847 transcripts are significantly immunoprecipitated ($p<0.05$, FC >1.5) by the Dazl antibody. When comparing this dataset to the RNA-Seq data, only 474 are present in both datasets. This subset of data is plotted together with the RiboTag IP/RNA-Seq log2 fold change ratio between the Dazl-MO and Con-MO versus the base mean. The genes marked in red are specifically immunoprecipitated by a Dazl antibody in the Dazl-RIP-CHIP experiment (FC>1.5, $P<0.05$). The dashed blue lines mark the 2 fold change threshold in the RNA-Seq dataset. CPM: counts per million of reads. The presence of transcripts that bind Dazl but that are not significantly affected by Dazl depletion is consistent with data reported during depletion of Dazl in spermatogenesis[3].

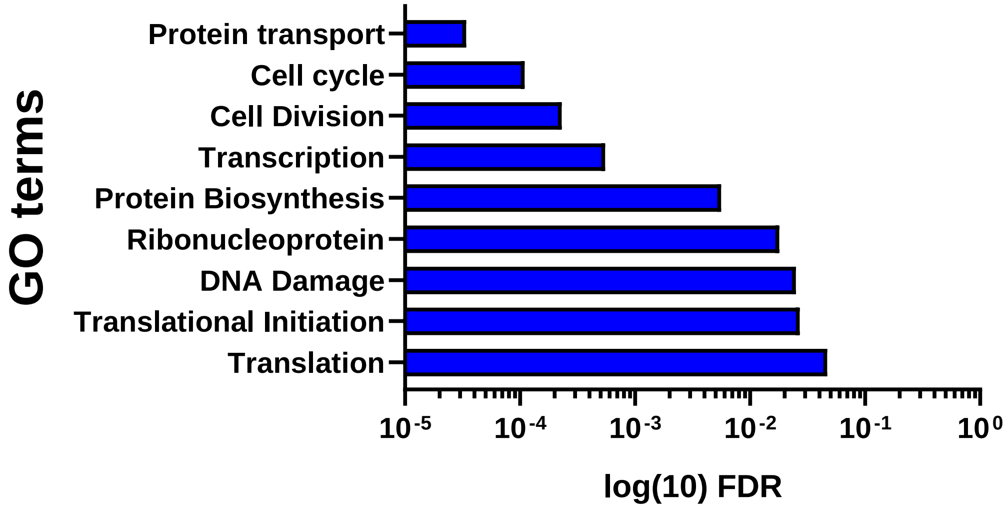

Supplementary Figure 6. Gene ontology analysis of transcripts whose translation is affected by Dazl depletion in the oocytes.

The transcripts whose translation is affected by Dazl depletion (FDR <0.05, >2 fold change) were subjected to analysis with the David GO Tool for Functional Annotation Clustering. Terms significantly enriched (FDR< 0.05) are reported in the graph.

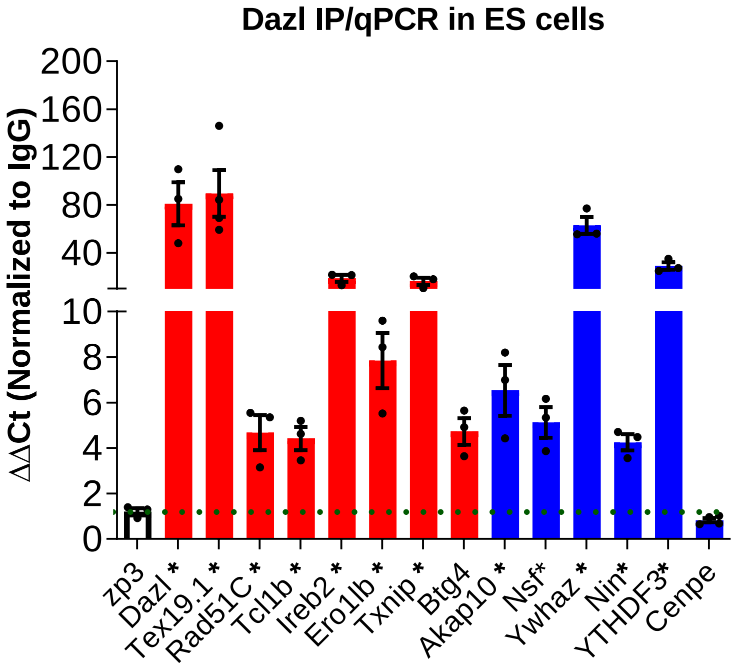

Supplementary Figure 7. DAZL RNA-IP/qPCR of Mouse embryonic stem (ES) cell extracts.

ES cells were cultured in DMEM medium with supplements including 15% KOSR, 2% FBS, 1x MEM Non-Essential Amino Acids (100x), 1x 2-mercaptoethanol, 10^{-7} U/ml LIF, 1x Pen/Strep and 2i (1 µM PD0325901 and 3µM CHIR99021) and collected for DAZL RNA-IP/qPCR analysis. The results are normalized with IgG IP. Red bar represents the transcripts whose translation is downregulated by DAZL removal in the oocyte, while blue bars represent the transcripts whose translation is downregulated. Data are presented as mean ± SEM of 3 biologically independent experiments. Asterisks (*) indicate that these transcripts are also found to be associated with DAZL in the RIP-Chip dataset. It should be noted that ES cells maintained in the ground state express DAZL, as well as a large number of two-cell embryo transcripts, mRNAs often expressed also in oocytes[3-5].

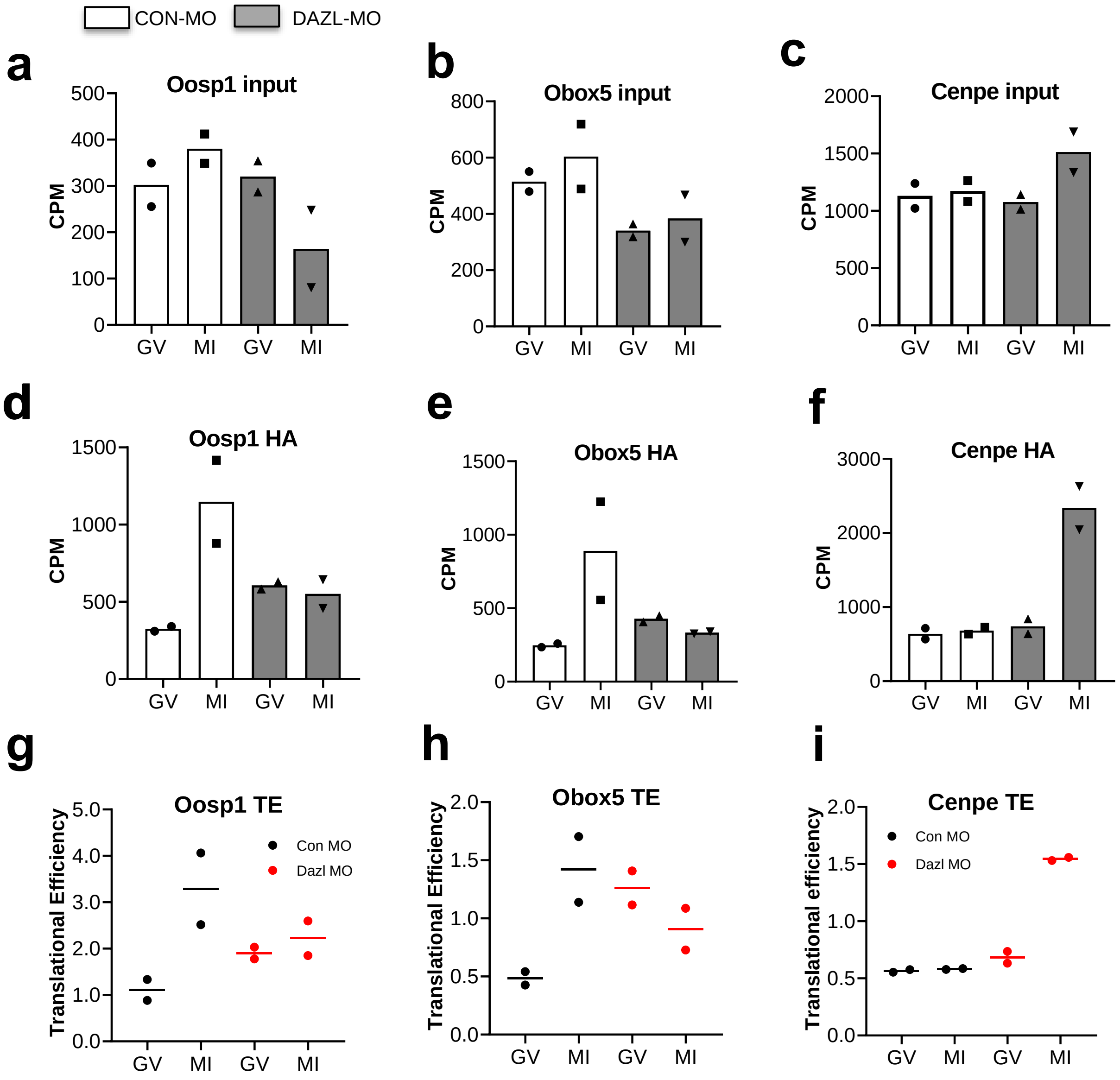

Supplementary Figure 8. RiboTag IP/RNA-Seq data and translational efficiency for *Oosp1*, *Obox5* and *Cenpe* transcripts.

Panels (**a**), (**b**), and **(c)** report the input levels of mRNAs for the three transcripts measured by RNA-Seq of an aliquot of oocyte extract prior to RiboTag IP. Panels **(d)**, **(e)**, and **(f)** report the RNA-Seq data for the mRNAs recovered in the RIboTag IP pellets. Empty bars are the mean from the CON-MO group while the shaded bars are from the DAZL-MO group. Panels **(g)**, **(h)**, and **(i)** report the Translation Efficiency (TE). Data are presented as mean of n=2 biologically independent experiments reported as dots. TE is calculated as described in the Methods. CPM= counts per million of reads.

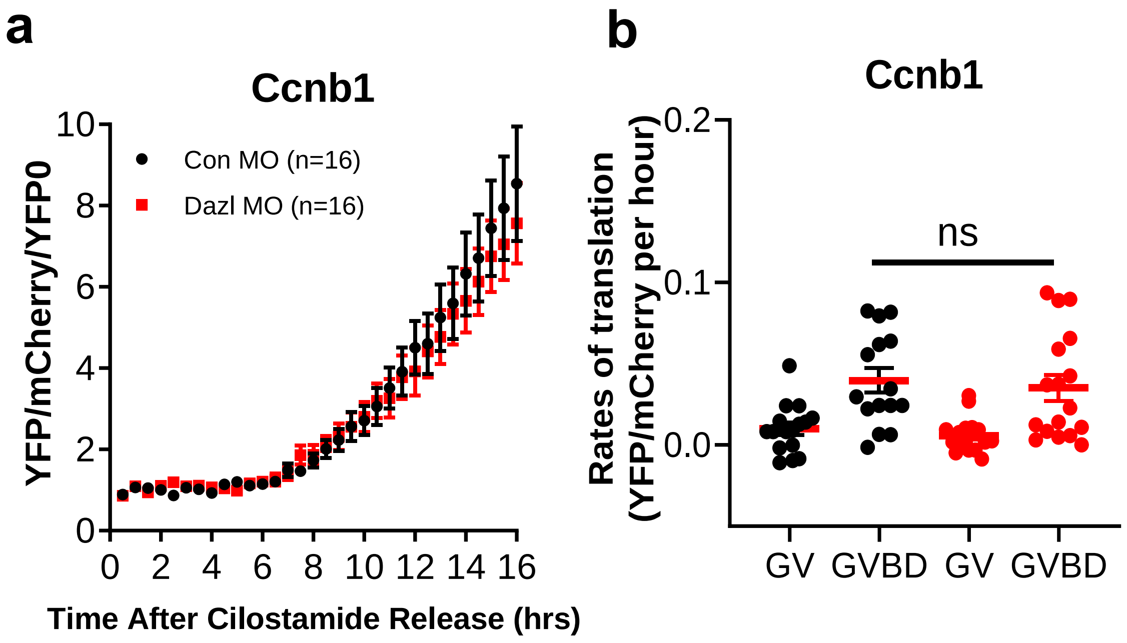

Supplementary Figure 9. Accumulation of the CcnB1 reporter during oocyte maturation.

(a) Oocytes were co-injected with *mCherry*-polyadenylated mRNA and *YFP- Ccnb1 3'UTR-* oligo-adenylated mRNA reporter together with either CON-MO or DAZL-MO. Oocytes were then pre-incubated overnight to allow the mCherry signal to reach a plateau. At the end of this preincubation, oocytes were released in cilostamide-free medium for maturation. The YFP and mCherry signal were recorded by time lapse microscopy as described in the Methods. The YFP signal for each oocyte was corrected by the level of mCherry signal and normalized to the first recording. The black dots correspond to Con-MO injection, while the red dots correspond to Dazl-MO injection group. Each time point is presented as the mean ± SEM of all oocytes tested (n=16). The number of oocytes included in each group is reported among brackets. Rates of translation in GV and GVBD for each oocyte were calculated as described in the Methods and are reported in **(b).** The rate for each individual oocytes from n=3 independent biologically experiments for panel (b) Data were analyzed by unpaired two tailed t-test (p = 0.6738).

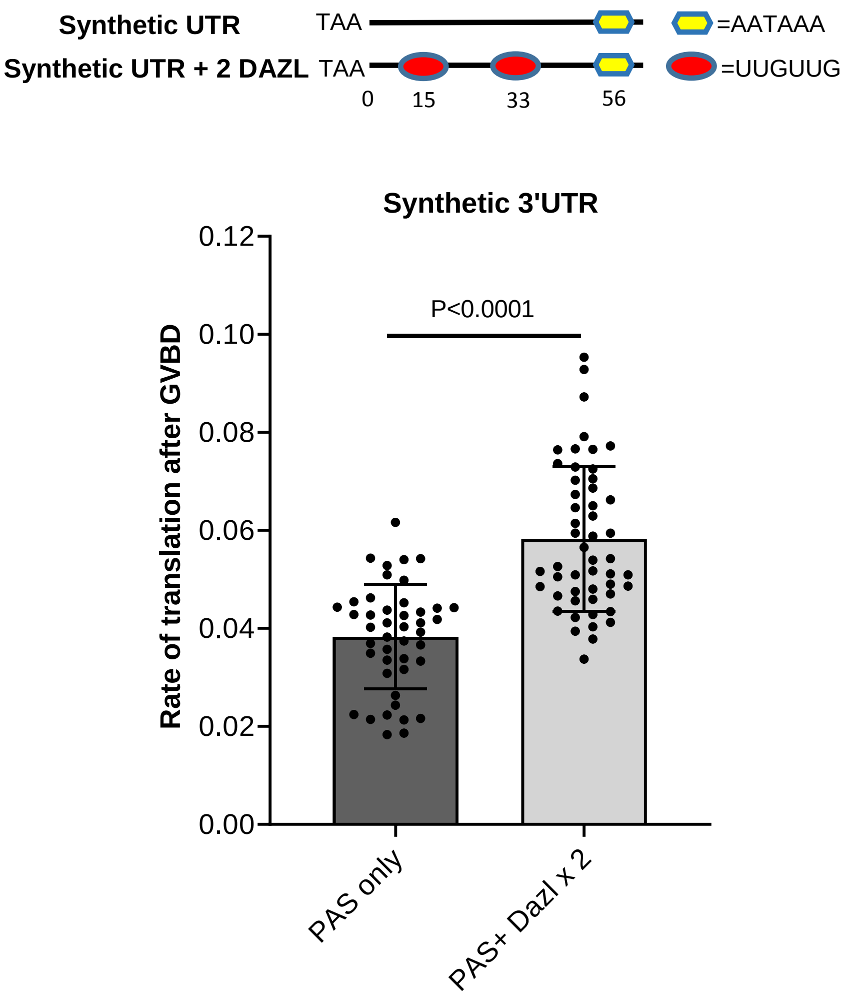

Supplementary Figure 10. Inclusion of Dazl consensus sequences in a synthetic 3' UTR causes an increase in translation of the reporter after GVBD.

A synthetic 3'UTR of 77 nucleotides that contains a PAS element was synthesized with or without a tandem of Dazl element (2x) upstream of the PAS (see scheme). Reporters were constructed by fusing these synthetic 3' UTR to the YFP coding region. After injection in oocytes, rates of reporter accumulation were calculated at the MI stage of maturation, data are presented as the mean ± SEM. Data are from n=3 biologically independent experiments. Each symbol corresponds to rate measurements in a single oocyte (PAS, n=45 oocytes; PAS + Dazl (2x), n=50 oocytes. Data was analyzed by unpaired two tailed t-test, ($p < 0.0001$).

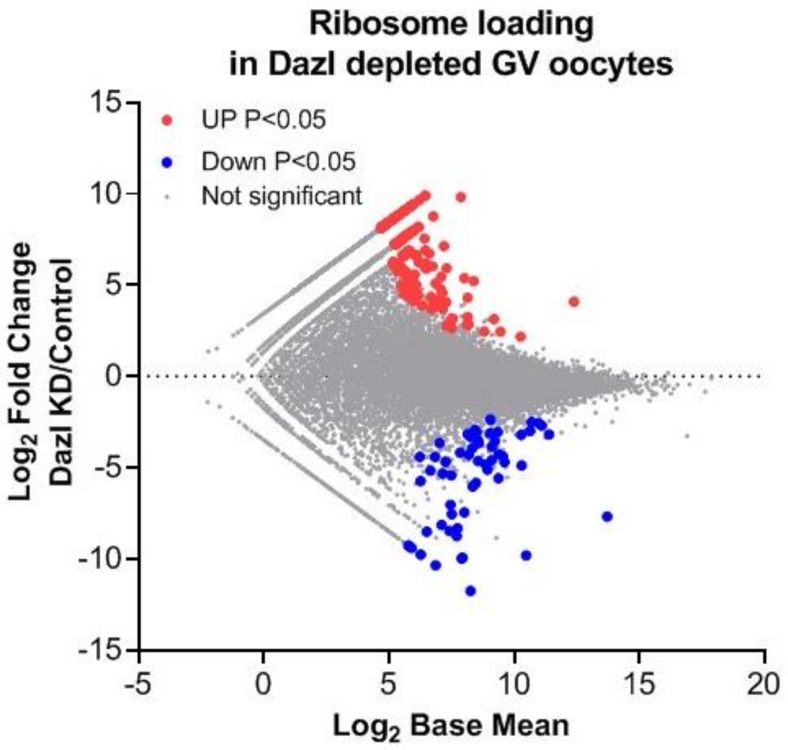

Supplementary Figure 11. Effect of DAZL depletion on ribosome loading of mRNAs in GV-arrested oocytes.

RiboTag IP/RNA-Seq data comparing Dazl-MO versus Con-MO injection in GV stage oocytes are reported. The ribosome loading of 153 transcripts was significantly increased in GV oocytes depleted of DAZL whereas 69 transcripts were decreased (no fold change cutoff, FDR < 0.05).

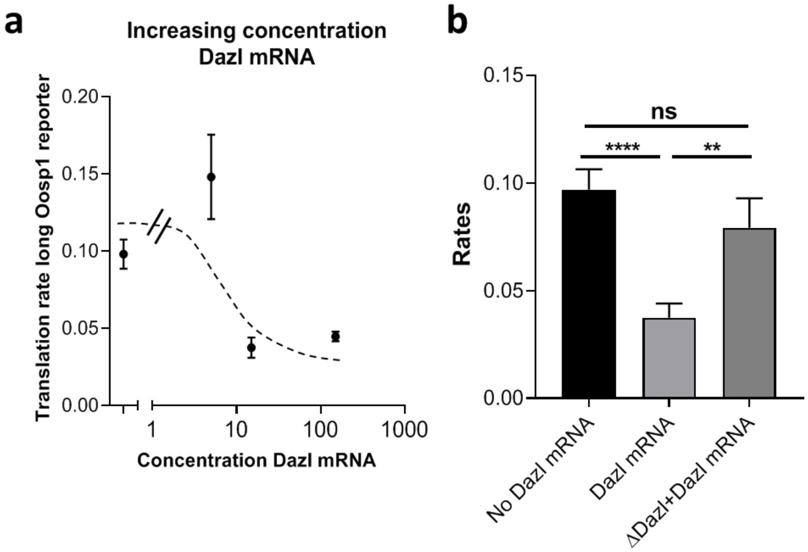

Supplementary Figure 12. Effect of Dazl overexpression on the translation of the Oosp1 long reporter.

(a) Translation of the Oosp1 reporter after coinjection with increasing concentration of mRNA coding for Dazl. Oocytes were injected with increasing concentrations **(**0**,** 1.5, 15 and 150 ng/µl) of V5-tagged Dazl mRNA together with *Oosp1-Long* YFP reporter. After overnight incubation, the rates of accumulation were measured and reported as Mean ± SEM (n=3 biologically independent experiments including samples of 30-90 oocytes). **(b)** Injection of Dazl mRNA rescue the effect of Dazl-binding element mutation on *Oosp1* long reporter in GV stage oocytes. Oocytes were injected with the Oosp1 reporter without or 15ng/µl V5 tagged Dazl mRNA, or with an *Oosp1*-Long reporter with a mutation in the Dazl element. Oocytes were pre-incubated overnight to allow mCherry signal to reach a plateau, then released in cilostamide-free medium. Rates of Oosp1-Long reporter accumulation were calculated in the in the first two hours of incubation when oocytes were still in the GV stage and plotted as Mean ± SEM of three separate experiments. Data was analyzed by unpaired two tailed t-test, panel b: No Dazl mRNA group versus Dazl mRNA group $p < 0.0001$; ΔDazl + Dazl mRNA group versus Dazl mRNA group: p=0.0039; No Dazl mRNA group versus ΔDazl + Dazl mRNA group: p=0.3426. **** indicates $p<0.0001$.

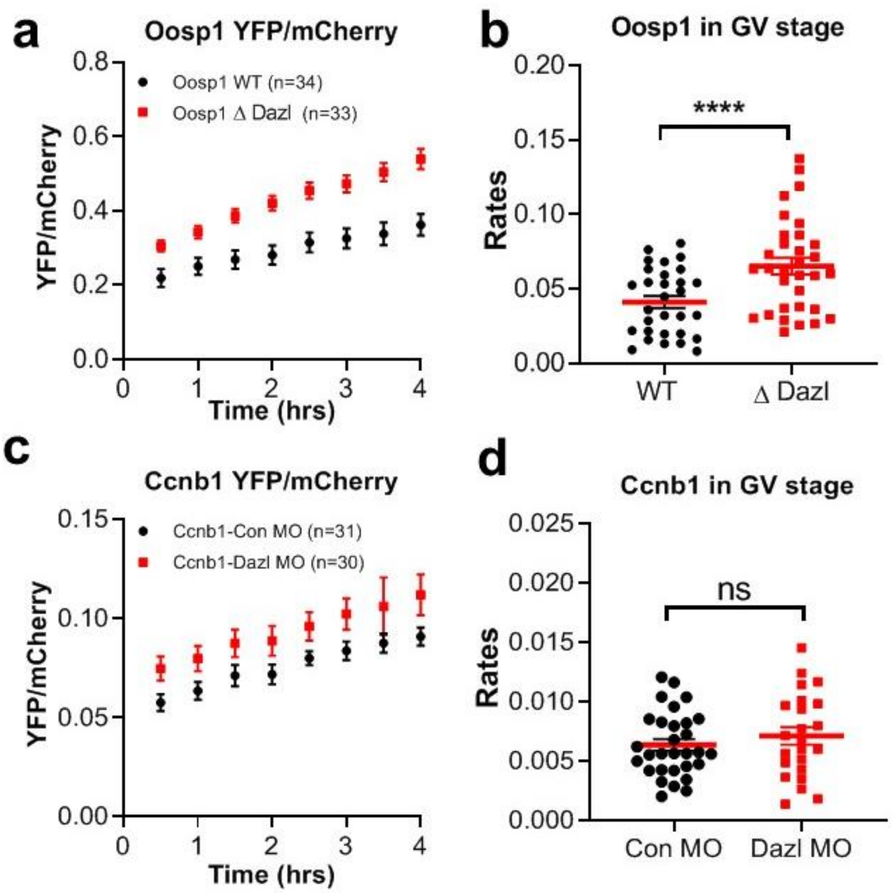

Supplementary Figure 13. Oosp1-Long accumulation in GV oocytes

Oocytes were injected with the YFP reporter fused to wild type Oosp1 3'UTR or 3'UTR with a mutated DAZL-binding element. After 3 hrs of preincubation for recovery, oocytes were maintained in GV with cilostamide and florescence was monitored by time lapse microscopy. Data are presented as the mean ± SEM (panel **(a)** and **(c)**) of each individual oocyte measurement. The the rates for each individual oocytes from n=3 independent biologically experiments is reported for panel **(b)** and **(d)**. Data were analyzed by unpaired two tailed t-test, panel b: $p < 0.0001$; panel d: $p=0.3777$. Rates of reporter accumulation were calculated as described in the Methods. **** indicated $p<0.0001$. The number of oocytes analyzed in each group is reported among brackets.

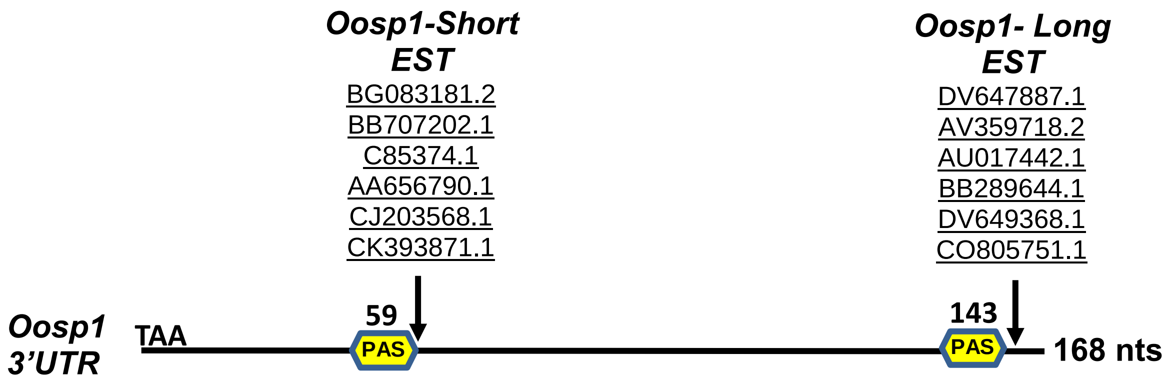

Supplementary Figure 14. Boundaries in Oosp1 3'UTR sequence defined by alignment with oocyte EST

The 3'UTR of Oosp1 was used to search for deposited expressed sequence tags (ESTs), and the boundary of hits is reported in the scheme. The arrows indicate the position of the boundary for the Oosp1-Short and Oosp1 Long 3'UTR. The boundary chosen for the long form corresponds to the NCBI reference Sequence NM_133353.3 Boundaries were confirmed by inspection of read density from Bam files of the mouse oocytes RiboTag RNA-Seq dataset.

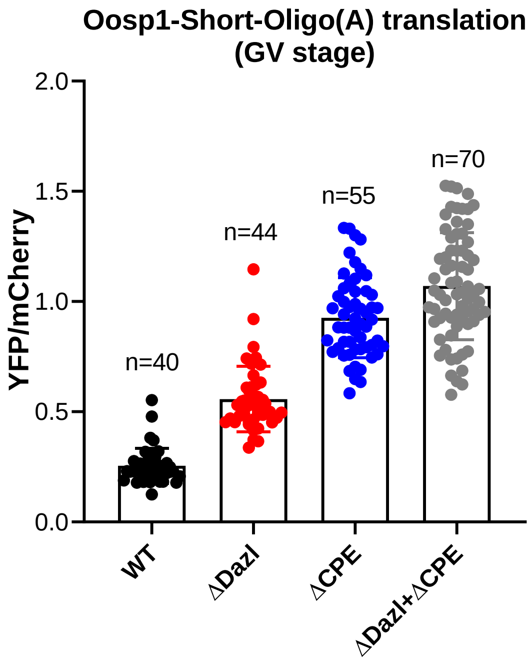

Supplementary Figure 15. Consequences of single or combined mutation of the CPE and Dazl element on the translation of the Oosp1-short reporter in GV stage oocytes.

The Oosp1 short wild type (WT) and mutant (ΔDazl, ΔCPE, and combined ΔDazl+ΔCPE) reporters were generated by fusing the 3'UTRs to YFP sequence and were injected in GV oocytes together with the *mCherry* constitutive reporter. After overnight recovery, oocytes were transferred to maturation medium and fluorescence recorded by time lapse microscopy for the first two hours of incubation prior to oocyte GVBD. Each experiment was repeated three or more times in different days and oocyte recordings were combined. Each point is the Mean ± SEM of single oocyte measurements. The total number of oocytes used for each construct is reported on the top of the bar.

Supplementary Table 1. Primers list

Name	Primers
Akap10 FW	AGTCATCAGATTCCCACCGAC
Akap10 REV	TGGCTTCTGTAATTGGTATTGGC
Cenpe FW	TGTCTGTGTTCGTGTGCGAC
Cenpe REV	AAGATTTACCCCCATCGCTCT
Ywhaz FW	AAAGGCAGGGCGTCATTCAG
Ywhaz REV	ACGGGGTTTCCTCCAATCAC
Nin FW	AGAACTCTATCCAGTGAGGAGC
Nin REV	TAGTGGCTCAAGCACTGTCAC
Dazl FW	GGATGAAACCGAAATCAGGA
Dazl REV	ATAGCCCTTCGACACACCAG
Oosp1 FW	TCTCTGGGGTTTGATCTTCAGC
Oosp1REV	CGTAGGCCTGATCCTTAGATGG
Obox5 FW	AGGGGATATCATGTTGGAGCC
Obox5 REV	GTTCCTTGCCGGTTCTTGAG
Tex19.1 FW	GGCTGTACCATCTTGTCCA
Tex19.1 REV	TCCTCTTCCTCTTCCTCCTC
Txnip FW	TGGACGACTCTCAAGACAGC
Txnip REV	CATTTCCTGCAGGCTCACTG
Tcl1 FW	TCGGAGTCCAACGATGAATAACC
Tcl1 REV	CTTCTTGGAGCCCAGTGTAGAG
Btg4 FW	TCGATCCCTATGAGGTGTGC
Btg4 REV	CCTGCTGCAGCTTTCTTCATC
Rad51C FW	TCAAGCTTTGCTTGTTCCATTA
Rad 51C REV	TATCGTAGACTCCTTCTGGCTTG
Ireb2 FW	TCAATGTACCTAAACTTGGCGG
Ireb2 REV	AAGGGCACTTCAACATTGCTC
Gdf9 FW	CAAACCCAGCAGAAGTCAC
Gdf9 REV	TTAAACAGCAGGTCCACCA
Ccnb1 FW	AAGGTGCCTGTGTGTGAACC
Ccnb1 REV	GTCAGCCCCATCATCTGCG
Dppa3 FW	GACCCAATGAAGGACCCTGAA
Dppa3 REV	GCTTGACACCGGGGTTTAG
Zp3 FW	TTTCGGCATTTCAAGTCCC
Zp3 REV	GGTGATGTAGAGCGTATTTCTG
Nlrp9b FW	ACC TCT GTA ATT CGA TGT CTC CAA C
Nlrp9b REV	CACACAGCTTCTCAGTCACATCCA
CPEB FW	TCGCTGCAAAAATAGTGCCC
CPEB REV	CTTGGTTGTCCCAGCAATCCT
Oog2 FW	CAGTTTTTGAAACCAGGAGTTGCAG
Oog2 REV	AGGTGTTGGATGATGAAGAGACTG
Oosp1 3'UTR FW	CATCACCATTGAatggtctggtgatttcttatctcc

Oosp1 3’UTR REV	GCGGGTTTAAACttagacacggcactaatggg
Oosp1 YFP FW	gtgccgtgtctaaGTTTAAACCCGCTGATCAGCCTC
Oosp1 YFP REV	tcaccagaccatTCAATGGTGATGGTGATGATGAC
Obox5 3’UTR FW	CATCACCATTGAcatatcagatgactggcttac
Obox5 3’UTR REV	GCGGGTTTAAACaaagaaatttaaatttactattttctcc
Obox5 YFP FW	ttaaatttctttGTTTAAACCCGCTGATC
Obox5 YFP REV	tcatctgatatgTCAATGGTGATGGTGATG
Cenpe 3’UTR FW	CATCACCATTGAatgcccctgtcccgtc
Cenpe 3’UTR REV	AGCGGGTTTAAACccttcaagacctttattcttcgc
Cenpe YFP FW	aaaggtcttgaaggGTTTAAACCCGCTGATCAGCCTC
Cenpe YFP REV	ggacaggggcatTCAATGGTGATGGTGATGATGAC
Nlrp9b 3’UTR FW	CATCACCATTGAtctgtgacaaaggcttatcctctg
Nlrp9b 3’UTR REV	GCGGGTTTAAACttttcaaacacgcttagtgtttattaaatg
Nlrp9b YFP FW	gcgtgtttgaaaaGTTTAAACCCGCTGATCAGCCTC
Nlrp9b YFP REV	cctttgtcacagaTCAATGGTGATGGTGATGATGAC

References

1. Chen, J. et al. Genome-wide analysis of translation reveals a critical role for deleted in azoospermia-like (Dazl) at the oocyte-to-zygote transition. *Genes & development* **25**, 755-766 (2011).
2. Luong XG, D.E., Rajkovic G, Yang C-R, Conti M in BioRxiv (2019).
3. Chen, H.H. et al. DAZL limits pluripotency, differentiation, and apoptosis in developing primordial germ cells. *Stem Cell Reports* **3**, 892-904 (2014).
4. Welling, M. et al. DAZL regulates Tet1 translation in murine embryonic stem cells. *EMBO Rep* **16**, 791-802 (2015).
5. Percharde, M., Bulut-Karslioglu, A. & Ramalho-Santos, M. Hypertranscription in Development, Stem Cells, and Regeneration. *Dev Cell* **40**, 9-21 (2017).
