# Supplementary material for: The RNA-binding protein DAZL functions as repressor and activator of mRNA translation during oocyte maturation
Source: Nat Commun. 2020 Mar 13;11:1399. doi: 10.1038/s41467-020-15209-9 (PMC7070028; doi:10.1038/s41467-020-15209-9)
Supplement: Supplementary file 2 — Description of Additional Supplementary Files [file 41467_2020_15209_MOESM2_ESM.pdf]

Description of Additional Supplementary Files

File Name: Supplementary Data 1
Description: List of transcripts whose translation is significantly affected by Dazl depletion

File Name: Supplementary Data 2
Description: List of transcripts significantly immunoprecipitated above background in a Dazl-RIP experiment
