# Supplementary material for: The RNA-binding protein DAZL functions as repressor and activator of mRNA translation during oocyte maturation
Source: Nat Commun. 2020 Mar 13;11:1399. doi: 10.1038/s41467-020-15209-9 (PMC7070028; doi:10.1038/s41467-020-15209-9)
Supplement: Supplementary file 3 — Supplementary Data 1 [file 41467_2020_15209_MOESM3_ESM.pdf]

	baseMean	log2FoldChange	lfcSE	stat	pvalue	padj
Mlh3	1934.427933	8.383650812	0.958396	8.747582	2.18E-18	1.79E-14
Fam69b	4878.031832	-7.383418221	0.894194	-8.25706	1.49E-16	6.15E-13
Dlgap2	3145.791244	7.393062876	0.959221	7.707365	1.28E-14	3.53E-11
Ccdc120	1721.537903	-7.224269229	1.003188	-7.20131	5.96E-13	1.23E-09
Tex264	1667.29294	-8.073341333	1.144463	-7.05426	1.74E-12	2.86E-09
Akt2	1842.578231	-6.851406961	0.976448	-7.01666	2.27E-12	3.12E-09
Pigb	1249.360328	-8.464945326	1.221039	-6.93258	4.13E-12	4.62E-09
Igfbp5	2781.173417	7.478908073	1.08064	6.920811	4.49E-12	4.62E-09
Socs4	3494.561147	6.055688034	0.886727	6.829257	8.54E-12	7.81E-09
Cd4	1316.666448	7.376603899	1.090056	6.767176	1.31E-11	9.64E-09
Gm3765	956.0613421	6.915908806	1.0234	6.757779	1.40E-11	9.64E-09
Smarcd2	1404.031413	-7.308902913	1.081603	-6.75747	1.40E-11	9.64E-09
Slc6a7	4521.216155	-5.83422797	0.871707	-6.69288	2.19E-11	1.39E-08
Bcl7c	1745.378997	-7.946487657	1.226631	-6.4783	9.28E-11	5.46E-08
Sdhd	1694.456495	-6.651601633	1.036557	-6.41701	1.39E-10	7.63E-08
Eif2b2	995.1282723	-7.133643471	1.118265	-6.37921	1.78E-10	8.83E-08
Pde10a	2749.389937	6.073831731	0.952669	6.375594	1.82E-10	8.83E-08
Gm24270	4347.629541	5.51009297	0.865786	6.364268	1.96E-10	8.98E-08
Fbxl18	664.594313	7.020198605	1.113522	6.304498	2.89E-10	1.25E-07
Rab3il1	1037.643528	-7.387434517	1.176383	-6.27978	3.39E-10	1.40E-07
Gna12	1230.614656	-6.630625083	1.06304	-6.23742	4.45E-10	1.74E-07
Dffa	812.7497988	-7.257029143	1.187047	-6.11351	9.75E-10	3.65E-07
Lrif1	1876.740506	5.564486412	0.916186	6.07353	1.25E-09	4.48E-07
Lmbr1	1646.816213	-5.76484252	0.956468	-6.02722	1.67E-09	5.72E-07
Fasn	2775.4378	-5.62676127	0.936538	-6.00805	1.88E-09	6.18E-07
Dab2ip	1803.741699	5.46518312	0.919897	5.94108	2.83E-09	8.97E-07
Irf2bp2	766.0661492	-7.435229678	1.253079	-5.93357	2.96E-09	9.04E-07
Kcnq1	834.2714478	-7.558729013	1.283268	-5.89022	3.86E-09	1.13E-06
Smarcb1	988.8758574	-7.804465583	1.328519	-5.87456	4.24E-09	1.17E-06
Rbm12	713.8132134	-7.333082701	1.248806	-5.87207	4.30E-09	1.17E-06
Lgals12	688.891857	-7.604439939	1.295737	-5.86882	4.39E-09	1.17E-06
Ap1m1	1323.096906	-7.084955979	1.209178	-5.85932	4.65E-09	1.17E-06
Klhl20	810.1812375	-6.835880465	1.166829	-5.85851	4.67E-09	1.17E-06
Cachd1	664.3829293	-7.552087999	1.293822	-5.83704	5.31E-09	1.29E-06
Nsmce1	828.0742546	-6.696757791	1.157318	-5.78644	7.19E-09	1.69E-06
Prr36	964.6828857	-7.505091751	1.307296	-5.74093	9.42E-09	2.15E-06
Hyal2	686.762423	-7.013206556	1.227077	-5.71538	1.09E-08	2.44E-06
Acyp1	778.2000635	-6.453896986	1.13505	-5.686	1.30E-08	2.77E-06
Rnf166	887.9133924	-7.971365757	1.402335	-5.68435	1.31E-08	2.77E-06
Pla2g6	692.9542761	-6.802893469	1.206789	-5.63718	1.73E-08	3.56E-06
Fmo5	893.3917232	-6.065582762	1.078121	-5.62607	1.84E-08	3.70E-06
Itgb1	593.0722576	-7.387777927	1.319774	-5.59776	2.17E-08	4.26E-06
Mrps2	914.663737	-6.005943568	1.075511	-5.58427	2.35E-08	4.49E-06
Spock1	1168.574703	5.628536021	1.008855	5.579131	2.42E-08	4.52E-06
Rasa4	2001.560803	-5.402050632	0.982596	-5.49773	3.85E-08	6.98E-06
Ttll1	804.9835757	-6.23833484	1.135203	-5.49535	3.90E-08	6.98E-06

Fau	681.8504027	-7.266670576	1.325101	-5.48386	4.16E-08	7.25E-06
Pdx1	1889.135835	-5.112124906	0.932661	-5.48122	4.22E-08	7.25E-06
Vamp8	596.0362525	-6.807902433	1.246358	-5.46224	4.70E-08	7.75E-06
Brsk2	596.1853216	-6.808260667	1.247145	-5.45907	4.79E-08	7.75E-06
Gcfc2	1834.924663	5.213772512	0.955446	5.456898	4.85E-08	7.75E-06
Ndufs2	826.2482333	-5.951985349	1.091095	-5.45506	4.90E-08	7.75E-06
Tpra1	2488.374101	-4.882188036	0.901852	-5.41351	6.18E-08	9.60E-06
Ier5l	801.7633063	-6.497118427	1.205001	-5.39179	6.98E-08	1.05E-05
Ccdc38	525.7749306	6.309689857	1.170463	5.390766	7.02E-08	1.05E-05
Rcl1	793.8568577	-7.809641248	1.450837	-5.38285	7.33E-08	1.08E-05
Plpp6	1216.646071	-5.60669674	1.043272	-5.37414	7.69E-08	1.11E-05
Stx8	585.5136846	-7.369318191	1.375451	-5.35775	8.43E-08	1.20E-05
Lactb2	595.7217216	-6.583651847	1.233341	-5.33806	9.39E-08	1.29E-05
Adcyap1r1	640.5731423	6.23604388	1.168279	5.337803	9.41E-08	1.29E-05
Ccdc62	1578.534079	-5.054964193	0.949784	-5.32223	1.03E-07	1.38E-05
Cdk20	1088.865258	-5.610068377	1.056617	-5.30946	1.10E-07	1.46E-05
Avpi1	696.550168	-6.154643098	1.161236	-5.30008	1.16E-07	1.51E-05
Fkbp8	787.6072882	-5.982707146	1.134783	-5.27212	1.35E-07	1.72E-05
Poll	723.2815421	-7.352028375	1.394844	-5.27086	1.36E-07	1.72E-05
2810004N	811.6736771	-5.743924463	1.091031	-5.26467	1.40E-07	1.75E-05
Thop1	1297.646017	-5.143919584	0.978734	-5.25569	1.47E-07	1.81E-05
Dis3	1354.078362	5.618073976	1.070789	5.246667	1.55E-07	1.88E-05
Thap11	1834.737491	-4.867628111	0.929038	-5.23943	1.61E-07	1.92E-05
Gm38227	557.5026339	6.403851089	1.230991	5.20219	1.97E-07	2.30E-05
Rad51c	1854.176093	-5.070079997	0.974833	-5.20097	1.98E-07	2.30E-05
Gns	761.5722121	-5.651138219	1.09879	-5.14306	2.70E-07	3.09E-05
Hsf2	726.1859945	5.761588861	1.126747	5.113473	3.16E-07	3.57E-05
Mrs2	735.475763	-6.233665063	1.224497	-5.0908	3.57E-07	3.97E-05
Pla2g12a	549.043183	-6.953164136	1.367332	-5.0852	3.67E-07	3.98E-05
Rabif	1130.126514	-5.215600825	1.025775	-5.08455	3.69E-07	3.98E-05
Mtmr12	544.2791223	-6.45273192	1.26957	-5.08261	3.72E-07	3.98E-05
Cd96	552.8999885	-7.28639456	1.436199	-5.07339	3.91E-07	4.13E-05
Zfp46	1047.600423	-5.441574897	1.074489	-5.06434	4.10E-07	4.24E-05
Pard6b	3008.233803	-4.464025776	0.881601	-5.06355	4.12E-07	4.24E-05
Asnsd1	448.4933033	-6.983283404	1.380359	-5.05903	4.21E-07	4.28E-05
Nutm1	1347.16664	5.001159453	0.992472	5.039093	4.68E-07	4.69E-05
Elk1	829.725468	-6.870457378	1.364907	-5.03365	4.81E-07	4.69E-05
Mib1	808.5687	-5.430337426	1.078851	-5.03345	4.82E-07	4.69E-05
Tbc1d20	1678.343438	4.68776454	0.931488	5.032554	4.84E-07	4.69E-05
Mrpl21	768.968247	-5.847398676	1.165319	-5.01785	5.23E-07	4.95E-05
Cox6b2	926.6815309	-5.434385375	1.083023	-5.01779	5.23E-07	4.95E-05
Dscr3	1384.112832	-6.801026469	1.358121	-5.00767	5.51E-07	5.15E-05
Tmco3	474.8250575	-7.066000047	1.416448	-4.98853	6.08E-07	5.57E-05
Meg3	533.1182042	-6.228809383	1.249066	-4.98677	6.14E-07	5.57E-05
Slc6a20b	624.8277688	-6.135272532	1.230468	-4.98613	6.16E-07	5.57E-05
Slc16a5	432.0288182	-6.929139014	1.392637	-4.97555	6.51E-07	5.82E-05
Anapc2	924.0329349	-5.153745285	1.039772	-4.95661	7.17E-07	6.30E-05

Gm7706	477.5134355	-6.750899215	1.362116	-4.95618	7.19E-07	6.30E-05
3110043O	695.4134549	-6.025706461	1.21761	-4.9488	7.47E-07	6.47E-05
9330175E:	488.0241159	-6.518098561	1.318705	-4.9428	7.70E-07	6.60E-05
Fra10ac1	474.7686571	-6.478048058	1.312155	-4.93695	7.94E-07	6.72E-05
Pcbp1	2157.383157	-4.509553855	0.913691	-4.93553	7.99E-07	6.72E-05
5530601H	557.9654185	-5.970662736	1.2106	-4.93199	8.14E-07	6.77E-05
Ccdc88c	636.5443306	-6.162272367	1.250238	-4.92888	8.27E-07	6.81E-05
Glb1l2	494.0146541	-6.311997209	1.288096	-4.90025	9.57E-07	7.80E-05
St18	896.0970598	4.987148704	1.01927	4.892864	9.94E-07	8.02E-05
Trpc5	603.4399818	-6.237738142	1.275371	-4.89092	1.00E-06	8.02E-05
Ago2	498.7065049	-6.32583211	1.297146	-4.87673	1.08E-06	8.54E-05
Golt1b	610.9108282	5.674206344	1.164917	4.870912	1.11E-06	8.71E-05
Col9a3	1188.561743	-5.088644738	1.046596	-4.86209	1.16E-06	9.02E-05
Fam72a	1184.701412	-4.874137856	1.003554	-4.85688	1.19E-06	9.18E-05
Larp1	619.8621152	-6.276733527	1.294586	-4.84845	1.24E-06	9.49E-05
Fth1	474.3015049	-6.2528824	1.290605	-4.84492	1.27E-06	9.57E-05
Mrpl58	510.6878103	-6.848059581	1.417063	-4.83257	1.35E-06	0.000101
Zfp472	554.0795637	-6.966405105	1.447179	-4.81378	1.48E-06	0.00011
Mtfr1	547.0123139	-5.941801763	1.235901	-4.80767	1.53E-06	0.000112
Oard1	558.0430106	-7.299590435	1.518941	-4.80571	1.54E-06	0.000112
Tfap2c	522.3359503	-6.027997174	1.255591	-4.80092	1.58E-06	0.000114
Mapre3	467.5611125	-6.720202783	1.4033	-4.78886	1.68E-06	0.00012
Sys1	423.6640858	-6.900878121	1.443467	-4.78076	1.75E-06	0.000124
B3gnt7	512.0644313	-5.845803896	1.225504	-4.77012	1.84E-06	0.00013
Nanos1	1284.007151	-4.645059194	0.97441	-4.76705	1.87E-06	0.00013
Htra1	461.0896059	-6.435594702	1.350948	-4.76376	1.90E-06	0.00013
Fam114a2	455.4799387	-6.417983227	1.347254	-4.76375	1.90E-06	0.00013
Vamp4	700.2265598	-6.300758287	1.326871	-4.74858	2.05E-06	0.000139
Pccb	2459.537344	-5.497633367	1.159489	-4.74143	2.12E-06	0.000143
Ppid	2406.08207	4.224633689	0.89307	4.73046	2.24E-06	0.00015
Srrt	480.356874	-6.495160309	1.374882	-4.72416	2.31E-06	0.000153
A430005L:	481.8688784	-6.499527921	1.379302	-4.71219	2.45E-06	0.000161
Trmt10b	1762.75131	-4.438913216	0.942869	-4.70788	2.50E-06	0.000164
Slc7a8	438.9803209	-6.952342655	1.47736	-4.70592	2.53E-06	0.000164
Xpa	2244.479884	4.207845193	0.897257	4.689678	2.74E-06	0.000176
Timd4	402.12407	-6.82509859	1.456593	-4.68566	2.79E-06	0.000178
Tollip	1929.851625	-4.78298918	1.021269	-4.68338	2.82E-06	0.000179
Fam162a	506.0314393	-7.15796396	1.53089	-4.67569	2.93E-06	0.000184
Pkp2	2115.353169	5.042645494	1.079644	4.670654	3.00E-06	0.000187
Emg1	1165.954526	-5.261134506	1.126731	-4.66938	3.02E-06	0.000187
Zfp618	679.4211481	-5.247448535	1.125861	-4.66083	3.15E-06	0.000194
Atp2b4	968.2472596	-5.326588647	1.14365	-4.65754	3.20E-06	0.000195
Hnrnpdl	2657.1726	-4.280660599	0.920157	-4.6521	3.29E-06	0.000199
Cenpt	419.7173288	-6.299281645	1.354584	-4.65034	3.31E-06	0.000199
Pxmp4	489.6861651	-6.522863514	1.403664	-4.64703	3.37E-06	0.000201
Zfp703	866.9330672	-6.472205454	1.397213	-4.63223	3.62E-06	0.000213
4921529L(	792.1290733	-5.890554726	1.271779	-4.63174	3.63E-06	0.000213

Hars	441.4175808	-5.954432276	1.289976	-4.61593	3.91E-06	0.000228
Kif21a	325.1848734	6.179029953	1.338918	4.614942	3.93E-06	0.000228
Ttc4	438.3416009	-5.944303995	1.289857	-4.6085	4.06E-06	0.000234
Wbp4	822.3145823	-5.142936021	1.116468	-4.60643	4.10E-06	0.000234
Elf1	899.0120676	-4.924328032	1.069266	-4.60533	4.12E-06	0.000234
Tspan33	2283.517618	-4.336004397	0.942428	-4.60089	4.21E-06	0.000237
Ell2	523.6777097	-6.031575229	1.317784	-4.57706	4.72E-06	0.000264
Bank1	3617.324795	4.306413726	0.942137	4.570899	4.86E-06	0.00027
Mgat5	914.6752979	-4.742557917	1.03885	-4.5652	4.99E-06	0.000274
Txnip	2360.999734	-4.176253874	0.915284	-4.56279	5.05E-06	0.000274
Figla	465.6976373	-5.707635727	1.251013	-4.56241	5.06E-06	0.000274
Lsm10	1391.043662	-4.418721034	0.968523	-4.56233	5.06E-06	0.000274
Siva1	1031.821275	-4.607127555	1.011087	-4.55661	5.20E-06	0.000279
Nr2c1	533.6562469	-5.767115356	1.265816	-4.55605	5.21E-06	0.000279
Tmem42	478.9260435	-6.073117295	1.335448	-4.54762	5.43E-06	0.000287
Med26	2499.768171	-5.053784409	1.11139	-4.54726	5.43E-06	0.000287
3830406C1	620.4223321	-5.635273705	1.242137	-4.53676	5.71E-06	0.0003
Plcg2	369.4843004	-6.378883094	1.406854	-4.53415	5.78E-06	0.000301
Pip5k1a	602.1957627	-7.086996136	1.567617	-4.52087	6.16E-06	0.000319
Rpl22l1	399.6899005	-6.492761197	1.438353	-4.51403	6.36E-06	0.000327
Mrpl9	398.7167932	-6.812739023	1.512925	-4.50302	6.70E-06	0.000343
Cdca7	455.935549	5.389086433	1.197256	4.501196	6.76E-06	0.000343
Dnal4	871.7298227	-5.018873201	1.119262	-4.48409	7.32E-06	0.00037
Ubac2	1795.660788	-4.370967881	0.976484	-4.47623	7.60E-06	0.000381
Sema5b	422.942621	5.626873351	1.257932	4.473114	7.71E-06	0.000385
Mrnip	672.7802735	-5.307943754	1.187224	-4.47089	7.79E-06	0.000386
Zfp964	394.0529515	-5.983608364	1.338971	-4.46881	7.87E-06	0.000388
Letm1	834.2651786	-4.814647901	1.078304	-4.46502	8.01E-06	0.000392
Foxc1	510.2946175	5.235254169	1.177438	4.446309	8.74E-06	0.000424
Jazf1	3591.879688	-4.015908644	0.903327	-4.44569	8.76E-06	0.000424
Rae1	1877.159729	-4.143795787	0.932641	-4.44308	8.87E-06	0.000427
Lpl	1232.003352	-4.899358508	1.103169	-4.44117	8.95E-06	0.000428
Pla2g15	688.7003615	-5.06284938	1.140533	-4.43902	9.04E-06	0.00043
AC118476.	727.8178912	-4.855452865	1.098021	-4.422	9.78E-06	0.000462
Tmem43	1563.943978	4.223849695	0.95552	4.42047	9.85E-06	0.000462
Pnpla2	1029.404151	-7.862544007	1.778926	-4.41983	9.88E-06	0.000462
Gltp	679.1900407	-5.87512676	1.330556	-4.41554	1.01E-05	0.000464
Csk	444.0053507	-5.638182806	1.27723	-4.41438	1.01E-05	0.000464
C2cd2	737.5167715	-4.823372496	1.0927	-4.41418	1.01E-05	0.000464
Mettl6	1510.58478	-4.31274831	0.977083	-4.4139	1.02E-05	0.000464
Trmt11	446.1663425	-5.970112611	1.353976	-4.40932	1.04E-05	0.000472
AW551984	718.9640434	4.800048484	1.090534	4.401558	1.07E-05	0.000486
Ugt8a	606.8454671	-5.954108243	1.353038	-4.40055	1.08E-05	0.000486
A330093E2	706.9425578	-4.812682116	1.095727	-4.39223	1.12E-05	0.000502
Hnf1b	332.7299839	-6.550496424	1.491977	-4.39048	1.13E-05	0.000503
Zfp628	541.9233902	-6.252661595	1.427144	-4.38124	1.18E-05	0.000521
Ssna1	662.8210856	-4.94427939	1.128799	-4.38012	1.19E-05	0.000521

Ino80e	911.8194579	-5.036710219	1.150112	-4.37932	1.19E-05	0.000521
Stk39	891.4699689	-5.718598768	1.308999	-4.36868	1.25E-05	0.000545
Pqbp1	782.5519748	-4.910377417	1.12537	-4.36335	1.28E-05	0.000555
Glrx5	781.7575883	-4.764281956	1.09225	-4.3619	1.29E-05	0.000556
Krt12	842.2215291	-4.621130346	1.060668	-4.35681	1.32E-05	0.000566
Apaf1	462.356419	-6.439767941	1.478818	-4.35467	1.33E-05	0.000569
Nup37	1404.33561	-4.240529308	0.97627	-4.3436	1.40E-05	0.000595
Cbr1	660.5370789	-4.821833104	1.11064	-4.34149	1.42E-05	0.000598
Eef1e1	537.779938	-6.658931786	1.534317	-4.34	1.42E-05	0.000599
Cenpm	558.4385814	-6.296036218	1.452102	-4.33581	1.45E-05	0.000606
Dusp16	348.8064972	-6.03037216	1.391175	-4.33473	1.46E-05	0.000606
Gemin7	313.7777153	-6.465531736	1.491831	-4.33396	1.46E-05	0.000606
Bcat1	497.6307496	-5.313546989	1.2267	-4.33158	1.48E-05	0.000609
Scarb1	1254.403434	4.379109584	1.011542	4.329141	1.50E-05	0.000613
Ildr1	454.3626826	-6.414223839	1.482566	-4.32643	1.52E-05	0.000618
Ddx17	1058.151746	-5.035689913	1.164842	-4.32307	1.54E-05	0.000624
Pdzd8	1832.5752	-3.986203948	0.922875	-4.31933	1.57E-05	0.000631
Exd2	242.0132656	6.324686761	1.464551	4.318515	1.57E-05	0.000631
Zfp691	1754.581075	3.967658639	0.921217	4.306976	1.66E-05	0.000662
Ap1m2	591.3932621	-5.565413785	1.301479	-4.27622	1.90E-05	0.000756
Cops7b	590.622206	-6.377376806	1.492059	-4.27421	1.92E-05	0.000759
Fcho1	454.6997451	-5.290051942	1.238159	-4.27251	1.93E-05	0.000761
Fbxo46	1246.454019	4.10385446	0.961213	4.269456	1.96E-05	0.000765
Thg1l	419.8341924	-6.887757086	1.613321	-4.2693	1.96E-05	0.000765
Pramef8	739.0080878	-4.826248506	1.131762	-4.26437	2.00E-05	0.000779
Sec11a	1484.122721	-4.415281163	1.037063	-4.25748	2.07E-05	0.000799
Metap1	432.8461514	-5.335032994	1.257789	-4.24159	2.22E-05	0.000854
Pold2	489.1096009	-6.521139227	1.539409	-4.23613	2.27E-05	0.000871
Preb	455.7450556	-6.418629269	1.515689	-4.23479	2.29E-05	0.000872
Dnmt3l	1922.806536	-4.353714136	1.030217	-4.22602	2.38E-05	0.000903
Tmem128	420.6669618	-5.559355366	1.319557	-4.21305	2.52E-05	0.000952
Klhl25	727.8547755	-6.218542697	1.476548	-4.21154	2.54E-05	0.000954
4833439L1	451.0593176	-5.661001057	1.345607	-4.20702	2.59E-05	0.000968
Ndufv2	298.0590637	-6.390986824	1.521354	-4.20085	2.66E-05	0.000991
Plcb3	427.4036772	-6.589968056	1.570442	-4.19625	2.71E-05	0.001002
Hspa2	709.688061	4.712298883	1.122994	4.196194	2.71E-05	0.001002
Sec24d	302.5017444	-6.412480328	1.531312	-4.18757	2.82E-05	0.001035
Tceanc2	494.987909	-5.021788323	1.199415	-4.18686	2.83E-05	0.001035
Twsg1	594.1864165	-4.783879824	1.14308	-4.18508	2.85E-05	0.001039
Abcb9	437.5724042	-6.359570941	1.520878	-4.18151	2.90E-05	0.001046
Tbc1d10b	527.5808649	-6.407565845	1.532366	-4.18149	2.90E-05	0.001046
D11Wsu47	916.1581905	-4.529109293	1.083426	-4.18036	2.91E-05	0.001047
Cacfd1	418.6405752	-5.413412674	1.295601	-4.1783	2.94E-05	0.001051
AC153816	335.1019749	-6.23714536	1.494749	-4.1727	3.01E-05	0.001073
Irf3	443.7798876	-6.380262988	1.529928	-4.1703	3.04E-05	0.00108
Git1	2604.522363	-4.6150362	1.107682	-4.16639	3.09E-05	0.001094
Actn3	729.6631506	-4.573734549	1.099945	-4.15815	3.21E-05	0.001122

Prkx	442.4527555	-5.367012068	1.29103	-4.15715	3.22E-05	0.001122
Tfap2a	984.6610339	-4.930066544	1.185966	-4.15701	3.22E-05	0.001122
Lrrc47	1269.027553	-4.07228195	0.97969	-4.15671	3.23E-05	0.001122
Bgn	1432.924993	4.168939082	1.003583	4.154056	3.27E-05	0.00113
Pdgfb	1797.637171	4.275411526	1.029757	4.151864	3.30E-05	0.001136
Ranbp3	3220.898886	-4.264897483	1.030331	-4.13935	3.48E-05	0.001195
9430021M	304.9916165	-6.424174745	1.555195	-4.13078	3.62E-05	0.001235
Gfra4	867.2643064	-4.415144942	1.069325	-4.12891	3.64E-05	0.00124
Inafm1	589.044412	-4.711122073	1.141499	-4.12714	3.67E-05	0.001245
Elk3	620.5121272	-5.635663359	1.367267	-4.12184	3.76E-05	0.001268
Kcnn2	4008.127179	3.534432135	0.858344	4.117735	3.83E-05	0.001286
Plagl1	1099.153676	4.036802223	0.981403	4.113295	3.90E-05	0.001306
Dctn3	699.1428955	4.581877913	1.114986	4.10936	3.97E-05	0.001322
Tmem178l	300.2806593	-6.077581567	1.479265	-4.10851	3.98E-05	0.001322
Mecom	405.4140546	5.071113661	1.235226	4.105413	4.04E-05	0.001335
Popdc2	416.2514415	-6.875084081	1.676591	-4.10063	4.12E-05	0.001357
Fkrp	514.3228852	-4.839681017	1.181028	-4.09785	4.17E-05	0.001368
Nacad	1202.578094	3.98858161	0.97399	4.095093	4.22E-05	0.001379
Tmem143	1538.215704	-5.582224164	1.363544	-4.09391	4.24E-05	0.00138
Anapc7	1053.156372	-4.091371901	1.00054	-4.08917	4.33E-05	0.001403
Nrcam	634.6757765	5.572718405	1.365978	4.079655	4.51E-05	0.001456
Dnm3	402.8484951	-6.504144193	1.60056	-4.06367	4.83E-05	0.001546
Mkl1	2769.946855	-3.677742225	0.905155	-4.06311	4.84E-05	0.001546
Cpsf4	479.6343119	-5.484959318	1.349977	-4.063	4.84E-05	0.001546
Cops7a	3565.696643	-3.773372352	0.930237	-4.05636	4.98E-05	0.001578
Usp11	2118.573795	-3.984415736	0.982404	-4.05578	5.00E-05	0.001578
Shisa8	345.7763983	-6.606485378	1.629014	-4.05551	5.00E-05	0.001578
Fam13b	660.2629551	-4.47036223	1.102511	-4.05471	5.02E-05	0.001578
Hectd3	2324.566219	-3.923739759	0.969306	-4.04799	5.17E-05	0.001617
Rfx1	337.9506844	-5.76019531	1.423525	-4.04643	5.20E-05	0.001622
Tifab	739.2785994	4.272221036	1.057389	4.040349	5.34E-05	0.001655
Dph7	367.2919833	-6.105289378	1.511227	-4.03996	5.35E-05	0.001655
I830077J0	1482.825398	-5.269376932	1.305029	-4.03775	5.40E-05	0.001664
Akr7a5	1975.062593	3.840839652	0.956817	4.014186	5.97E-05	0.001828
Hmgn1	1147.924207	-3.977845123	0.991022	-4.01388	5.97E-05	0.001828
Sgcd	513.0751401	-4.764490231	1.187673	-4.01162	6.03E-05	0.001833
Rgs19	394.7171722	-5.200225219	1.296305	-4.01157	6.03E-05	0.001833
Wbp1l	1496.337132	-3.967672588	0.992742	-3.99668	6.42E-05	0.00194
Tmem151a	607.9093748	-5.084821145	1.272366	-3.99635	6.43E-05	0.00194
Mthfsl	271.7591041	5.844092902	1.464224	3.991255	6.57E-05	0.001975
Zbtb14	386.0185664	-5.167703096	1.296545	-3.98575	6.73E-05	0.002014
Sri	337.750715	-5.983408792	1.501508	-3.98493	6.75E-05	0.002014
Mrpl3	959.6574375	-4.241476889	1.066289	-3.97779	6.96E-05	0.002068
Zbtb6	339.6656998	-5.991884197	1.506794	-3.97658	6.99E-05	0.002071
Dclre1c	3008.744365	4.515307095	1.136726	3.972205	7.12E-05	0.002102
Cpne3	4589.570696	3.652024229	0.919628	3.971196	7.15E-05	0.002103
Arhgef16	1113.878938	-3.914518169	0.987252	-3.96507	7.34E-05	0.002147

Ddx41	2287.680564	3.692379045	0.931346	3.964562	7.35E-05	0.002147
Mrpl48	1812.574097	-4.017455318	1.014482	-3.96011	7.49E-05	0.002176
Lysmd4	1587.175281	-3.759993652	0.949579	-3.95964	7.51E-05	0.002176
4930558C:	391.3696832	4.897254927	1.237049	3.958819	7.53E-05	0.002176
Rbm5	630.8227872	4.455565478	1.126956	3.95363	7.70E-05	0.002216
Tpgs1	256.7604828	-6.174336999	1.56328	-3.9496	7.83E-05	0.002246
Cnpy2	385.4367957	5.188675007	1.314384	3.947609	7.89E-05	0.002257
Ift46	334.948885	-6.560060508	1.663055	-3.94458	7.99E-05	0.002275
Pigc	1329.312799	-4.020936676	1.019644	-3.94347	8.03E-05	0.002275
Mrgbp	1256.744923	-4.094087822	1.038257	-3.94323	8.04E-05	0.002275
Pdlim2	523.0593181	-4.659002807	1.182759	-3.9391	8.18E-05	0.002304
Rps20	271.9288797	-5.933417101	1.506506	-3.93853	8.20E-05	0.002304
Cmklr1	2635.635323	-4.905427793	1.246419	-3.93562	8.30E-05	0.002324
Smco4	1796.199267	-3.697814025	0.940886	-3.93014	8.49E-05	0.002368
Rnf167	513.4757129	-5.467431486	1.391376	-3.92951	8.51E-05	0.002368
Zzef1	1088.492122	-6.352505805	1.619247	-3.92312	8.74E-05	0.002418
Pds5a	571.2142054	-4.499054255	1.146894	-3.92281	8.75E-05	0.002418
Sntg2	334.3901838	-5.37831268	1.372765	-3.91787	8.93E-05	0.002453
Dlgap1	563.4910837	-5.985116392	1.527695	-3.91774	8.94E-05	0.002453
Mrpl39	2384.995309	3.493177121	0.891989	3.916164	9.00E-05	0.002461
Ccnh	476.3795644	-5.054372302	1.29115	-3.91463	9.05E-05	0.002469
Dact3	566.1616646	-4.485948877	1.147814	-3.90826	9.30E-05	0.002526
Kctd2	340.8597356	-6.261628235	1.604438	-3.90269	9.51E-05	0.002577
Asna1	2897.545293	-3.488518256	0.897563	-3.88666	0.000102	0.002744
Ccdc158	761.5498747	-4.360658818	1.122479	-3.88485	0.000102	0.002747
Zmiz2	2071.631387	-4.002572014	1.030326	-3.88476	0.000102	0.002747
Rpl17	489.0605545	-4.625540527	1.191704	-3.88145	0.000104	0.002768
Pou2f2	10006.0564	3.877639144	0.99904	3.881364	0.000104	0.002768
1300002E:	2425.513929	3.679725995	0.950607	3.870922	0.000108	0.002877
Cd9	444.0351206	-5.045919066	1.303724	-3.87039	0.000109	0.002877
Pop4	835.1047156	-4.173970907	1.079803	-3.86549	0.000111	0.002926
AC107662	271.4723613	-6.255418044	1.620025	-3.86131	0.000113	0.002967
Csnk1g2	1167.907126	-3.87855612	1.006844	-3.85219	0.000117	0.00307
Klhl12	338.6433917	-5.987511456	1.555024	-3.85043	0.000118	0.003082
Rbbp5	3838.629466	3.404586592	0.884762	3.848027	0.000119	0.003103
Usp5	832.3706959	-4.003974232	1.042096	-3.84223	0.000122	0.003167
Nadsyn1	896.2163471	-3.964986239	1.032355	-3.84072	0.000123	0.003176
Snph	331.9422901	-5.734111223	1.493674	-3.83893	0.000124	0.00318
Gm45903	582.7191528	5.35668575	1.39544	3.838706	0.000124	0.00318
Strn4	1921.093494	-3.594646314	0.936554	-3.83816	0.000124	0.00318
Tpm1	902.0819553	-4.409080986	1.149961	-3.83411	0.000126	0.003216
R3hcc1l	1345.591337	-3.699426555	0.964933	-3.83387	0.000126	0.003216
Spa17	388.8393395	-5.770040817	1.505985	-3.83141	0.000127	0.003238
Arl15	963.1671003	-3.873365932	1.011922	-3.82773	0.000129	0.003277
Ttc38	719.1970079	-5.655961263	1.481789	-3.81698	0.000135	0.003412
Fbp2	503.0628452	-4.538939386	1.19058	-3.81238	0.000138	0.003466
Ano10	301.2034722	-5.81680308	1.526286	-3.81108	0.000138	0.003473

Cisd2	635.9222098	-4.206277173	1.106955	-3.79986	0.000145	0.003623
Nsdhl	2752.558495	4.00985017	1.05875	3.787344	0.000152	0.003799
Rab11fip3	562.1262198	-5.716272228	1.510749	-3.78373	0.000154	0.003843
Rit1	1008.40497	-4.262767825	1.128785	-3.77642	0.000159	0.003946
Nefh	486.2819818	4.602913772	1.219879	3.773255	0.000161	0.003984
Jmjd6	1276.10485	-3.729942309	0.98914	-3.77089	0.000163	0.00401
Prr5	1328.103598	-4.019591632	1.066396	-3.76932	0.000164	0.004023
Rpl41	1130.092082	-3.713711043	0.989924	-3.75151	0.000176	0.004297
Six1	508.8959664	5.292059778	1.410689	3.751401	0.000176	0.004297
Det1	342.9409287	-5.587058855	1.494624	-3.7381	0.000185	0.004517
B530045E1	231.9998085	-6.027055682	1.613132	-3.73624	0.000187	0.004537
Zfp612	666.5248002	4.015105055	1.075578	3.732976	0.000189	0.004583
Tmod2	1743.027118	-4.166289757	1.116307	-3.73221	0.00019	0.004583
Mapk14	2385.175823	3.745359715	1.004233	3.729574	0.000192	0.004618
Sbno1	1929.645355	-3.875967611	1.040329	-3.72571	0.000195	0.004675
Hpse	484.9245834	-4.424611412	1.188546	-3.72271	0.000197	0.004718
Gk5	1026.831051	-4.143851938	1.113952	-3.71995	0.000199	0.004738
Pkib	557.7737199	-6.488285306	1.74432	-3.71966	0.000199	0.004738
Slc25a26	247.3510582	-5.795527301	1.558177	-3.71943	0.0002	0.004738
Ndufb8	257.1430724	-5.586571635	1.503809	-3.71495	0.000203	0.0048
Cep44	317.0707086	-5.300492663	1.426909	-3.71467	0.000203	0.0048
Suds3	553.5278195	-4.255261477	1.145802	-3.71378	0.000204	0.004804
Gmcl1	2766.721359	-3.462575895	0.932605	-3.7128	0.000205	0.004809
S100a16	226.3807349	-5.991373358	1.61501	-3.70981	0.000207	0.004852
Ddx4	590.6961882	-4.177074614	1.127186	-3.70575	0.000211	0.004916
Josd1	1075.794664	-3.688535156	0.997541	-3.69763	0.000218	0.005062
Olfr650-ps	2337.006151	4.385554086	1.186817	3.695222	0.00022	0.005096
Cmtm6	292.5792831	-6.039715059	1.637508	-3.68836	0.000226	0.00522
Oosp1	3028.367837	-4.108584413	1.114621	-3.68608	0.000228	0.005253
Rps9	990.4188811	-3.834270375	1.042187	-3.67906	0.000234	0.005384
Ahsa2	447.8451784	4.530848362	1.232685	3.675593	0.000237	0.005443
Nphp4	1099.86673	3.637217808	0.989882	3.674395	0.000238	0.005451
Hist1h2aa	742.0165697	-3.913370699	1.065218	-3.67377	0.000239	0.005451
C130074G	580.4814133	-4.471090591	1.218063	-3.67065	0.000242	0.005486
Tnfsf13b	272.6791264	-5.447230844	1.484157	-3.67025	0.000242	0.005486
Umps	387.6611132	-5.59404098	1.524245	-3.67004	0.000243	0.005486
Gpm6a	1609.075774	-3.927482282	1.070368	-3.66928	0.000243	0.005487
Prkab2	1504.366216	4.170313074	1.139553	3.659605	0.000253	0.005683
Apip	1276.064741	-3.529485423	0.964925	-3.65778	0.000254	0.005708
Nprl2	568.8046424	-4.659620814	1.274667	-3.65556	0.000257	0.005742
Slc6a20a	907.0216794	-4.184615067	1.145699	-3.65246	0.00026	0.005796
Slc25a44	960.4901322	-3.693119675	1.013342	-3.64449	0.000268	0.005962
Hykk	462.9238548	-5.852192902	1.609609	-3.63579	0.000277	0.006151
Ndufa1	475.0551734	-5.144974033	1.415809	-3.63395	0.000279	0.006178
Cbfa2t3	963.3292913	-3.773297345	1.038918	-3.63195	0.000281	0.006209
St6gal1	1591.306446	-3.496077838	0.962882	-3.63085	0.000282	0.006219
Rnf139	1736.306097	-3.382453586	0.933597	-3.62303	0.000291	0.006393

Ube4a	2475.293802	3.272772204	0.903821	3.621041	0.000293	0.006426
Spata2	419.9065793	-4.617014141	1.275592	-3.61951	0.000295	0.006447
8430429K(	949.4417045	3.780989628	1.045281	3.617198	0.000298	0.006487
Inppl1	270.597289	-5.926451002	1.640015	-3.61366	0.000302	0.006559
Pcbp2	3960.561354	-3.284577759	0.909446	-3.61162	0.000304	0.006593
Pwwp2a	671.8201679	-4.287659249	1.187903	-3.60944	0.000307	0.006632
Ralgapa2	220.0384672	-5.950115686	1.648829	-3.60869	0.000308	0.006633
Celf3	216.6261746	-5.927230507	1.643496	-3.60648	0.00031	0.006673
Pdcd4	5325.094281	3.050809311	0.846083	3.605802	0.000311	0.006673
0610010F(	516.8264885	-4.29936514	1.194766	-3.5985	0.00032	0.006845
Pde1b	713.3283689	3.81343122	1.060222	3.596824	0.000322	0.006871
Gprasp1	352.7135749	4.963748566	1.38052	3.595565	0.000324	0.006887
Tfip11	1124.454291	-3.722209695	1.037144	-3.5889	0.000332	0.007047
Ndufa8	561.24583	-4.967626399	1.384812	-3.58722	0.000334	0.007071
Sphk2	1365.96375	-3.465791468	0.966367	-3.58641	0.000335	0.007071
BC005537	367.7110757	4.819199505	1.343884	3.586023	0.000336	0.007071
Tmem9	868.5511585	-4.120190792	1.150219	-3.58209	0.000341	0.00716
Sh3gl1	1595.627323	-3.815457381	1.069138	-3.56872	0.000359	0.007516
Mfap3l	552.9916102	-4.451292265	1.247618	-3.56783	0.00036	0.007522
Pinlyp	985.7988614	-3.659870031	1.027086	-3.56335	0.000366	0.007633
Zc3h3	712.0982742	-4.413144164	1.240876	-3.55647	0.000376	0.007815
Prdm6	937.9867626	-3.733491796	1.050613	-3.55363	0.00038	0.00788
Cep85l	1007.375404	-3.557254693	1.00332	-3.54548	0.000392	0.008108
H1f0	2130.798654	-3.334572019	0.940698	-3.54478	0.000393	0.008109
Tor1aip1	6530.606455	3.132834998	0.884441	3.542164	0.000397	0.008169
Lsm6	1049.251784	-3.555210076	1.005583	-3.53547	0.000407	0.008358
Papss2	327.906714	4.857972444	1.377798	3.525896	0.000422	0.008628
Rusc2	1202.621291	3.428280983	0.972351	3.525765	0.000422	0.008628
Slc37a3	341.3525757	-5.580300414	1.583797	-3.52337	0.000426	0.008684
Zfp939	1147.703394	-3.48721443	0.990372	-3.52112	0.00043	0.008737
Ddx23	738.5577606	-4.112850061	1.169816	-3.51581	0.000438	0.008891
Lrrc32	350.8606342	-6.627627219	1.886992	-3.51227	0.000444	0.008977
Ankrd50	309.8479569	-5.858258548	1.668353	-3.5114	0.000446	0.008977
BB557941	391.1705139	-4.59208965	1.3078	-3.51131	0.000446	0.008977
Rab5c	4156.67061	-3.129576503	0.892089	-3.50814	0.000451	0.009062
Dgke	1590.218843	-3.283370494	0.938046	-3.50022	0.000465	0.009313
Fbxw23	1007.937736	-3.94103432	1.130045	-3.4875	0.000488	0.009744
Mfsd13a	628.9301102	3.808142427	1.093225	3.483401	0.000495	0.009871
Aig1	3963.775036	3.493647778	1.004042	3.479584	0.000502	0.009988
S100pbp	1110.181621	3.418051091	0.984117	3.473215	0.000514	0.010203
Gm5	339.7874905	-5.108470899	1.471401	-3.47184	0.000517	0.010231
Mmp2	270.272494	-5.434225878	1.566853	-3.46824	0.000524	0.010344
Mcat	375.0219087	-4.450360432	1.284245	-3.46535	0.00053	0.010431
Nufip1	409.6996711	-5.254597735	1.517164	-3.46344	0.000533	0.010467
Dnm2	943.4301433	-3.507908191	1.012928	-3.46314	0.000534	0.010467
Tead3	287.4358399	5.128245358	1.481425	3.461699	0.000537	0.010492
Eef1b2	729.7732399	-4.15960959	1.20177	-3.46123	0.000538	0.010492

Ebna1bp2	914.8982663	-4.068178834	1.175788	-3.45996	0.00054	0.010498
Tox	4285.449299	4.241815997	1.226032	3.459791	0.000541	0.010498
Fbxl7	580.07329	-6.545128027	1.893345	-3.45691	0.000546	0.01058
Minos1	567.6047662	-4.037655036	1.168153	-3.45644	0.000547	0.01058
Dyrk1b	908.9362725	-5.746711966	1.666228	-3.44893	0.000563	0.010853
2700099C1	338.694714	-4.553496183	1.321128	-3.44667	0.000568	0.010902
Gm7073	308.0502123	-5.258555311	1.525789	-3.44645	0.000568	0.010902
Rab4a	3125.805442	3.310077498	0.961209	3.443659	0.000574	0.010989
Farp1	3686.039352	-3.158134666	0.918575	-3.43808	0.000586	0.011192
Zfp341	718.2687223	-4.238475597	1.233501	-3.43613	0.00059	0.011247
Tmem135	1229.793809	-3.329852204	0.969394	-3.43498	0.000593	0.011269
Mlh1	4681.115126	3.489907982	1.016414	3.433549	0.000596	0.011286
Psd2	2037.975946	4.46740051	1.301192	3.433313	0.000596	0.011286
2810021J2	194.5823911	-5.771132732	1.682472	-3.43015	0.000603	0.011393
Gm11827	3120.315175	-3.207640428	0.935668	-3.42818	0.000608	0.011449
Prr13	225.722787	-5.662201907	1.652934	-3.42555	0.000614	0.011534
Slc25a1	368.8634695	-4.425929486	1.292918	-3.42321	0.000619	0.011605
Exosc7	266.0073952	-5.635801628	1.646621	-3.42265	0.00062	0.011605
Adgrl2	834.4728357	-3.835157605	1.122089	-3.41787	0.000631	0.011784
AC156832	856.2102274	-3.520495535	1.031142	-3.41417	0.00064	0.011918
Ncoa5	833.6799458	-3.537161208	1.03652	-3.41254	0.000644	0.011963
Agpat3	563.9509911	-4.067706074	1.19541	-3.40277	0.000667	0.012371
Ggta1	7290.513539	-2.857629803	0.84009	-3.40157	0.00067	0.012397
Dennd1a	5111.055482	-2.989308073	0.880007	-3.39691	0.000682	0.012582
Utp11	560.0502559	-3.941378129	1.160804	-3.39539	0.000685	0.012616
Timm17a	2786.033304	-3.017993815	0.888967	-3.39494	0.000686	0.012616
Gm44621	552.3258705	-3.883842775	1.144481	-3.39354	0.00069	0.012653
Rit2	1828.634223	4.6724943	1.377233	3.392669	0.000692	0.012665
Cap1	736.8407129	3.832725161	1.13193	3.386008	0.000709	0.012948
Ranbp1	501.0866384	-4.359453972	1.288214	-3.38411	0.000714	0.013009
Sssca1	481.2248839	-6.497581528	1.923841	-3.3774	0.000732	0.013292
Pts	670.3370248	4.213569477	1.247906	3.376513	0.000734	0.013292
Cldn8	396.4962358	-4.315942861	1.278325	-3.37625	0.000735	0.013292
Ppm1l	1171.132654	-3.302403375	0.978271	-3.37576	0.000736	0.013292
Ralgds	1111.096693	-3.333130171	0.987781	-3.37436	0.00074	0.013331
0610012G	596.6259229	-3.827180291	1.134832	-3.37246	0.000745	0.013363
Rpa3	755.9788492	-3.941072068	1.16877	-3.37198	0.000746	0.013363
Nfkbiz	1483.103217	-3.409761196	1.01123	-3.3719	0.000747	0.013363
Dab2	20664.09604	2.7680743	0.821615	3.369065	0.000754	0.013472
Pnpla6	2254.47708	-3.12447511	0.928512	-3.36503	0.000765	0.01364
Vps8	1660.592177	3.107501945	0.92505	3.35928	0.000781	0.013897
Crtc1	671.10379	-3.682395037	1.100827	-3.34512	0.000822	0.014595
Nedd8	1779.750917	-3.550958131	1.06221	-3.34299	0.000829	0.014676
Chmp1b	1199.030396	3.573589458	1.069863	3.340231	0.000837	0.014755
Tsga10	1246.659351	-3.481829023	1.042418	-3.34015	0.000837	0.014755
Trmt10a	509.6655784	-3.957893195	1.185101	-3.33971	0.000839	0.014755
Dtx3l	489.9312152	3.863266857	1.157797	3.336738	0.000848	0.014862

Zfp345	5902.079685	2.893431059	0.867201	3.336516	0.000848	0.014862
Lima1	810.8969418	-3.554439979	1.06562	-3.33556	0.000851	0.014882
Ttc39aos1	335.468519	-6.238739927	1.872231	-3.33225	0.000861	0.015028
Idh1	212.1581636	-5.305908731	1.592882	-3.33101	0.000865	0.015063
Obox5	23058.25417	-2.906569475	0.872953	-3.32958	0.00087	0.015082
Mfn2	1649.366273	-3.464004984	1.040501	-3.32917	0.000871	0.015082
Zfhx2	284.630813	-4.720867415	1.418143	-3.32891	0.000872	0.015082
Dnah14	337.3537794	-6.570690465	1.974894	-3.32711	0.000878	0.015148
Haus1	318.2550669	-5.012328944	1.506966	-3.32611	0.000881	0.015171
Gls	515.016839	3.795044728	1.141676	3.324098	0.000887	0.015248
Map3k12	833.3999179	3.413828974	1.027415	3.322737	0.000891	0.015285
Kpna6	16102.75657	2.907069242	0.87516	3.321759	0.000895	0.015285
Rc3h2	205.5405115	-5.851033407	1.761467	-3.32168	0.000895	0.015285
Stim2	987.5528794	-3.329827037	1.00329	-3.31891	0.000904	0.015406
Zc3h7b	1301.129389	-3.201320208	0.965713	-3.31498	0.000916	0.015592
Phtf2	8764.761641	-2.860717397	0.86368	-3.31224	0.000926	0.015713
Mtif3	450.747403	-4.148681118	1.253166	-3.31056	0.000931	0.015775
Selenow	683.8535422	-3.906333524	1.181578	-3.30603	0.000946	0.015999
Igkv13-87	202.0013978	5.129620708	1.552239	3.30466	0.000951	0.016045
Adam32	221.173137	-5.141458705	1.556482	-3.30326	0.000956	0.016092
Gnptg	571.552728	-4.302794632	1.303587	-3.30073	0.000964	0.016205
Hvcn1	2342.201703	-3.840416601	1.164386	-3.29823	0.000973	0.016316
Gm29595	308.444087	-4.612268683	1.399692	-3.2952	0.000984	0.01646
Bcl7b	333.2365483	-5.219425016	1.584586	-3.29387	0.000988	0.016504
Rps6ka3	3064.295791	3.812607216	1.157892	3.292715	0.000992	0.016539
Cluap1	260.7957346	-5.382427107	1.63608	-3.28983	0.001002	0.016675
Rheb	828.3475155	-3.527454316	1.074698	-3.28227	0.00103	0.017094
Snrpd3	398.468373	-5.341111766	1.627583	-3.28162	0.001032	0.0171
Rab3b	1282.978646	-3.262078525	0.995663	-3.27629	0.001052	0.017391
Tarbp2	374.0321273	-5.004454585	1.529965	-3.27096	0.001072	0.017686
Tbrg1	476.4104972	-5.054671504	1.548969	-3.26325	0.001101	0.018138
Ak7	320.394487	-6.171965091	1.892627	-3.26106	0.00111	0.018243
Ppp6r1	938.2910507	-5.176072686	1.588057	-3.25937	0.001117	0.018315
Sel1l	4875.537331	2.788314173	0.857451	3.251865	0.001147	0.018768
Rab1b	750.994525	-3.438851181	1.058339	-3.24929	0.001157	0.018901
Dhcr7	452.1139597	3.828386499	1.179583	3.245542	0.001172	0.019114
Dsc2	616.1281717	-3.908096916	1.204481	-3.24463	0.001176	0.019137
Kin	1352.397457	3.41004046	1.053024	3.238332	0.001202	0.019512
Axin1	964.02433	-4.329683779	1.337159	-3.23797	0.001204	0.019512
Hspb11	296.7566913	4.755732963	1.469647	3.235969	0.001212	0.019611
Braf	1532.737958	3.50864398	1.085353	3.232723	0.001226	0.019797
Tubb3	5546.167122	-2.743619709	0.848937	-3.23183	0.00123	0.01982
Lhb	408.2801029	-4.922848676	1.524552	-3.22905	0.001242	0.019975
Cenpi	543.2615329	4.094248429	1.269074	3.22617	0.001255	0.020114
Ninj1	1793.847714	-3.039518794	0.942213	-3.22593	0.001256	0.020114
Anapc11	556.1822176	-3.658469989	1.134301	-3.22531	0.001258	0.020119
Ubb	5551.706454	-2.74161226	0.850582	-3.22322	0.001268	0.020227

Gm14085	679.4831166	-5.176118757	1.607285	-3.22041	0.00128	0.020387
Ift140	836.8734506	-3.960748069	1.232852	-3.21267	0.001315	0.020904
Sass6	184.4843853	5.102592577	1.589043	3.211111	0.001322	0.020972
Tril	699.1427959	-4.905083881	1.527761	-3.21064	0.001324	0.020972
Extl1	196.9142564	-5.463133707	1.701895	-3.21003	0.001327	0.020976
Tbl3	926.6486812	-3.566852399	1.111461	-3.20916	0.001331	0.020999
Mthfd2	1465.609837	-3.042497058	0.949011	-3.20597	0.001346	0.021188
Apool	223.1734467	-5.970410689	1.862567	-3.20547	0.001348	0.021188
C130073F1	312.4128844	-4.344868942	1.356947	-3.20194	0.001365	0.021409
AC149586.	753.7896499	-3.964752803	1.238552	-3.20112	0.001369	0.02143
Fam217a	7422.53967	2.67757744	0.836991	3.199051	0.001379	0.021543
Trim35	249.9842639	4.997630258	1.563795	3.195835	0.001394	0.021743
Itsn1	5785.627458	2.849637137	0.892529	3.192766	0.001409	0.021894
Rcc2	2512.582928	-3.278423289	1.026836	-3.19274	0.001409	0.021894
Asah2	1474.238837	3.210956827	1.006108	3.191464	0.001416	0.02195
Ppef1	1716.340839	2.942322451	0.922225	3.190462	0.00142	0.021955
Otub2	2295.254576	-3.869664127	1.212943	-3.19031	0.001421	0.021955
Inpp5b	896.8101494	-3.287321003	1.030648	-3.18957	0.001425	0.021971
Slc38a7	227.3603442	-5.181633512	1.625061	-3.18858	0.00143	0.022005
Fgf1	700.5009857	-3.720682129	1.16722	-3.18765	0.001434	0.02202
Gm11639	993.1997382	3.871491599	1.214664	3.187296	0.001436	0.02202
4930522H	649.0399504	-4.4905631	1.410726	-3.18316	0.001457	0.022296
Kif1c	468.0983476	-3.87308627	1.21758	-3.18097	0.001468	0.022423
Erg	4657.891926	2.806009193	0.882507	3.179588	0.001475	0.022489
Cntnap3	525.5238526	3.608258589	1.135255	3.178368	0.001481	0.022542
Ccdc93	1092.494065	-3.320401006	1.045443	-3.17607	0.001493	0.022679
Rdh12	7456.349011	2.675987511	0.843239	3.173462	0.001506	0.022842
Ppan	1127.398997	-3.305172025	1.042051	-3.17179	0.001515	0.022928
Adhfe1	848.8753136	-3.884210042	1.2248	-3.1713	0.001518	0.022928
Rasl11b	394.6670218	-4.605140924	1.452472	-3.17055	0.001521	0.022945
Tgoln1	1770.78992	3.197268309	1.009271	3.167898	0.001535	0.023094
Skiv2l2	8507.071722	3.001521214	0.947568	3.167605	0.001537	0.023094
Rps5	322.9731388	-4.229394027	1.338082	-3.16079	0.001573	0.023598
Ndufc1	1559.74504	3.095621444	0.979592	3.160113	0.001577	0.02361
Gm19757	347.9912234	4.117618867	1.303431	3.159063	0.001583	0.023653
Nlrp9a	85735.02465	2.575807305	0.815963	3.156771	0.001595	0.023796
Nudt2	207.1803899	-5.537576439	1.755482	-3.15445	0.001608	0.023943
Mrpl50	780.8560682	3.777320827	1.199127	3.150059	0.001632	0.024262
Ednrb	250.6003543	-4.661555325	1.480705	-3.1482	0.001643	0.024364
Rpp21	349.8277445	-4.600986012	1.461663	-3.14777	0.001645	0.024364
BC024978	238.7505492	4.725631456	1.501694	3.146867	0.00165	0.024396
Esr2	2508.985916	2.880742469	0.916155	3.144382	0.001664	0.02456
Gm12328	485.3047703	-3.690425343	1.174089	-3.14322	0.001671	0.02457
Atp6v0a1	607.8868864	-4.393890672	1.397898	-3.14321	0.001671	0.02457
Agk	652.0210551	-3.562179859	1.133582	-3.14241	0.001676	0.024594
Adm2	1340.954991	-3.318689438	1.056709	-3.14059	0.001686	0.024699
Tomm22	1572.865461	-2.932948777	0.934024	-3.14012	0.001689	0.024699

Dfna5	2019.691903	2.86378416	0.912235	3.139306	0.001693	0.024724
Gm15592	165.4968981	-5.535219879	1.76406	-3.13777	0.001702	0.024809
Trmt12	1473.850487	4.056060671	1.293987	3.134546	0.001721	0.02504
Ogg1	911.9507354	-4.858857432	1.550671	-3.13339	0.001728	0.025094
Trp73	295.3976465	-5.196936658	1.661334	-3.12817	0.001759	0.025466
Crb3	240.7171542	-5.265069386	1.683188	-3.12803	0.00176	0.025466
Mrpl13	557.2502091	-5.830077077	1.86484	-3.12632	0.00177	0.02557
Npat	1628.48187	2.963690397	0.948286	3.125313	0.001776	0.025613
Atic	297.2940615	-4.271429383	1.36908	-3.11993	0.001809	0.02604
Ikbkb	446.4323899	-3.802205557	1.221174	-3.11357	0.001848	0.026562
Gm7353	549.1574475	-4.243641703	1.364429	-3.1102	0.00187	0.02682
Chtop	707.2591981	-3.328116843	1.070459	-3.10906	0.001877	0.026877
Cct4	531.614931	3.742180191	1.203849	3.108512	0.00188	0.026879
Zswim4	812.8890967	-3.266041849	1.051509	-3.10605	0.001896	0.027057
Hist1h1a	950.5734195	-3.81273489	1.228304	-3.10406	0.001909	0.027146
Dpysl5	800.3876771	3.444284648	1.109609	3.104053	0.001909	0.027146
Gid8	594.0349164	3.518173045	1.134131	3.102086	0.001922	0.02728
Rnase6	1180.826216	3.011870851	0.971456	3.100368	0.001933	0.027392
Dpy19l3	1282.032772	-3.053550444	0.986582	-3.09508	0.001968	0.027837
Eif2b4	1250.147381	-3.024362716	0.978006	-3.09238	0.001986	0.028009
St3gal5	862.3896153	4.173579232	1.349698	3.092233	0.001987	0.028009
Plekho1	247.9214053	-4.517647684	1.461586	-3.09092	0.001995	0.028085
Klf7	4637.974746	2.871904207	0.929723	3.08899	0.002008	0.02822
Eif3k	1253.930152	-3.105381494	1.005734	-3.08768	0.002017	0.028231
Rps3a1	261.2325634	-4.862565831	1.574887	-3.08756	0.002018	0.028231
Trmt1l	1579.583702	2.964139084	0.960089	3.08736	0.002019	0.028231
Nmnat2	179.1337349	-5.65045478	1.834379	-3.08031	0.002068	0.028859
Col6a5	1308.748884	3.262783848	1.059487	3.079589	0.002073	0.02888
Gm5177	1303.667817	-3.204315892	1.041501	-3.07663	0.002094	0.029078
Csnk1d	1791.981228	-3.263049287	1.060697	-3.07633	0.002096	0.029078
2610507B1	2382.564111	2.753950457	0.895309	3.075977	0.002098	0.029078
Arhgap5	1159.266684	3.756341884	1.221358	3.075545	0.002101	0.029078
Gtpbp1	1323.917242	-2.986687979	0.97137	-3.07472	0.002107	0.029109
Lrp11	896.8625619	-3.685949519	1.199906	-3.07186	0.002127	0.02934
U2af2	505.7610358	-3.86587868	1.259198	-3.07011	0.00214	0.029463
Gm15564	2748.453392	2.703951836	0.881288	3.068182	0.002154	0.029605
Tmem150a	589.9304655	-3.68550691	1.20256	-3.06472	0.002179	0.0299
Lypd6	671.4592455	-3.373926256	1.102362	-3.06063	0.002209	0.03026
Slc25a15	2817.923019	-2.745580887	0.897654	-3.05862	0.002224	0.030414
Cdpf1	1056.618465	-3.348873243	1.095399	-3.05722	0.002234	0.030506
Add2	638.9364732	-3.38782922	1.108957	-3.05497	0.002251	0.030676
Gpr27	305.2230711	-5.416818628	1.773358	-3.05456	0.002254	0.030676
Fpgs	986.4935532	-3.065219476	1.003827	-3.05353	0.002262	0.03073
Smim26	298.096436	-4.190837178	1.373484	-3.05125	0.002279	0.030914
Krcc1	1737.696522	-2.864223602	0.939219	-3.04958	0.002292	0.03102
Ccdc170	1603.325139	3.786065229	1.241645	3.049233	0.002294	0.03102
Cacna1b	172.0008428	-5.591245133	1.834619	-3.04763	0.002307	0.031134

Rpl22	1307.435647	-2.924896288	0.960898	-3.04392	0.002335	0.031469
Cul4a	3057.64422	3.185911187	1.046912	3.04315	0.002341	0.031498
Prkrip1	3028.458483	-2.672608997	0.879209	-3.03979	0.002367	0.0318
Bbip1	987.0416636	-3.245596762	1.067922	-3.03917	0.002372	0.031814
Ppif	1337.74602	-3.184674384	1.050226	-3.03237	0.002426	0.032486
Rps3	920.4603453	-3.487681871	1.152522	-3.02613	0.002477	0.033081
Shroom3	4390.153222	-3.186163594	1.05296	-3.02591	0.002479	0.033081
Oxa1l	172.3681301	-5.268753569	1.742496	-3.02368	0.002497	0.033272
Alg8	6006.785471	2.566973487	0.849953	3.020137	0.002527	0.033609
Cux2	227.2543505	-4.812981036	1.594258	-3.01895	0.002537	0.033687
Rabl6	365.6235508	4.320495618	1.431577	3.017998	0.002545	0.033738
Cmc2	279.9305123	-4.578504176	1.518644	-3.01486	0.002571	0.034034
Lmna	309.2151363	-4.615882446	1.531387	-3.01418	0.002577	0.034045
Zmat3	823.0745905	3.175950262	1.053805	3.013793	0.00258	0.034045
Eif3h	1350.004252	-3.060137057	1.016237	-3.01124	0.002602	0.034277
Pskh1	1293.363519	-3.295503369	1.094665	-3.01051	0.002608	0.034305
Slc25a38	735.273653	-3.693187327	1.228018	-3.00744	0.002635	0.034561
Map1s	1182.758793	-3.353234171	1.115037	-3.00728	0.002636	0.034561
Pygo2	488.4670282	-3.894425416	1.295427	-3.00629	0.002645	0.034619
Gm14004	236.5613563	-4.448483252	1.481363	-3.00297	0.002674	0.034944
AC101677.	428.1571756	-3.827628626	1.275677	-3.00047	0.002696	0.035176
Ptpdc1	1943.710223	2.846683366	0.950168	2.995978	0.002736	0.035641
Adam7	335.058853	3.837186149	1.282627	2.991662	0.002775	0.036092
Cpox	799.8071344	-3.327216802	1.112401	-2.99102	0.00278	0.036111
Prmt5	177.1023325	-5.308618815	1.776564	-2.98814	0.002807	0.036396
Mlx	790.5394716	-3.142337525	1.052244	-2.98632	0.002824	0.036556
Klf16	1015.400754	-2.981293433	0.99859	-2.9855	0.002831	0.036596
Lrp2	1213.666085	3.226780948	1.082615	2.980543	0.002877	0.037131
Nucb1	2138.220531	2.742330626	0.920214	2.9801	0.002882	0.037131
Gpr63	243.4127546	-4.913467768	1.649704	-2.97839	0.002898	0.037229
Tm9sf3	1462.036239	2.815885089	0.945458	2.978331	0.002898	0.037229
Olfm1	803.6939978	-3.623510802	1.216849	-2.97778	0.002903	0.037238
Wdr92	493.5700143	-3.478929663	1.169195	-2.97549	0.002925	0.037459
Rpl32	1184.962249	-2.905124875	0.976968	-2.97361	0.002943	0.03763
Hid1	2986.29705	2.675139441	0.900287	2.971429	0.002964	0.03784
Aff3	1655.87171	2.782485382	0.937276	2.968692	0.002991	0.038111
Cnih4	1679.829262	-2.874191339	0.968298	-2.96829	0.002995	0.038111
1700084F2	525.7481699	3.535760734	1.191527	2.967419	0.003003	0.03816
Aasdhppt	772.5405541	3.157551065	1.064275	2.966858	0.003009	0.038171
Limd2	379.119372	-3.739360575	1.261109	-2.96514	0.003025	0.038326
Trim30d	346.9174229	4.377518462	1.477882	2.962022	0.003056	0.038619
Trim31	550.0577063	-3.464634954	1.169793	-2.96175	0.003059	0.038619
Cd55	38194.93125	2.518325986	0.850391	2.961372	0.003063	0.038619
C77370	749.6503049	3.109201693	1.050345	2.960172	0.003075	0.038711
Lsm3	319.8143151	-3.931057506	1.328207	-2.95967	0.00308	0.038715
Ccdc122	229.2404776	-4.530347512	1.531912	-2.95732	0.003103	0.038952
Lhfp	836.353525	-3.621016064	1.225855	-2.95387	0.003138	0.03933

Zfp553	1286.012774	2.917293137	0.987896	2.953037	0.003147	0.039337
Atp5g1	210.0773279	-4.697461185	1.590809	-2.95288	0.003148	0.039337
Mipep	2484.066307	-3.052131684	1.034125	-2.95141	0.003163	0.039464
Mapk11	380.422054	-3.896606155	1.320677	-2.95046	0.003173	0.039526
Zfp541	212.0482637	-5.571071457	1.890823	-2.94637	0.003215	0.039991
Gm44398	972.1615698	-3.030708289	1.03017	-2.94195	0.003262	0.040506
Nqo1	546.2204793	-3.346180867	1.137665	-2.94127	0.003269	0.040534
Cpb1	277.5497935	-4.951749337	1.684409	-2.93976	0.003285	0.040604
Arhgef7	7327.229747	-2.689831716	0.91519	-2.9391	0.003292	0.040604
Ctif	1835.865221	-2.930696258	0.997211	-2.93889	0.003294	0.040604
Ubqln4	2921.836056	-2.619901249	0.891465	-2.93887	0.003294	0.040604
Vps37c	1260.668411	-3.133334979	1.0666	-2.93768	0.003307	0.040625
Mau2	1380.196685	-2.910459569	0.990779	-2.93755	0.003308	0.040625
Cenpe	52668.96538	2.414792907	0.822108	2.93732	0.003311	0.040625
Ssr2	710.0660571	3.161827286	1.077241	2.935117	0.003334	0.040854
Cyth2	583.6054811	-3.420336026	1.166386	-2.93242	0.003363	0.041082
Tubd1	628.6057027	-3.295948304	1.124103	-2.93207	0.003367	0.041082
Gatsl2	409.148687	-4.233852067	1.444012	-2.93201	0.003368	0.041082
Upp1	1271.371368	-3.346099322	1.141697	-2.93081	0.003381	0.04118
Omt2b	5700.074863	-2.473073426	0.84477	-2.92751	0.003417	0.041558
Gtf2h5	536.5553827	-3.345445873	1.14314	-2.92654	0.003428	0.041597
Nfe2l2	3535.582607	2.811745843	0.960855	2.926295	0.00343	0.041597
Ddx19a	40027.18112	2.376167279	0.813189	2.922036	0.003478	0.042109
Srp54a	254.4577656	-4.684261815	1.604597	-2.91928	0.003508	0.042421
Mbd4	320.8673164	-4.29992385	1.473234	-2.9187	0.003515	0.042437
Ift22	1334.229623	-2.796212178	0.960008	-2.9127	0.003583	0.043198
Pabpn1l	2213.093994	-2.633191877	0.904459	-2.91135	0.003599	0.04326
Dnajc11	1673.784017	-2.699848448	0.927358	-2.91133	0.003599	0.04326
Klhl32	667.1799709	-3.161635933	1.087442	-2.90741	0.003644	0.043743
Cnnm2	1249.578798	2.803962138	0.965692	2.903578	0.003689	0.044217
Eefsec	616.6396303	-3.844476871	1.324367	-2.90288	0.003697	0.044252
Prss37	327.3920898	-4.10104591	1.414409	-2.89948	0.003738	0.044641
Cdk6	144.3303935	-5.335520044	1.840392	-2.89912	0.003742	0.044641
Hmgn5	3067.076434	2.532104781	0.87351	2.898771	0.003746	0.044641
Ddx56	222.4102517	-4.485743326	1.549547	-2.89487	0.003793	0.045133
Slc16a12	364.0632384	3.732686787	1.289777	2.894056	0.003803	0.045186
AU015836	207810.5124	2.363791974	0.818593	2.887629	0.003882	0.045988
Smco2	178.4965703	-5.645642758	1.955117	-2.88762	0.003882	0.045988
Fkbp14	487.935312	3.901290187	1.351415	2.886819	0.003892	0.046039
Zfp956	984.7290283	-3.444049379	1.193436	-2.88583	0.003904	0.046091
Eri3	507.9526619	-3.586285227	1.242838	-2.88556	0.003907	0.046091
Carmil2	533.1622606	3.27311369	1.134588	2.884848	0.003916	0.04613
Manf	233.874671	-5.223262184	1.811034	-2.88413	0.003925	0.046169
Cyp2b23	230.5546807	-4.292238386	1.490596	-2.87955	0.003982	0.046772
Limk1	531.0412975	-3.620555917	1.257512	-2.87914	0.003988	0.046772
Pycr1	1076.758278	3.439821548	1.195409	2.877527	0.004008	0.046792
Pygo1	962.0263816	-2.932392501	1.019081	-2.87749	0.004009	0.046792

Xpo5	2008.355558	2.656971811	0.923397	2.877388	0.00401	0.046792
Trmu	177.934706	-4.82253459	1.676114	-2.87721	0.004012	0.046792
AU040320	719.305279	-3.096673663	1.077321	-2.87442	0.004048	0.047141
Lrrc41	386.0813021	-3.584612047	1.24834	-2.8715	0.004085	0.047462
Ciapin1	1635.083836	-2.680795195	0.933625	-2.87138	0.004087	0.047462
Map3k10	527.3788762	-4.230731319	1.473847	-2.87054	0.004098	0.047477
Zfp735	11818.35234	2.422798315	0.844065	2.870393	0.0041	0.047477
Wwp2	667.9387787	-3.24130092	1.129708	-2.86915	0.004116	0.047597
Setdb2	412.0478612	-3.598994569	1.254926	-2.86789	0.004132	0.04772
Nrip1	2420.938314	3.12435348	1.090319	2.86554	0.004163	0.048008
Aurkaip1	529.4898063	-3.27313373	1.14624	-2.85554	0.004296	0.049451
Ccdc85c	5056.095068	-2.874990527	1.006907	-2.85527	0.0043	0.049451
Smpd1	2389.343815	-2.54583254	0.892376	-2.85287	0.004333	0.049637
Fam206a	2285.016349	2.562578392	0.898322	2.852627	0.004336	0.049637
Gm340	273.5208213	-3.982707469	1.396243	-2.85245	0.004338	0.049637
Zpr1	1099.769395	2.800185807	0.981728	2.852302	0.00434	0.049637
Txndc11	2419.881037	-2.584146772	0.906398	-2.85101	0.004358	0.04977
