# Supplementary material for: The RNA-binding protein DAZL functions as repressor and activator of mRNA translation during oocyte maturation
Source: Nat Commun. 2020 Mar 13;11:1399. doi: 10.1038/s41467-020-15209-9 (PMC7070028; doi:10.1038/s41467-020-15209-9)
Supplement: Supplementary file 4 — Supplementary Data 2 [file 41467_2020_15209_MOESM4_ESM.pdf]

probe set	gene	Accession	Ab/IgG	P value
10471072	Tor1b: torsin family 1, member B	NM_133673	2.108065318	4.97813E-06
10454469	Ammecr1l: AMME chromosomal region gene 1-like	NM_153515	1.52884762	6.19826E-05
10508770	Gm13033: prostaglandin-endoperoxide synthase 2 pseudogene	AK142202	1.642683608	8.40924E-05
10545524	4931417E11Rik: RIKEN cDNA 4931417E11 gene	BC048456	1.512632486	0.000151504
10569011	Ifitm5: interferon induced transmembrane protein 5	NM_053088	1.700227613	0.000167583
10428211	Zfp706: zinc finger protein 706	NM_026521	2.24143774	0.000187763
10415784	Trim13: tripartite motif-containing 13	NM_001164220	2.112437322	0.000201784
10422518	Tmtc4: transmembrane and tetratricopeptide repeat containing 4	NM_028651	2.086653045	0.000228858
10584124	Arhgap32: Rho GTPase activating protein 32	NM_177379	1.53256733	0.000230605
10367673	Plekhg1: pleckstrin homology domain containing, family G (with RhoG	NM_001033253	1.833878221	0.00026236
10374500	Vps54: vacuolar protein sorting 54 (yeast)	NM_139061	2.188027378	0.000347876
10440099	St3gal6: ST3 beta-galactoside alpha-2,3-sialyltransferase 6	NM_018784	1.991791166	0.00036693
10376685	Alkbh5: alkB, alkylation repair homolog 5 (E. coli)	NM_172943	1.526370677	0.000378759
10600377	Atp6ap1: ATPase, H+ transporting, lysosomal accessory protein 1	NM_018794	1.539651715	0.000393188
10542108	Tom1: target of myb1 homolog (chicken)	NM_011622	2.099725944	0.000417052
10603362	Ccdc120: coiled-coil domain containing 120	NM_207202	1.553199067	0.000452869
10573803	Cyld: cylindromatosis (turban tumor syndrome)	NM_001128169	1.586143951	0.00050062
10591164	Zfp558: zinc finger protein 558	NM_028935	2.2384443	0.000517256
10527713	Rxfp2: relaxin/insulin-like family peptide receptor 2	NM_080468	1.892546101	0.000543274
10368981	Lin28b: lin-28 homolog B (C. elegans)	NM_001031772	1.722991396	0.000611455
10447224	Dync2li1: dynein cytoplasmic 2 light intermediate chain 1	NM_172256	1.602660976	0.000646601
10457918	Gm5064: predicted gene 5064	XR_031138	1.647811537	0.000688464
10357630	Srgap2: SLIT-ROBO Rho GTPase activating protein 2	NM_001081011	1.984622967	0.000711682
10567423	Dcun1d3: DCN1, defective in cullin neddylation 1, domain containing 3	NM_173408	1.589198348	0.000715749
10484648	Olfr1104: olfactory receptor 1104	NM_146767	1.790784247	0.000777442
10515242	Nsun4: NOL1/NOP2/Sun domain family, member 4	NM_028142	1.785225582	0.000807404
10422760	Fyb: FYN binding protein	NM_011815	1.630780028	0.000884325
10501104	Slc6a17: solute carrier family 6 (neurotransmitter transporter), membe	NM_172271	1.955850571	0.000925787
10604038	Sfrs17b: splicing factor, arginine/serine-rich 17b	NM_001081956	1.930616252	0.000997379
10373778	Morc2a: microrchidia 2A	NM_001159288	1.763401987	0.001198559
10540241	Arl6ip5: ADP-ribosylation factor-like 6 interacting protein 5	NM_022992	1.534792671	0.001282616
10350753	Glul: glutamate-ammonia ligase (glutamine synthetase)	NM_008131	1.640934405	0.001309735
10357124	Tsn: translin	NM_011650	2.125699481	0.001316266
10571984	Ddx60: DEAD (Asp-Glu-Ala-Asp) box polypeptide 60	NM_001081215	1.592800872	0.001327755
10538927	Amd1: S-adenosylmethionine decarboxylase 1	NM_009665	1.526723851	0.001344636
10512226	Dcaf12: DDB1 and CUL4 associated factor 12	NM_026893	1.589015033	0.001359695
10479752	Olah: oleoyl-ACP hydrolase	NM_145921	1.789585925	0.001430493
10369752	Lrrtm3: leucine rich repeat transmembrane neuronal 3	NM_178678	1.732075472	0.001523116
10530089	Cckar: cholecystokinin A receptor	NM_009827	1.554893785	0.001563321
10490078	Sumo1: SMT3 suppressor of mif two 3 homolog 1 (yeast)	NM_009460	2.004066563	0.001580727
10563722	Mrgpra3: MAS-related GPR, member A3	NM_153067	1.767819026	0.001597371
10502355	Gm4861: predicted gene 4861	NM_177665	1.64717879	0.001598877
10497250	Pag1: phosphoprotein associated with glycosphingolipid microdomain	BC145761	1.814009505	0.001631502
10369890	Cisd1: CDGSH iron sulfur domain 1	BC013522	1.567545897	0.001640222
10537934	Zfp212: Zinc finger protein 212	NM_145576	2.213405797	0.001728864
10580082	Rfx1: regulatory factor X, 1 (influences HLA class II expression)	NM_009055	1.881306983	0.001752752
10546056	Rab43: RAB43, member RAS oncogene family	NM_001039394	1.869826488	0.001765379
10408487	Uqcrfs1: ubiquinol-cytochrome c reductase, Rieske iron-sulfur polypep	NM_025710	2.16726	0.001776114
10416956	Mir19b-1: microRNA 19b-1	NR_029815	1.757267442	0.001799081
10422628	Plcxd3: phosphatidylinositol-specific phospholipase C, X domain conta	NM_177355	1.833794557	0.001828254
10372618	Frs2: fibroblast growth factor receptor substrate 2	NM_177798	1.62630937	0.001884104
10543226	2610001J05Rik: RIKEN cDNA 2610001J05 gene	NR_024619	1.506547641	0.001923556
10404578	Cdyl: chromodomain protein, Y chromosome-like	NM_009881	1.562197924	0.001925785
10356172	Pid1: phosphotyrosine interaction domain containing 1	NM_001003948	1.824939428	0.001940007
10459389	Amd1: S-adenosylmethionine decarboxylase 1	NM_009665	1.527614372	0.001994837
10415608	Ift88: intraflagellar transport 88 homolog (Chlamydomonas)	NM_009376	1.701922635	0.002238547
10518805	Vamp3: vesicle-associated membrane protein 3	NM_009498	2.173909813	0.002286511
10427849	6030458C11Rik: RIKEN cDNA 6030458C11 gene	NM_029998	1.513496965	0.00228704
10458424	Taf7: TAF7 RNA polymerase II, TATA box binding protein (TBP)-associa	NM_175770	2.012294038	0.002339944
10526487	Alkbh4: alkB, alkylation repair homolog 4 (E. coli)	NM_028070	1.526144584	0.002384788
10464479	Saps3: SAPS domain family, member 3	NM_028999	1.893369808	0.002396355
10400795	Sav1: salvador homolog 1 (Drosophila)	NM_022028	1.894190643	0.002404217
10503023	Cth: cystathionase (cystathionine gamma-lyase)	NM_145953	1.581623961	0.002434731
10357952	Ppp1r12b: protein phosphatase 1, regulatory (inhibitor) subunit 12B	NM_001081307	1.848843295	0.002517102

10560015	Rnf141: ring finger protein 141	NM_025999	1.883988996	0.002517951
10586933	Nedd4: neural precursor cell expressed, developmentally down-regula	NM_010890	1.777419994	0.00264115
10457872	Slc39a6: solute carrier family 39 (metal ion transporter), member 6	NM_139143	1.955373319	0.002654611
10484773	Olfr1214: olfactory receptor 1214	NM_146897	1.793109059	0.002676724
10396862	Actn1: actinin, alpha 1	NM_134156	1.518101628	0.00282699
10557754	Orai3: ORAI calcium release-activated calcium modulator 3	NM_198424	1.807764233	0.002850344
10428534	Trps1: trichorhinophalangeal syndrome I (human)	NM_032000	2.123986454	0.002892178
10575120	Sntb2: syntrophin, basic 2	NM_009229	1.973889603	0.003072352
10492720	Mtap9: microtubule-associated protein 9	NM_001081230	1.770448117	0.003097439
10490955	Mtfr1: mitochondrial fission regulator 1	NM_026182	1.771411351	0.0031542
10476033	Stk35: serine/threonine kinase 35	NM_183262	1.999150806	0.003239278
10453026	Prkd3: protein kinase D3	NM_001171004	1.852598287	0.003242785
10446656	Lpin2: lipin 2	NM_001164885	1.727475877	0.003333779
10374453	Glul: glutamate-ammonia ligase (glutamine synthetase)	NM_008131	1.641489046	0.003373734
10607747	Siah1b: seven in absentia 1B	NM_009173	1.937746918	0.00339946
10522250	Tmem33: transmembrane protein 33	NM_028975	2.066338601	0.003414258
10388591	Cpd: carboxypeptidase D	NM_007754	1.573678129	0.003417887
10584162	Tmed2: transmembrane emp24 domain trafficking protein 2	NM_019770	2.035264142	0.003530727
10353320	Crisp4: cysteine-rich secretory protein 4	NM_030033	1.576520921	0.003539522
10556463	Arntl: aryl hydrocarbon receptor nuclear translocator-like	NM_007489	1.535301178	0.003574932
10585494	Ube2q2: ubiquitin-conjugating enzyme E2Q (putative) 2	NM_180600	2.156659196	0.003608488
10428933	H2afy3: H2A histone family, member Y3	NR_003523	1.674247879	0.003619667
10413596	Sfmbt1: Scm-like with four mbt domains 1	NM_001166532	1.928972577	0.003751201
10583242	Sesn3: sestrin 3	NM_030261	1.922004831	0.003760121
10411690	Rad17: RAD17 homolog (S. pombe)	NM_011233	1.872200403	0.003801179
10587023	Rab27a: RAB27A, member RAS oncogene family	NM_023635	1.863286089	0.003831363
10517159	Lin28a: lin-28 homolog A (C. elegans)	NM_145833	1.722455037	0.003877865
10515943	Ctps: cytidine 5'-triphosphate synthase	NM_016748	1.582246914	0.00393068
10468519	Smndc1: survival motor neuron domain containing 1	NM_172429	1.970126466	0.003975558
10603746	Maob: monoamine oxidase B	NM_172778	1.740468591	0.003980338
10425114	Tmed2: transmembrane emp24 domain trafficking protein 2	NM_019770	2.036967844	0.004020747
10509113	Sfrs13a: splicing factor, arginine/serine-rich 13A	NM_010178	1.930220075	0.004065816
10417253	Gm1973: predicted gene 1973	NM_029288	1.644290494	0.004123254
10604405	Aifm1: apoptosis-inducing factor, mitochondrion-associated 1	NM_012019	1.52261571	0.004123819
10453544	Mettl4: methyltransferase like 4	NM_176917	1.747395112	0.004146054
10497878	Il2: interleukin 2	NM_008366	1.703261516	0.004167378
10606619	Sytl4: synaptotagmin-like 4	NM_013757	2.012096455	0.00417194
10540911	Tsen2: tRNA splicing endonuclease 2 homolog (S. cerevisiae)	NM_199033	2.124958402	0.004236993
10523670	Aff1: AF4/FMR2 family, member 1	NM_001080798	1.520392808	0.004287144
10484581	Olfr1053: olfactory receptor 1053	NM_001177857	1.790327048	0.004298499
10599537	Slc25a14: solute carrier family 25 (mitochondrial carrier, brain), memb	NM_011398	1.94242597	0.004413129
10444394	Pbx2: pre B-cell leukemia transcription factor 2	NM_017463	1.814415144	0.004447655
10425801	Bik: BCL2-interacting killer	NM_007546	1.542498568	0.004458078
10407513	Wdr37: WD repeat domain 37	NM_172445	2.191631287	0.004536476
10486499	Tmem87a: transmembrane protein 87A	NM_173734	2.079287725	0.004554368
10414590	Ear6: eosinophil-associated, ribonuclease A family, member 6	NM_053111	1.603689691	0.004628747
10525744	Tmed2: transmembrane emp24 domain trafficking protein 2	NM_019770	2.034752414	0.004738225
10607910	Msl3: male-specific lethal 3 homolog (Drosophila)	NM_010832	1.769872064	0.004748363
10516884	Trnau1ap: tRNA selenocysteine 1 associated protein 1	NM_027925	2.121664268	0.00479644
10461012	Trmt112: tRNA methyltransferase 11-2 homolog (S. cerevisiae)	NM_001166370	2.121664268	0.00479644
10511062	Tmem52: transmembrane protein 52	NM_027161	2.072819034	0.004874711
10559238	Igf2as: insulin-like growth factor 2, antisense	NR_002855	1.702017793	0.004893888
10551355	Sertad3: SERTA domain containing 3	NM_133210	1.921252322	0.004973209
10596988	Plxnb1: plexin B1	NM_172775	1.836815632	0.004977343
10363563	Slc25a16: solute carrier family 25 (mitochondrial carrier, Graves diseas	NM_175194	1.943492568	0.005035462
10479297	Lsm14b: LSM14 homolog B (SCD6, S. cerevisiae)	NM_177727	1.733120827	0.005045725
10431154	Phf21b: PHD finger protein 21B	NR_030731	1.82126117	0.005067706
10569085	Lrrc56: leucine rich repeat containing 56	NM_001172064	1.731522444	0.005190518
10512030	3110043O21Rik: RIKEN cDNA 3110043O21 gene	BC076612	1.508960573	0.005236059
10484884	Olfr1272: olfactory receptor 1272	NM_146980	1.79443371	0.005263072
10507273	Pik3r3: phosphatidylinositol 3 kinase, regulatory subunit, polypeptide :	NM_181585	1.82685328	0.005385125
10496629	Sep15: selenoprotein	NM_053102	1.913669138	0.005387189
10498919	Gm9989: predicted gene 9989	ENSMUST00000069	1.66129204	0.005390323
10355017	Sumo1: SMT3 suppressor of mif two 3 homolog 1 (yeast)	NM_009460	2.00258653	0.005463216
10459611	Mc2r: melanocortin 2 receptor	NM_008560	1.746437531	0.005466741

10407145	Mier3: mesoderm induction early response 1, family member 3	NM_172593	1.750968175	0.005507928
10466888	Glis3: GLIS family zinc finger 3	NM_175459	1.638783346	0.005520381
10407209	Slc38a9: solute carrier family 38, member 9	NM_178746	1.952990751	0.005529344
10397912	Unc79: unc-79 homolog (C. elegans)	NM_001081017	2.164472481	0.00553044
10598638	Mid1ip1: Mid1 interacting protein 1 (gastrulation specific G12-like (zel	NM_001166635	1.750270233	0.005540502
10419073	Tspan14: tetraspanin 14	NM_145928	2.134047906	0.005568254
10606195	Rlim: ring finger protein, LIM domain interacting	NM_011276	1.882379336	0.005574826
10530558	Slain2: SLAIN motif family, member 2	NM_153567	1.940781234	0.005576518
10412755	Gm8471: predicted gene 8471	XR_033275	1.65766366	0.005605346
10466282	Olfr1426: olfactory receptor 1426	NM_146809	1.799223702	0.005612489
10447490	Pja2: praja 2, RING-H2 motif containing	NM_001025309	1.830509138	0.005626048
10514201	Haus6: HAUS augmin-like complex, subunit 6	NM_173400	1.676976182	0.005844097
10567412	Eri2: exoribonuclease 2	NM_027698	1.608801466	0.005885808
10530045	Sepsecs: Sep (O-phosphoserine) tRNA:Sec (selenocysteine) tRNA synth	NM_172490	1.914152305	0.005933954
10599863	4931400O07Rik: RIKEN cDNA 4931400O07 gene	ENSMUST00000033	1.511634128	0.005950623
10547191	Tmem72: transmembrane protein 72	NM_178768	2.076858093	0.00610749
10502961	Lhx8: LIM homeobox protein 8	NM_010713	1.719577874	0.006121694
10420316	Atp12a: ATPase, H+/K+ transporting, nongastric, alpha polypeptide	NM_138652	1.539222205	0.006145077
10430711	Slc25a17: solute carrier family 25 (mitochondrial carrier, peroxisomal r	NM_011399	1.945112157	0.00615871
10593526	Atm: ataxia telangiectasia mutated homolog (human)	NM_007499	1.538633888	0.006183976
10405400	Nsd1: nuclear receptor-binding SET-domain protein 1	NM_008739	1.782869665	0.006289772
10555407	Chchd8: coiled-coil-helix-coiled-coil-helix domain containing 8	NM_183270	1.565972412	0.006292596
10433352	Ubn1: ubinuclein 1	NM_026666	2.162316093	0.006351065
10440993	Rcan1: regulator of calcineurin 1	NM_001081549	1.875106712	0.006491726
10458033	Stard4: StAR-related lipid transfer (START) domain containing 4	NM_133774	1.996890404	0.006554782
10351099	Tnfsf18: tumor necrosis factor (ligand) superfamily, member 18	NM_183391	2.099247649	0.006573962
10439835	Gm4802: predicted gene 4802	XM_126432	1.647070305	0.006597274
10400515	Sec23a: SEC23A (S. cerevisiae)	NM_009147	1.903091312	0.006613731
10557139	Ubfd1: ubiquitin family domain containing 1	NM_138589	2.159353736	0.006695573
10408142	Vmn1r206: vomeronasal 1 receptor 206	NM_134216	2.176046226	0.006768379
10449935	Zfp870: zinc finger protein 870	NM_207245	2.245957103	0.006913114
10549497	Fam60a: family with sequence similarity 60, member A	NM_019643	1.620012868	0.006998401
10585572	Hmg20a: high mobility group 20A	NM_025812	1.688606625	0.007086861
10459705	Smad4: MAD homolog 4 (Drosophila)	NM_008540	1.963295106	0.007096098
10503150	Rab2a: RAB2A, member RAS oncogene family	NM_021518	1.866000725	0.007111821
10458583	Yipf5: Yip1 domain family, member 5	NM_023311	2.20018406	0.007181241
10563314	Dhdh: dihydrodiol dehydrogenase (dimeric)	NM_027903	1.59446048	0.0072074
10533989	LOC639341: similar to Spindlin-like protein 2 (SPIN-2)	XM_915736	1.724796898	0.007226869
10599192	Lonrf3: LON peptidase N-terminal domain and ring finger 3	NM_028894	1.726215026	0.007251791
10501661	Sfrs3: splicing factor, arginine/serine-rich 3 (SRp20)	BC083316	1.933720034	0.007252792
10476759	Rin2: Ras and Rab interactor 2	NM_028724	1.881962852	0.007283302
10443940	Zfp422-rs1: zinc finger protein 422, related sequence 1	NM_029952	2.230105839	0.007318316
10529581	Mrfap1: Morf4 family associated protein 1	NM_026242	1.76519359	0.00732324
10584416	Olfr905: olfactory receptor 905	NM_146804	1.805828428	0.007378412
10385479	Ibtk: inhibitor of Bruton agammaglobulinemia tyrosine kinase	NM_001081282	1.69827204	0.007490054
10433077	Smug1: single-strand selective monofunctional uracil DNA glycosylase	NM_027885	1.970212205	0.007584985
10363860	Slc16a9: solute carrier family 16 (monocarboxylic acid transporters), m	NM_025807	1.942260477	0.007609024
10390153	Myst2: MYST histone acetyltransferase 2	NM_177619	1.775266116	0.007642333
10414355	Mapk1ip1l: mitogen-activated protein kinase 1 interacting protein 1-lil	NM_178684	1.743246471	0.007657724
10413897	Ercc6: excision repair cross-complementing rodent repair deficiency, c	NM_001081221	1.608604134	0.007704813
10453166	Cdkl4: cyclin-dependent kinase-like 4	NM_001033443	1.561846496	0.007717696
10546134	Kbtbd12: kelch repeat and BTB (POZ) domain containing 12	NM_029013	1.706613542	0.007804527
10487945	Gpcpd1: glycerophosphocholine phosphodiesterase GDE1 homolog (S.	NM_028802	1.666119183	0.007810965
10368486	Rnf146: ring finger protein 146	NM_001110197	1.885320263	0.007826514
10457385	Ccny: cyclin Y	NM_026484	1.556449276	0.007833393
10606217	Abcb7: ATP-binding cassette, sub-family B (MDR/TAP), member 7	NM_009592	1.51594868	0.007910038
10349793	Dstyk: dual serine/threonine and tyrosine protein kinase	NM_172516	1.598978476	0.007923582
10446581	Zfp161: zinc finger protein 161	NM_009547	2.212787814	0.007977156
10458767	Trim36: tripartite motif-containing 36	NM_178872	2.115854686	0.008017732
10536595	Naa38: N(alpha)-acetyltransferase 38, NatC auxiliary subunit	NM_133939	1.775414215	0.008042891
10549794	Gm5065: predicted gene 5065	NR_003622	1.648089138	0.008059064
10412454	Ctsm: cathepsin M	NM_022326	1.58280343	0.008069265
10491231	Mynn: myoneurin	NM_030557	1.773642448	0.008358382
10354258	Uxs1: UDP-glucuronate decarboxylase 1	NM_026430	2.168486299	0.008440438
10475912	Tmem87b: transmembrane protein 87B	NM_028248	2.085231089	0.008540489

10424126	Depdc6: DEP domain containing 6	NM_001037937	1.59328638	0.008566478
10521589	Cir1: corepressor interacting with RBPJ, 1	NM_025854	1.566867761	0.008766063
10441330	Zfp295: zinc finger protein 295	NM_175428	2.22202228	0.008777114
10428020	March6: membrane-associated ring finger (C3HC4) 6	NM_172606	1.745737866	0.008858686
10399841	Cbll1: Casitas B-lineage lymphoma-like 1	NM_134048	1.551969915	0.008975319
10369655	Stox1: storkhead box 1	NM_001033260	2.001678783	0.009072277
10539813	Ccdc48: coiled-coil domain containing 48	NM_001159697	1.554163076	0.009150711
10398678	Eif5: eukaryotic translation initiation factor 5	NM_173363	1.605709845	0.0091529
10492582	Mir15b: microRNA 15b	NR_029529	1.753880495	0.009217065
10554839	Picalm: phosphatidylinositol binding clathrin assembly protein	NM_146194	1.823435395	0.009222789
10502079	5730508B09Rik: RIKEN cDNA 5730508B09 gene	BC116791	1.51339091	0.009295684
10425357	Smcr7l: Smith-Magenis syndrome chromosome region, candidate 7-lik	NM_178719	1.964048029	0.00933654
10545623	Dok1: docking protein 1	NM_010070	1.597779548	0.009353169
10505451	Orm2: orosomucoid 2	NM_011016	1.808720398	0.009382233
10584138	Kcnj1: potassium inwardly-rectifying channel, subfamily J, member 1	NM_001168354	1.708200814	0.009413332
10484237	Zfp385b: zinc finger protein 385B	NM_178723	2.226669428	0.009449778
10369844	Bicc1: bicaudal C homolog 1 (Drosophila)	NM_031397	1.542323774	0.009477202
10447356	Socs5: suppressor of cytokine signaling 5	NM_019654	1.976728199	0.009581045
10566686	Olfr490: olfactory receptor 490	NM_146498	1.802946651	0.009611375
10437765	Cpped1: calcineurin-like phosphoesterase domain containing 1	NM_146067	1.575040169	0.009770667
10571142	Gpr124: G protein-coupled receptor 124	NM_054044	1.66705892	0.009781979
10467068	Sgms1: sphingomyelin synthase 1	NM_001168525	1.934158007	0.009902849
10593159	Pafah1b2: platelet-activating factor acetylhydrolase, isoform 1b, subui	NM_008775	1.811506106	0.009912039
10504422	Olfr155: olfactory receptor 155	NM_019473	1.800982997	0.010019166
10400967	Six1: sine oculis-related homeobox 1 homolog (Drosophila)	NM_009189	1.93995644	0.01012216
10407833	Ggps1: geranylgeranyl diphosphate synthase 1	NM_010282	1.636788304	0.01014866
10463227	Gm6937: predicted pseudogene 6937	BC089359	1.654519671	0.010278967
10457644	Cdh2: cadherin 2	NM_007664	1.561116401	0.010340929
10460371	Ptprcap: protein tyrosine phosphatase, receptor type, C polypeptide-a	NM_016933	1.857160886	0.010504522
10604958	Magea10: melanoma antigen family A, 10	NM_001085506	1.737466986	0.010600388
10369116	Mcm9: minichromosome maintenance complex component 9	NM_027830	1.746749376	0.010612798
10473622	Olfr1255: olfactory receptor 1255	NM_146977	1.794382914	0.010672869
10570573	Agpat5: 1-acylglycerol-3-phosphate O-acyltransferase 5 (lysophosphat	NM_026792	1.520519077	0.010684195
10498500	Vmn2r1: vomeronasal 2, receptor 1	NM_019918	2.179607176	0.010826711
10508454	Bsdc1: BSD domain containing 1	NM_133889	1.543654647	0.01084101
10433088	Cbx5: chromobox homolog 5 (Drosophila HP1a)	NM_007626	1.552092439	0.010876249
10437942	Ube2v2: ubiquitin-conjugating enzyme E2 variant 2	NM_023585	2.157478897	0.010880205
10368670	Amd1: S-adenosylmethionine decarboxylase 1	NM_009665	1.527240895	0.010948528
10492846	Pet112l: PET112-like (yeast)	NM_144896	1.819084193	0.010969512
10585428	Dnaja4: DnaJ (Hsp40) homolog, subfamily A, member 4	NM_021422	1.595720474	0.010982673
10403248	Speer6-ps1: spermatogenesis associated glutamate (E)-rich protein 6,	NR_001581	1.981896357	0.011127812
10566417	Olfr675: olfactory receptor 675	NM_001011848	1.805487748	0.011167746
10422854	Nup155: nucleoporin 155	NM_133227	1.788359	0.011182181
10532741	Tmem119: transmembrane protein 119	NM_146162	2.037529995	0.011198259
10409905	Ctsr: cathepsin R	NM_020284	1.583988772	0.011217643
10470296	Mir126: microRNA 126	NR_029541	1.752866291	0.011244119
10592515	Ubash3b: ubiquitin associated and SH3 domain containing, B	NM_176860	2.143771842	0.011287365
10354868	Fam126b: family with sequence similarity 126, member B	NM_172513	1.615819366	0.011308605
10522024	Tbc1d1: TBC1 domain family, member 1	NM_019636	2.013598681	0.011345015
10514985	Zyg11b: zyg-ll homolog B (C. elegans)	NM_001033634	2.254898187	0.011445906
10455813	Lmnb1: lamin B1	NM_010721	1.723828556	0.011473942
10382284	Prkar1a: protein kinase, cAMP dependent regulatory, type I, alpha	NM_021880	1.850193058	0.011494232
10458555	Spry4: sprouty homolog 4 (Drosophila)	NM_011898	1.983547926	0.01150069
10532275	Smndc1: survival motor neuron domain containing 1	NM_172429	1.969377829	0.011525925
10468311	Sh3pxd2a: SH3 and PX domains 2A	NM_008018	1.934300486	0.011552877
10422244	Slitrk6: SLIT and NTRK-like family, member 6	NM_175499	1.962999131	0.011565332
10576661	Itgb1: integrin beta 1 (fibronectin receptor beta)	NM_010578	1.706264474	0.011580218
10608138	Ddx3y: DEAD (Asp-Glu-Ala-Asp) box polypeptide 3, Y-linked	NM_012008	1.59278544	0.011581449
10421685	Serp2: stress-associated endoplasmic reticulum protein family membe	NR_027699	1.919281971	0.011621316
10603109	Piga: phosphatidylinositol glycan anchor biosynthesis, class A	NM_011081	1.825008094	0.011707907
10436442	Fam60a: family with sequence similarity 60, member A	NM_019643	1.621421021	0.011745126
10480878	Camsap1: calmodulin regulated spectrin-associated protein 1	NM_001115076	1.550484815	0.011861566
10558285	Zranb1: zinc finger, RAN-binding domain containing 1	NM_207302	2.250266972	0.011893862
10346298	Coq10b: coenzyme Q10 homolog B (S. cerevisiae)	NM_001039710	1.571426875	0.011907355
10548667	Tas2r136: taste receptor, type 2, member 136	NM_181276	2.013557421	0.011927845

10476886	Sstr4: somatostatin receptor 4	NM_009219	1.988686585	0.012036438
10417492	Gm5458: predicted gene 5458	NM_001024706	1.64979197	0.012054832
10487564	Zc3h8: zinc finger CCCH type containing 8	NM_020594	2.208878444	0.012179519
10591517	Cdkn2d: cyclin-dependent kinase inhibitor 2D (p19, inhibits CDK4)	NM_009878	1.562025554	0.012246702
10485294	Hsd17b12: hydroxysteroid (17-beta) dehydrogenase 12	NM_019657	1.696141282	0.012247913
10399198	Ncoa4: nuclear receptor coactivator 4	NM_019744	1.77637106	0.012356564
10368748	Amd1: S-adenosylmethionine decarboxylase 1	NM_009665	1.527275001	0.012367583
10545038	Vmn1r30: vomeronasal 1 receptor 30	NM_134177	2.179317351	0.012377641
10498350	P2ry14: purinergic receptor P2Y, G-protein coupled, 14	NM_133200	1.810872742	0.012386829
10445953	Emr4: EGF-like module containing, mucin-like, hormone receptor-like :	NM_139138	1.607531272	0.012428018
10388290	Olfr393: olfactory receptor 393	NM_147008	1.802264843	0.012483154
10565330	Zfand6: zinc finger, AN1-type domain 6	NM_022985	2.210388428	0.012545544
10439208	Sec22a: SEC22 vesicle trafficking protein homologe A (S. cerevisiae)	NM_133704	1.897631514	0.012643187
10476252	Cdc25b: cell division cycle 25 homolog B (S. pombe)	NM_023117	1.558379841	0.012677379
10606376	2610002M06Rik: RIKEN cDNA 2610002M06 gene	NM_025921	1.507189965	0.012705365
10540265	Mitf: microphthalmia-associated transcription factor	NM_001113198	1.760758934	0.012900883
10359961	Uhmk1: U2AF homology motif (UHM) kinase 1	NM_010633	2.163886188	0.012918959
10541910	Vwf: Von Willebrand factor homolog	NM_011708	2.191051498	0.013059253
10345777	Il1rl2: interleukin 1 receptor-like 2	NM_133193	1.70315856	0.01310813
10522712	Rest: RE1-silencing transcription factor	NM_011263	1.878161404	0.013108396
10506843	Cc2d1b: coiled-coil and C2 domain containing 1B	NM_177045	1.552427303	0.013190593
10541112	Hnrnpf: heterogeneous nuclear ribonucleoprotein F	BC018185	1.694469208	0.013313643
10528880	Lmbr1: limb region 1	NM_020295	1.723088937	0.01331607
10440669	2310057N15Rik: RIKEN cDNA 2310057N15 gene	BC104341	1.506472888	0.013441187
10539773	Gfpt1: glutamine fructose-6-phosphate transaminase 1	NM_013528	1.635469725	0.013476442
10498907	Glrb: glycine receptor, beta subunit	NM_010298	1.638979217	0.013518524
10396125	Atl1: atlastin GTPase 1	NM_178628	1.538378029	0.013680089
10442292	Vmn2r113: vomeronasal 2, receptor 113	NM_001104578	2.18286518	0.013725773
10542650	Golt1b: golgi transport 1 homolog B (S. cerevisiae)	NM_025872	1.665482816	0.01378129
10582123	Hsdl1: hydroxysteroid dehydrogenase like 1	NM_175185	1.696460997	0.013969971
10380109	Hsf5: heat shock transcription factor family member 5	NM_001045527	1.696460997	0.013969971
10360957	Kctd3: potassium channel tetramerisation domain containing 3	NM_172650	1.708887222	0.013989399
10589929	Cmtm6: CKLF-like MARVEL transmembrane domain containing 6	NM_026036	1.569849634	0.014046873
10354432	Myo1b: myosin IB	NM_001161817	1.773993076	0.014196542
10422946	Ranbp3l: RAN binding protein 3-like	NM_198024	1.873132737	0.014316384
10500610	Fam46c: family with sequence similarity 46, member C	NM_001142952	1.618537257	0.014391198
10352092	Zfp238: zinc finger protein 238	NM_001012330	2.215959574	0.014699295
10467191	Ankrd1: ankyrin repeat domain 1 (cardiac muscle)	NM_013468	1.529780829	0.014717826
10528360	Slc26a5: solute carrier family 26, member 5	NM_030727	1.946724837	0.014723758
10594840	Gcom1: GRINL1A complex locus	NM_001033208	1.632355437	0.01474354
10531323	G3bp2: GTPase activating protein (SH3 domain) binding protein 2	NM_011816	1.630921453	0.014780202
10585347	4930550C14Rik: RIKEN cDNA 4930550C14 gene	BC115639	1.510557573	0.014800849
10497203	Hey1: hairy/enhancer-of-split related with YRPW motif 1	NM_010423	1.682261124	0.01482799
10355227	1110028C15Rik: RIKEN cDNA 1110028C15 gene	NM_001122738	1.501794597	0.014858539
10404836	Rnf182: ring finger protein 182	NM_183204	1.885634646	0.014863741
10598164	0610010K06Rik: RIKEN cDNA 0610010K06 gene	NM_027861	1.501663874	0.014871476
10457040	Zfp516: zinc finger protein 516	NM_183033	2.231070091	0.015012592
10403413	Idi1: isopentenyl-diphosphate delta isomerase	NM_145360	1.698975663	0.015020929
10562117	Ffar2: free fatty acid receptor 2	NM_146187	1.624678663	0.015453179
10558992	Muc2: mucin 2	NM_023566	1.772849946	0.015608792
10351644	Cd244: CD244 natural killer cell receptor 2B4	NM_018729	1.55722951	0.015671438
10528200	Hnrnpa3: heterogeneous nuclear ribonucleoprotein A3	NM_053263	1.689174485	0.015716672
10538617	Lancl2: LanC (bacterial lantibiotic synthetase component C)-like 2	NM_133737	1.718154278	0.015734923
10445796	AI314976: expressed sequence AI314976	BC022574	1.521286843	0.015875488
10569998	Gm5605: predicted gene 5605	XR_031660	1.650847085	0.015952008
10481337	1700101E01Rik: RIKEN cDNA 1700101E01 gene	NM_001166705	1.503903259	0.015991706
10489891	B4galt5: UDP-Gal:betaGlcNAc beta 1,4-galactosyltransferase, polypept	NM_019835	1.541253301	0.015995113
10449920	Zfp811: zinc finger protein 811	NM_183177	2.244304936	0.016053826
10502951	Acadm: acyl-Coenzyme A dehydrogenase, medium chain	NM_007382	1.517112712	0.016055045
10420675	Gucy1b2: guanylate cyclase 1, soluble, beta 2	NM_172810	1.672730063	0.01608441
10423134	Zfr: zinc finger RNA binding protein	NM_011767	2.246960104	0.01617156
10401997	Ptpn21: protein tyrosine phosphatase, non-receptor type 21	NM_011877	1.856762716	0.016256104
10404178	BC005537: cDNA sequence BC005537	NM_024473	1.541579607	0.016318836
10529260	C330019G07Rik: RIKEN cDNA C330019G07 gene	NM_194340	1.548383681	0.01635935
10361748	Fbxo30: F-box protein 30	NM_027968	1.623474062	0.016447455

10541484	M6pr: mannose-6-phosphate receptor, cation dependent	NM_010749	1.735946059	0.016467304
10531193	Adamts3: a disintegrin-like and metallopeptidase (reprolysin type) witl	NM_177872	1.51848696	0.016552815
10437222	Hnrnpa3: heterogeneous nuclear ribonucleoprotein A3	NM_053263	1.689346099	0.016964255
10556442	Tead1: TEA domain family member 1	NM_001166584	2.021620373	0.016978472
10378732	Crk: v-crk sarcoma virus CT10 oncogene homolog (avian)	NM_133656	1.576581701	0.017033625
10543680	Mir182: microRNA 182	NR_029569	1.757094765	0.017370944
10491962	Foxo1: forkhead box O1	NM_019739	1.626124954	0.017387888
10560242	C5ar1: complement component 5a receptor 1	NM_001173550	1.548505432	0.017391954
10435048	Tctex1d2: Tctex1 domain containing 2	NM_025329	2.019411277	0.017473194
10506058	Inadl: InaD-like (Drosophila)	NM_172696	1.705531772	0.017496787
10595863	Trim42: tripartite motif-containing 42	NM_030219	2.118554155	0.01757439
10474490	Olfr1283: olfactory receptor 1283	NM_207236	1.794459989	0.017699234
10591816	Dpy19l1: dpy-19-like 1 (C. elegans)	NM_172920	1.598168082	0.017726338
10505030	Fsd1l: fibronectin type III and SPRY domain containing 1-like	NM_176966	1.62713863	0.017764928
10458213	Etf1: eukaryotic translation termination factor 1	NM_144866	1.610219479	0.017773109
10428388	Rspo2: R-spondin 2 homolog (Xenopus laevis)	NM_172815	1.891652767	0.017903961
10347491	Wnt6: wingless-related MMTV integration site 6	NM_009526	2.194503279	0.017917002
10597833	Sec22c: SEC22 vesicle trafficking protein homolog C (S. cerevisiae)	NM_178677	1.900794204	0.017928702
10585048	Cadm1: cell adhesion molecule 1	NM_207675	1.55031324	0.017969761
10412543	Gm1973: predicted gene 1973	NM_029288	1.644147373	0.018139746
10519855	Cacna2d1: calcium channel, voltage-dependent, alpha2/delta subunit	NM_001110843	1.549758967	0.018161018
10501265	Gnai3: guanine nucleotide binding protein (G protein), alpha inhibiting	NM_010306	1.661476413	0.018165127
10570321	Cul4a: cullin 4A	NM_146207	1.585509401	0.018184648
10401160	Tmem229b: transmembrane protein 229B	NM_178745	2.060798008	0.018398541
10364130	Zfp280b: zinc finger protein 280B	NM_177475	2.221946094	0.018428022
10587085	BC031353: cDNA sequence BC031353	NM_001113283	1.542144233	0.018434998
10526923	Get4: golgi to ER traffic protein 4 homolog (S. cerevisiae)	NM_026269	1.634311233	0.018444525
10498707	Slitrk3: SLIT and NTRK-like family, member 3	NM_198864	1.959186456	0.018464984
10347933	Sp140: Sp140 nuclear body protein	NM_001013817	1.978446818	0.018480573
10466624	Aldh1a7: aldehyde dehydrogenase family 1, subfamily A7	NM_011921	1.524805864	0.018540775
10435226	Snx4: sorting nexin 4	NM_080557	1.974430143	0.018592065
10560111	Gm5584: predicted gene 5584	NM_001101534	1.650328891	0.018617804
10399214	Rab10: RAB10, member RAS oncogene family	NM_016676	1.858531258	0.018661274
10458841	Ube2l3: ubiquitin-conjugating enzyme E2L 3	NM_009456	2.156416494	0.018686062
10501778	Ptbp2: polypyrimidine tract binding protein 2	NM_019550	1.855401046	0.018749031
10466659	Gda: guanine deaminase	NM_010266	1.634012295	0.018781113
10531208	Cox18: COX18 cytochrome c oxidase assembly homolog (S. cerevisiae)	NM_001163456	1.573155362	0.018798835
10594289	Glce: glucuronyl C5-epimerase	NM_033320	1.637560645	0.018996687
10570013	Abhd13: abhydrolase domain containing 13	NM_001081119	1.516093385	0.01900114
10569485	Tnfrsf26: tumor necrosis factor receptor superfamily, member 26	NM_175649	2.097157856	0.019082543
10379820	Acaca: acetyl-Coenzyme A carboxylase alpha	NM_133360	1.516512521	0.019089315
10386824	Akap10: A kinase (PRKA) anchor protein 10	NM_019921	1.523339468	0.019115293
10487787	Gfra4: glial cell line derived neurotrophic factor family receptor alpha 4	NM_020014	1.635701876	0.019129893
10479950	Celf2: CUGBP, Elav-like family member 2	NM_001110231	1.562898138	0.019139989
10385774	Olfr1371: olfactory receptor 1371	NM_207253	1.798012576	0.019176179
10523281	Sept11: septin 11	NM_001009818	1.914388945	0.019206096
10529218	Supt7l: suppressor of Ty 7 (S. cerevisiae)-like	NM_028150	2.005996077	0.019215965
10368030	Heca: headcase homolog (Drosophila)	NM_001033432	1.679782307	0.01922808
10405488	Tmed9: transmembrane emp24 protein transport domain containing 9	NM_026211	2.037512894	0.019231348
10489660	Elmo2: engulfment and cell motility 2, ced-12 homolog (C. elegans)	NM_207706	1.605826554	0.019392955
10521440	Afap1: actin filament associated protein 1	NM_027373	1.520016382	0.019393591
10393058	H3f3b: H3 histone, family 3B	NM_008211	1.676811466	0.019400341
10408162	Zfp322a: zinc finger protein 322A	NM_001111107	2.225652801	0.019419267
10408113	Hist1h4i: histone cluster 1, H4i	NM_175656	1.686026833	0.019437655
10376929	Fam18b: family with sequence similarity 18, member B	BC115504	1.616950829	0.019450075
10576940	Fam155a: family with sequence similarity 155, member A	AK149049	1.616893169	0.019556066
10389326	Pigw: phosphatidylinositol glycan anchor biosynthesis, class W	NM_027388	1.825269565	0.019569398
10485344	Api5: apoptosis inhibitor 5	NM_007466	1.53037653	0.019596016
10416945	Mirhg1: microRNA host gene 1 (non-protein coding)	NR_029382	1.760353508	0.019613423
10469571	Otud1: OTU domain containing 1	NM_027715	1.809973026	0.019657215
10541446	Cpamd8: C3 and PZP-like, alpha-2-macroglobulin domain containing 8	NM_008646	1.573170732	0.019674374
10348087	Cops7b: COP9 (constitutive photomorphogenic) homolog, subunit 7b (	NM_172974	1.571032186	0.019694813
10508788	Ahdc1: AT hook, DNA binding motif, containing 1	NM_146155	1.520529908	0.01970459
10535378	D430018E03Rik: RIKEN cDNA D430018E03 gene	ENSMUST00000014	1.588150591	0.019716339
10580452	Siah1a: seven in absentia 1A	NM_009172	1.936457012	0.019738842

10424062	A930017M01Rik: RIKEN cDNA A930017M01 gene	NR_033609	1.515638477	0.019994179
10548735	Dusp16: dual specificity phosphatase 16	NM_130447	1.602296753	0.019998295
10544444	Eapa2: experimental autoimmune prostatitis antigen 2	NM_203396	1.602997732	0.020027739
10499948	Lce3a: late cornified envelope 3A	NM_001039594	1.719270596	0.020085284
10489413	Tomm34: translocase of outer mitochondrial membrane 34	NM_025996	2.099773115	0.020107021
10406226	Hnrnpa1l2: heterogeneous nuclear ribonucleoprotein A1-like 2	XM_619124	1.689077465	0.020142048
10495685	Arhgap29: Rho GTPase activating protein 29	NM_172525	1.532021065	0.0201588
10434302	Klhl24: kelch-like 24 (Drosophila)	NM_029436	1.715493983	0.020173374
10545450	Tgoln1: trans-golgi network protein	NM_009443	2.031970862	0.020175786
10564624	St8sia2: ST8 alpha-N-acetyl-neuraminide alpha-2,8-sialyltransferase 2	NM_009181	1.994841806	0.020194109
10419674	Snord8: small nucleolar RNA, C/D box 8	NR_028542	1.972749664	0.020325033
10577633	Golga7: golgi autoantigen, golgin subfamily a, 7	NM_020585	1.662779302	0.02033986
10416023	Scara5: scavenger receptor class A, member 5 (putative)	NM_028903	1.8958469	0.020364938
10395538	Pnpla8: patatin-like phospholipase domain containing 8	NM_026164	1.8387684	0.020365748
10510708	Icmt: isoprenylcysteine carboxyl methyltransferase	NM_133788	1.698732139	0.020501831
10365471	Fbxo7: F-box protein 7	NM_153195	1.624127798	0.020664386
10469278	Il2ra: interleukin 2 receptor, alpha chain	NM_008367	1.704638231	0.020672461
10569646	Ccnd1: cyclin D1	NM_007631	1.556227403	0.020735234
10603698	Gm1549: predicted gene 1549	AJ319753	1.643038634	0.02080873
10531197	Adamts3: a disintegrin-like and metallopeptidase (reprolysin type) witl	NM_177872	1.519506999	0.020912531
10466372	Olfr1504: olfactory receptor 1504	NM_146634	1.799656949	0.021114709
10481827	Zbtb34: zinc finger and BTB domain containing 34	NM_001085507	2.204844576	0.021123754
10438815	1600021P15Rik: RIKEN cDNA 1600021P15 gene	NM_177718	1.503115069	0.021202547
10383833	Ccdc157: coiled-coil domain containing 157	NM_177616	1.553420194	0.02120611
10601760	Hnrnph2: heterogeneous nuclear ribonucleoprotein H2	NM_019868	1.694778189	0.021264685
10346551	Cflar: CASP8 and FADD-like apoptosis regulator	NM_207653	1.565657065	0.021379692
10463070	Entpd1: ectonucleoside triphosphate diphosphohydrolase 1	NM_009848	1.607610103	0.021546119
10544250	E330009J07Rik: RIKEN cDNA E330009J07 gene	ENSMUST00000101	1.602754528	0.021649409
10542575	Pde3a: phosphodiesterase 3A, cGMP inhibited	NM_018779	1.816384455	0.021679858
10542407	Pde6h: phosphodiesterase 6H, cGMP-specific, cone, gamma	NM_023898	1.816384455	0.021679858
10386470	4930412M03Rik: RIKEN cDNA 4930412M03 gene	ENSMUST00000057	1.509265846	0.021796467
10498337	Clrn1: clarin 1	NM_153384	1.569129474	0.021921856
10572880	Tom1: target of myb1 homolog (chicken)	NM_011622	2.099429688	0.021930113
10402249	Atxn3: ataxin 3	NM_029705	1.540111105	0.021954142
10479794	Prpf18: PRP18 pre-mRNA processing factor 18 homolog (yeast)	NM_026045	1.855269979	0.022109393
10482336	Lrp1b: low density lipoprotein-related protein 1B (deleted in tumors)	NM_053011	1.730138548	0.022158753
10521972	Pcdh7: protocadherin 7	NM_018764	1.815914609	0.022181504
10391348	Fam134c: family with sequence similarity 134, member C	BC016089	1.615986217	0.022255791
10507284	Gm12950: predicted gene 12950	XR_031009	1.642328016	0.022279005
10366052	Kitl: kit ligand	NM_013598	1.713542339	0.02234109
10515164	Cmpk1: cytidine monophosphate (UMP-CMP) kinase 1	NM_025647	1.569325837	0.022505978
10516551	S100pbp: S100P binding protein	NM_029036	1.893285337	0.022517527
10345083	Khdc1b: KH domain containing 1B	NM_001113187	1.711540411	0.022533871
10394671	Gm16497: predicted gene 16497	AY512908	1.643679308	0.02254898
10592382	Olfr934: olfactory receptor 934	NM_146442	1.806532347	0.022589072
10511975	Slc35a1: solute carrier family 35 (CMP-sialic acid transporter), membe	NM_011895	1.952780229	0.022671795
10362803	Cd164: CD164 antigen	NM_016898	1.557190134	0.022709478
10520952	Ppp1cb: protein phosphatase 1, catalytic subunit, beta isoform	NM_172707	1.847928616	0.022714764
10463737	Ina: internexin neuronal intermediate filament protein, alpha	ENSMUST00000037	1.705353479	0.022744092
10498599	Ift80: intraflagellar transport 80 homolog (Chlamydomonas)	NM_026641	1.700677723	0.022786633
10435841	Ccdc52: coiled-coil domain containing 52	NM_144550	1.554390155	0.022826971
10381934	Tanc2: tetratricopeptide repeat, ankyrin repeat and coiled-coil contain	NM_181071	2.012533247	0.02290332
10385052	Ranbp17: RAN binding protein 17	NM_023146	1.873098951	0.022913237
10602428	Wnk3: WNK lysine deficient protein kinase 3	ENSMUST00000096	2.191848397	0.022925219
10469335	Stam: signal transducing adaptor molecule (SH3 domain and ITAM mo	NM_011484	1.995358647	0.022934127
10580649	Es22: esterase 22	NM_133660	1.610003112	0.023070901
10506050	Nfia: nuclear factor I/A	NM_001122952	1.78028028	0.023268688
10592457	Olfr976: olfactory receptor 976	NM_146367	1.807493333	0.02328974
10358754	Gm7278: predicted gene 7278	XR_002131	1.656766391	0.023341634
10404702	Gcnt2: glucosaminyl (N-acetyl) transferase 2, I-branching enzyme	NM_023887	1.631447432	0.023418586
10403558	Ero1lb: ERO1-like beta (S. cerevisiae)	NM_026184	1.609215852	0.023495976
10512847	Alg2: asparagine-linked glycosylation 2 homolog (yeast, alpha-1,3-mar	NM_019998	1.5255942	0.023511424
10563355	Sec1: secretory blood group 1	NM_019934	1.896765381	0.023512674
10357472	Cxcr4: chemokine (C-X-C motif) receptor 4	NM_009911	1.585706082	0.023529526
10517173	Ccdc21: coiled-coil domain containing 21	NM_144527	1.553801382	0.023573143

10490773	Hnrnph2: heterogeneous nuclear ribonucleoprotein H2	NM_019868	1.694899932	0.023646003
10504817	Tgfbr1: transforming growth factor, beta receptor I	NM_009370	2.025739987	0.02368021
10434733	Eif4a2: eukaryotic translation initiation factor 4A2	NM_013506	1.605556633	0.023690809
10422179	Pou4f1: POU domain, class 4, transcription factor 1	NM_011143	1.847174133	0.023694931
10415377	2610027L16Rik: RIKEN cDNA 2610027L16 gene	NM_026403	1.508253983	0.023729776
10388869	Tnfaip1: tumor necrosis factor, alpha-induced protein 1 (endothelial)	NM_009395	2.094664148	0.02373848
10532944	Mlec: malectin	NM_175403	1.761841637	0.023758964
10378126	Ankfy1: ankyrin repeat and FYVE domain containing 1	NM_009671	1.529013204	0.023818971
10600726	Mageb1: melanoma antigen, family B, 1	NM_010759	1.737502067	0.023899877
10607841	Tceanc: transcription elongation factor A (SII) N-terminal and central d	NM_001007577	2.018631458	0.024077118
10458834	Atg12: autophagy-related 12 (yeast)	NM_026217	1.538207597	0.024175434
10397145	Acot2: acyl-CoA thioesterase 2	NM_134188	1.517169061	0.024203192
10557009	Eef2k: eukaryotic elongation factor-2 kinase	NM_007908	1.604030432	0.024208932
10454478	Polr2d: polymerase (RNA) II (DNA directed) polypeptide D	NM_027002	1.842489074	0.024360535
10414417	Peli2: pellino 2	NM_033602	1.818683681	0.024544893
10453573	Olfr63: olfactory receptor 63	NM_146937	1.80465127	0.024600637
10586614	C2cd4b: C2 calcium-dependent domain containing 4B	NM_001081314	1.547183831	0.024611563
10474508	Olfr1299: olfactory receptor 1299	NM_146884	1.794990154	0.024815346
10458098	Pkd2l2: polycystic kidney disease 2-like 2	NM_016927	1.831632653	0.024842666
10352777	Slc30a1: solute carrier family 30 (zinc transporter), member 1	NM_009579	1.947376261	0.024885894
10465812	Ttc9c: tetratricopeptide repeat domain 9C	NM_027412	2.138703091	0.024898382
10554323	Mir9-3: microRNA 9-3	NR_029818	1.759687106	0.024906603
10393881	Mafg: v-maf musculoaponeurotic fibrosarcoma oncogene family, prote	NM_010756	1.737033921	0.024928451
10420637	Kpna3: karyopherin (importin) alpha 3	NM_008466	1.717406589	0.024965104
10449284	Dusp1: dual specificity phosphatase 1	NM_013642	1.599888746	0.02509238
10536746	Arf5: ADP-ribosylation factor 5	NM_007480	1.531978022	0.025099813
10417759	Ube2e2: ubiquitin-conjugating enzyme E2E 2 (UBC4/5 homolog, yeast	NM_144839	2.146750918	0.02510939
10577641	1810011O10Rik: RIKEN cDNA 1810011O10 gene	NM_026931	1.5040257	0.025130134
10440238	Nsun3: NOL1/NOP2/Sun domain family member 3	NM_178925	1.784860558	0.025184176
10434778	Rtp4: receptor transporter protein 4	NM_023386	1.892038827	0.025189869
10394862	Asap2: ArfGAP with SH3 domain, ankyrin repeat and PH domain 2	NM_001135192	1.535700119	0.025229012
10363265	Lims1: LIM and senescent cell antigen-like domains 1	NM_026148	1.722299754	0.025262572
10528901	C79130: expressed sequence C79130	AK147752	1.54886258	0.025276332
10600593	Hnrnpa3: heterogeneous nuclear ribonucleoprotein A3	NM_053263	1.692654	0.025300523
10366293	Csrp2: cysteine and glycine-rich protein 2	NM_007792	1.580633839	0.025358073
10544017	Svopl: SV2 related protein homolog (rat)-like	NM_177200	2.009789103	0.025413912
10570955	Golga7: golgi autoantigen, golgin subfamily a, 7	NM_020585	1.664493268	0.025475715
10444589	Hspa1a: heat shock protein 1A	NM_010479	1.696661354	0.02554515
10390473	Pip4k2b: phosphatidylinositol-5-phosphate 4-kinase, type II, beta	NM_054051	1.829113246	0.02570994
10480492	Cacna1b: calcium channel, voltage-dependent, N type, alpha 1B subur	NM_001042528	1.549373152	0.02573502
10493916	Flg2: filaggrin family member 2	NM_001013804	1.625415815	0.025741744
10422272	Sox21: SRY-box containing gene 21	NM_177753	1.977320452	0.025748935
10462521	Pten: phosphatase and tensin homolog	NM_008960	1.856557887	0.025765889
10404187	Tdp2: tyrosyl-DNA phosphodiesterase 2	NM_019551	2.020761796	0.025851473
10413839	Ncoa4: nuclear receptor coactivator 4	NM_001033988	1.776203274	0.025900648
10400981	D830013O20Rik: RIKEN cDNA D830013O20 gene	ENSMUST00000070	1.588910561	0.026191398
10555919	Olfr692: olfactory receptor 692	NM_146355	1.805617701	0.026404844
10363161	Tmem229b: transmembrane protein 229B	NM_178745	2.065341213	0.026436082
10352393	Srp9: signal recognition particle 9	NM_012058	1.985387238	0.026452088
10387180	Ndel1: nuclear distribution gene E-like homolog 1 (A. nidulans)	NM_023668	1.776782524	0.026476643
10508974	Pafah2: platelet-activating factor acetylhydrolase 2	NM_133880	1.812310121	0.026536225
10581729	Ddx19a: DEAD (Asp-Glu-Ala-Asp) box polypeptide 19a	NM_007916	1.590101354	0.026542995
10582287	Car5a: carbonic anhydrase 5a, mitochondrial	NM_007608	1.550994015	0.026579416
10415092	4930579G18Rik: RIKEN cDNA 4930579G18 gene	ENSMUST00000038	1.510776477	0.026620916
10394040	Csnk1d: casein kinase 1, delta	NM_027874	1.580495905	0.026724662
10448811	Cramp1l: Crm, cramped-like (Drosophila)	NM_020608	1.575946217	0.026760451
10564573	Chd2: chromodomain helicase DNA binding protein 2	NM_001081345	1.566394645	0.026768745
10409376	Hk3: hexokinase 3	NM_001033245	1.688158548	0.026842549
10428302	Klf10: Kruppel-like factor 10	NM_013692	1.71359078	0.026940885
10533569	Kdm2b: lysine (K)-specific demethylase 2B	NM_001003953	1.71027275	0.027016952
10545672	Mthfd2: methylenetetrahydrofolate dehydrogenase (NAD+ dependent	NM_008638	1.771985931	0.027042221
10522192	Nsun7: NOL1/NOP2/Sun domain family, member 7	NM_027602	1.785581129	0.027191281
10555848	Trim6: tripartite motif-containing 6	NM_001013616	2.120337557	0.027256384
10460573	Eif1ad: eukaryotic translation initiation factor 1A domain containing	NM_027236	1.605282974	0.027258813
10546510	Lrig1: leucine-rich repeats and immunoglobulin-like domains 1	NM_008377	1.729726009	0.027405334

10560117	Gm6955: predicted gene 6955	ENSMUST00000098	1.654609262	0.027459878
10567022	Btbd10: BTB (POZ) domain containing 10	NM_133700	1.543731579	0.027461517
10444386	Gpsm3: G-protein signalling modulator 3 (AGS3-like, C. elegans)	NM_134116	1.671722365	0.027511654
10380629	Hoxb8: homeobox B8	NM_010461	1.696103896	0.027536283
10383684	Limk2: LIM motif-containing protein kinase 2	NM_010718	1.721822864	0.027598107
10489078	Dsn1: DSN1, MIND kinetochore complex component, homolog (S. cere	NM_025853	1.598761583	0.027669268
10455514	Kcnn2: potassium intermediate/small conductance calcium-activated c	NM_080465	1.708324025	0.027710202
10353899	Sema4c: sema domain, immunoglobulin domain (Ig), transmembrane	NM_001126047	1.903855121	0.027755087
10452556	Rab12: RAB12, member RAS oncogene family	NM_024448	1.858781405	0.027772184
10494200	Cdc42se1: CDC42 small effector 1	NM_172395	1.560367443	0.027802884
10562368	4931406P16Rik: RIKEN cDNA 4931406P16 gene	BC060233	1.512147387	0.027817194
10604974	Cetn2: centrin 2	NM_019405	1.563066007	0.027825527
10496169	Ppa2: pyrophosphatase (inorganic) 2	NM_146141	1.847432655	0.027956879
10402117	Rps6ka5: ribosomal protein S6 kinase, polypeptide 5	NM_153587	1.888570357	0.027988955
10500896	Gm6485: predicted gene 6485	XR_002221	1.652751706	0.028037207
10358698	Rnf2: ring finger protein 2	NM_011277	1.886780553	0.028112474
10537499	Tas2r137: taste receptor, type 2, member 137	NM_001025385	2.013584282	0.028128204
10581737	Ddx19b: DEAD (Asp-Glu-Ala-Asp) box polypeptide 19b	NM_172284	1.5919387	0.028197092
10481393	Zdhhc12: zinc finger, DHHC domain containing 12	NM_025428	2.210062831	0.028266875
10567043	Rras2: related RAS viral (r-ras) oncogene homolog 2	NM_025846	1.891584925	0.028442
10414479	Olfr740: olfactory receptor 740	NM_146667	1.805800186	0.028551148
10602716	Ubqln2: ubiquilin 2	NM_018798	2.163807024	0.028578807
10545760	Paip2b: poly(A) binding protein interacting protein 2B	NM_146169	1.814168324	0.028617798
10543204	Tmem168: transmembrane protein 168	NM_028990	2.037762773	0.028632745
10451650	Nfya: nuclear transcription factor-Y alpha	NM_001110832	1.780554094	0.028710834
10439268	Dtx3l: deltex 3-like (Drosophila)	NM_001013371	1.599503207	0.028776782
10389238	Dusp14: dual specificity phosphatase 14	NM_019819	1.600749671	0.028844717
10542317	Cdkn1b: cyclin-dependent kinase inhibitor 1B	NM_009875	1.561860773	0.028906362
10456120	Csnk1a1: casein kinase 1, alpha 1	NM_146087	1.5798183	0.028935942
10404049	Hist1h3d: histone cluster 1, H3d	NM_178204	1.684186498	0.029083797
10377550	Trp53: transformation related protein 53	NM_001127233	2.121682048	0.029133399
10389627	Rad51c: RAD51 homolog c (S. cerevisiae)	NM_053269	1.872250297	0.029182367
10453256	Kcng3: potassium voltage-gated channel, subfamily G, member 3	NM_153512	1.707151547	0.02922126
10474207	Fbxo3: F-box protein 3	NM_212433	1.623330342	0.029273122
10576124	Ctu2: cytosolic thiouridylase subunit 2 homolog (S. pombe)	NM_153775	1.584715822	0.029281578
10368720	Slc16a10: solute carrier family 16 (monocarboxylic acid transporters),	NM_001114332	1.940827071	0.029286071
10446739	Clip4: CAP-GLY domain containing linker protein family, member 4	NM_030179	1.569012562	0.029366867
10566926	Rnf141: ring finger protein 141	NM_025999	1.883040936	0.029430356
10413989	Gm626: predicted gene 626	XM_985917	1.651500158	0.029515279
10354677	Ankrd44: ankyrin repeat domain 44	NM_001081433	1.529838929	0.029575675
10585358	Npat: nuclear protein in the AT region	NM_001081152	1.781979188	0.02964377
10554057	Adamts17: a disintegrin-like and metallopeptidase (reprolysin type) wi	NM_001033877	1.518226328	0.029725333
10528527	Fam126a: family with sequence similarity 126, member A	NM_053090	1.614285714	0.029732717
10458992	C330018D20Rik: RIKEN cDNA C330018D20 gene	NM_029909	1.547479387	0.029836217
10471770	Olfr357: olfactory receptor 357	NM_146623	1.800992083	0.02990865
10357064	Vps4b: vacuolar protein sorting 4b (yeast)	NM_009190	2.184706184	0.030494607
10535852	Slc7a1: solute carrier family 7 (cationic amino acid transporter, y+ syst	NM_007513	1.955973311	0.030522117
10542112	Clec2h: C-type lectin domain family 2, member h	NM_053165	1.568114608	0.030537753
10408156	Vmn1r217: vomeronasal 1 receptor 217	NM_134239	2.177539254	0.030569625
10422280	Abcc4: ATP-binding cassette, sub-family C (CFTR/MRP), member 4	NM_001033336	1.516086706	0.030605211
10595856	Slc25a36: solute carrier family 25, member 36	NM_138756	1.945302469	0.030625318
10516241	Maneal: mannosidase, endo-alpha-like	NM_001007573	1.739846126	0.030704874
10426110	Pim3: proviral integration site 3	NM_145478	1.82698344	0.030805533
10373530	Cdk2: cyclin-dependent kinase 2	NM_183417	1.561156231	0.030829885
10347779	9830004L10Rik: RIKEN cDNA 9830004L10 gene	AK137457	1.515406736	0.030840639
10543471	Pot1a: protection of telomeres 1A	NM_133931	1.843931538	0.030965543
10389507	Tmem49: transmembrane protein 49	NM_029478	2.068641888	0.031023892
10592023	Aplp2: amyloid beta (A4) precursor-like protein 2	NM_001102455	1.531733574	0.031211614
10595768	Pls1: plastin 1 (I-isoform)	NM_001033210	1.836759566	0.031337639
10441511	Tmem181a: transmembrane protein 181A	NM_001033178	2.049572744	0.031362728
10462333	Cdc37l1: cell division cycle 37 homolog (S. cerevisiae)-like 1	NM_025950	1.558809112	0.031374618
10537728	Casp2: caspase 2	NM_007610	1.551889788	0.031400991
10515080	4930522H14Rik: RIKEN cDNA 4930522H14 gene	BC125547	1.510102019	0.031428588
10605664	Mageb1: melanoma antigen, family B, 1	NM_010759	1.738304089	0.031483803
10601915	Fam199x: family with sequence similarity 199, X-linked	NM_146261	1.617831766	0.031500815

10604595	Mir106a: microRNA 106a	NR_029657	1.752472008	0.031617544
10390816	Krt27: keratin 27	NM_010666	1.717790399	0.031756562
10425109	Elfn2: leucine rich repeat and fibronectin type III, extracellular 2	NM_183141	1.605783147	0.031828682
10553071	Ntn5: netrin 5	NM_001033356	1.78599055	0.031832974
10468309	Sh3pxd2a: SH3 and PX domains 2A	NM_008018	1.934873863	0.031885382
10525961	Piwil1: piwi-like homolog 1 (Drosophila)	NM_021311	1.830243059	0.03193753
10350823	Abl2: v-abl Abelson murine leukemia viral oncogene homolog 2 (arg, A	NM_001136104	1.516376054	0.03195413
10578361	Asah1: N-acylsphingosine amidohydrolase 1	NM_019734	1.535597942	0.031955003
10411156	Scamp1: secretory carrier membrane protein 1	NM_029153	1.894718059	0.032006432
10607064	Gucy2f: guanylate cyclase 2f	NM_001007576	1.672760703	0.032067297
10588294	Topbp1: topoisomerase (DNA) II binding protein 1	NM_176979	2.104110093	0.032080924
10445944	St6gal2: beta galactoside alpha 2,6 sialyltransferase 2	NM_172829	1.994457015	0.032163214
10543428	Iqub: IQ motif and ubiquitin domain containing	NM_172535	1.706236999	0.032166726
10443449	Kctd20: potassium channel tetramerisation domain containing 20	NM_025888	1.708856549	0.03222751
10567851	Nfatc2ip: nuclear factor of activated T-cells, cytoplasmic, calcineurin-d	NM_010900	1.778438695	0.032252802
10462973	Hells: helicase, lymphoid specific	NM_008234	1.681582725	0.032265497
10347796	Rhbdd1: rhomboid domain containing 1	NM_029777	1.881708435	0.032362047
10601993	D330045A20Rik: RIKEN cDNA D330045A20 gene	NM_175326	1.587962557	0.032443821
10538932	Amd1: S-adenosylmethionine decarboxylase 1	NM_009665	1.526724004	0.03250762
10596347	Atp2c1: ATPase, Ca++-sequestering	NM_175025	1.539630677	0.032515478
10606694	Btk: Bruton agammaglobulinemia tyrosine kinase	NM_013482	1.544836481	0.032700421
10555785	Olfr598: olfactory receptor 598	NM_001011793	1.803848669	0.032846396
10444719	Bat3: HLA-B-associated transcript 3	NM_057171	1.541568443	0.033038244
10549396	Gm6288: predicted gene 6288	ENSMUST00000100	1.651682507	0.03313646
10601424	Gpr174: G protein-coupled receptor 174	NM_001177781	1.667897948	0.033202395
10440329	9330155M09Rik: RIKEN cDNA 9330155M09 gene	AK038707	1.51514203	0.033250025
10475932	Fbln7: fibulin 7	NM_024237	1.621474779	0.033276653
10410892	Rasa1: RAS p21 protein activator 1	NM_145452	1.874184539	0.033293108
10488147	Flrt3: fibronectin leucine rich transmembrane protein 3	NM_001172160	1.625661448	0.033337716
10414262	Ear2: eosinophil-associated, ribonuclease A family, member 2	NM_007895	1.603228789	0.033396507
10447510	Amd1: S-adenosylmethionine decarboxylase 1	NM_009665	1.527011743	0.033407133
10412466	Hmgcs1: 3-hydroxy-3-methylglutaryl-Coenzyme A synthase 1	NM_145942	1.688899665	0.033436159
10366983	Tmem194: transmembrane protein 194	NM_001113211	2.05956047	0.033482658
10400006	Ahr: aryl-hydrocarbon receptor	NM_013464	1.520539438	0.033516719
10367033	Zbtb39: zinc finger and BTB domain containing 39	NM_198035	2.207356586	0.03360218
10581336	Gfod2: glucose-fructose oxidoreductase domain containing 2	NM_027469	1.634445283	0.033715376
10576772	Clec4g: C-type lectin domain family 4, member g	AK016282	1.568367177	0.033749277
10462480	Cstf2t: cleavage stimulation factor, 3' pre-RNA subunit 2, tau	NM_031249	1.581302414	0.033773827
10497904	Nudt6: nudix (nucleoside diphosphate linked moiety X)-type motif 6	NM_153561	1.788170369	0.033799946
10535508	AU022870: expressed sequence AU022870	BC048077	1.540503818	0.033965634
10395287	Atxn7l1: ataxin 7-like 1	NM_028139	1.540503818	0.033965634
10565996	Inppl1: inositol polyphosphate phosphatase-like 1	NM_010567	1.706121203	0.033973326
10568221	Sephs2: selenophosphate synthetase 2	NM_009266	1.914000719	0.034022773
10478447	Stk4: serine/threonine kinase 4	NM_021420	2.000187207	0.034086345
10517373	Rcan3: regulator of calcineurin 3	NM_022980	1.87784674	0.034220636
10439744	Cd96: CD96 antigen	NM_032465	1.557480237	0.034488298
10574436	Khdrbs1: KH domain containing, RNA binding, signal transduction asso	NM_011317	1.71262431	0.034572021
10406287	Ttc37: tetratricopeptide repeat domain 37	NM_001081352	2.137598959	0.034622259
10419038	Ghitm: growth hormone inducible transmembrane protein	NM_078478	1.637363535	0.034719508
10578880	Tll1: tolloid-like	NM_009390	2.032892217	0.034778236
10479979	Slc25a36: solute carrier family 25, member 36	NM_138756	1.945765026	0.03488221
10443459	Sfrs3: splicing factor, arginine/serine-rich 3 (SRp20)	NM_013663	1.931431327	0.034900099
10369176	D630037F22Rik: RIKEN cDNA D630037F22 gene	NM_001033385	1.588192998	0.034915856
10483667	Cir1: corepressor interacting with RBPJ, 1	NM_025854	1.567453391	0.034984086
10369704	Hnrnph3: heterogeneous nuclear ribonucleoprotein H3	NM_001079824	1.694967737	0.035024592
10568464	Ate1: arginyltransferase 1	NM_013799	1.53696882	0.035081084
10394971	Klf11: Kruppel-like factor 11	NM_178357	1.713801245	0.035153135
10557853	B230325K18Rik: RIKEN cDNA B230325K18 gene	ENSMUST00000079	1.541122472	0.035207073
10499981	Oaz3: ornithine decarboxylase antizyme 3	NM_016901	1.788857569	0.035242649
10385004	Nprl3: nitrogen permease regulator-like 3 (S. cerevisiae)	NM_181569	1.782029903	0.035283893
10409943	Ctsm: cathepsin M	NM_022326	1.582674779	0.035405946
10428222	Ncald: neurocalcin delta	NM_134094	1.775781718	0.035493004
10364293	Ube2g2: ubiquitin-conjugating enzyme E2G 2	NM_019803	2.149472821	0.035503287
10585982	Myo9a: myosin IXa	NM_173018	1.774623315	0.035526339
10530960	Tmprss11d: transmembrane protease, serine 11d	NM_145561	2.085521719	0.035550709

10570429	Zfp828: zinc finger protein 828	NM_181854	2.244805781	0.035790917
10498415	Dhx36: DEAH (Asp-Glu-Ala-His) box polypeptide 36	NM_028136	1.594973704	0.035801084
10491038	Tbl1xr1: transducin (beta)-like 1X-linked receptor 1	NM_030732	2.015380104	0.035815776
10545583	Pole4: polymerase (DNA-directed), epsilon 4 (p12 subunit)	NM_025882	1.840966461	0.035869083
10576934	Fam155a: family with sequence similarity 155, member A	NM_173446	1.616515168	0.036065908
10397054	Dcaf4: DDB1 and CUL4 associated factor 4	NM_001165256	1.589054319	0.036122792
10394922	Gm6950: predicted pseudogene 6950	ENSMUST00000080	1.654592352	0.036126413
10394331	Pfn4: profilin family, member 4	NM_028376	1.819379739	0.036223977
10473760	Celf1: CUGBP, Elav-like family member 1	NM_017368	1.562859882	0.036265596
10440417	9430053O09Rik: RIKEN cDNA 9430053O09 gene	AK140789	1.515324018	0.036387996
10404038	Hist1h3d: histone cluster 1, H3d	NM_178204	1.684309823	0.03649379
10502191	Ostc: oligosaccharyltransferase complex subunit	NM_025509	1.808945639	0.036641709
10369867	Tfam: transcription factor A, mitochondrial	NM_009360	2.022066349	0.036646431
10428310	Azin1: antizyme inhibitor 1	NM_001102458	1.540516844	0.036680153
10556769	Acsm3: acyl-CoA synthetase medium-chain family member 3	NM_016870	1.51768849	0.036857965
10459837	8030462N17Rik: RIKEN cDNA 8030462N17 gene	BC120889	1.514469379	0.036883056
10374466	Rab1: RAB1, member RAS oncogene family	NM_008996	1.85776942	0.036899484
10508907	Lin28a: lin-28 homolog A (C. elegans)	NM_145833	1.722333228	0.036954582
10502451	Bmpr1b: bone morphogenetic protein receptor, type 1B	NM_007560	1.542746604	0.037053892
10407993	Sfrs13a: splicing factor, arginine/serine-rich 13A	NM_001080387	1.929041639	0.037064725
10590325	Ctnnb1: catenin (cadherin associated protein), beta 1	NM_007614	1.581782983	0.037272353
10605662	Gm5072: predicted gene 5072	NM_001114678	1.649652644	0.037530175
10424379	Sfrs3: splicing factor, arginine/serine-rich 3 (SRp20)	NM_013663	1.931500689	0.037649454
10594322	Pias1: protein inhibitor of activated STAT 1	NM_019663	1.821328078	0.037664253
10429547	Cyp11b2: cytochrome P450, family 11, subfamily b, polypeptide 2	NM_009991	1.586542758	0.037666802
10347106	Rpe: ribulose-5-phosphate-3-epimerase	NM_025683	1.888436821	0.037764829
10462881	Gpr120: G protein-coupled receptor 120	NM_181748	1.666361574	0.037848971
10466274	Olfr1419: olfactory receptor 1419	BC147200	1.798396465	0.037893334
10517486	Gm12986: heterogeneous nuclear ribonucleoprotein A3 pseudogene	XR_031848	1.64255729	0.038067802
10498302	Gm410: predicted gene 410	NM_001033349	1.647035782	0.038098782
10458046	D0H4S114: DNA segment, human D4S114	NM_053078	1.586608494	0.038106615
10562416	Cebpg: CCAAT/enhancer binding protein (C/EBP), gamma	NM_009884	1.562658556	0.038108399
10347460	Ttll4: tubulin tyrosine ligase-like family, member 4	NM_001014974	2.14137648	0.038258189
10561474	Il28a: interleukin 28A	NM_001024673	1.703541383	0.038270286
10391990	1700081L11Rik: RIKEN cDNA 1700081L11 gene	BC054752	1.503393313	0.038312624
10474524	Olfr1318: olfactory receptor 1318	NM_001011802	1.797177361	0.038369585
10350896	Astn1: astrotactin 1	NM_007495	1.53608365	0.038385017
10607156	Dcx: doublecortin	NM_001110222	1.589320537	0.038394192
10379989	Fam33a: family with sequence similarity 33, member A	NM_025377	1.618041638	0.038443504
10407022	Gm4938: H3 histone, family 3 pseudogene	XM_138832	1.647469833	0.038465159
10548940	Lmo3: LIM domain only 3	NM_207222	1.72460666	0.038490732
10368817	Ccdc162: coiled-coil domain containing 162	NM_001177571	1.553427437	0.038668474
10404521	Bphl: biphenyl hydrolase-like (serine hydrolase, breast epithelial mucir	NM_026512	1.543185095	0.038688864
10515113	Hnrnpa3: heterogeneous nuclear ribonucleoprotein A3	BC062198	1.694280534	0.038716546
10518372	Miip: migration and invasion inhibitory protein	NM_001025365	1.751005951	0.0387523
10492220	2810407C02Rik: RIKEN cDNA 2810407C02 gene	NM_001040396	1.508682896	0.038807366
10512935	Amd1: S-adenosylmethionine decarboxylase 1	NM_009665	1.528803658	0.038975085
10526520	Plod3: procollagen-lysine, 2-oxoglutarate 5-dioxygenase 3	NM_011962	1.836695593	0.039104321
10401630	Acyp1: acylphosphatase 1, erythrocyte (common) type	NM_025421	1.518211947	0.039162933
10392560	Abca9: ATP-binding cassette, sub-family A (ABC1), member 9	NM_147220	1.515743269	0.039180754
10433492	Atf7ip2: activating transcription factor 7 interacting protein 2	NM_153123	1.53815801	0.039256997
10577340	Defb37: defensin beta 37	NM_181683	1.593273067	0.039308301
10371589	Gm10763: predicted gene 10763	ENSMUST00000099	1.641890589	0.039363147
10367717	BC013529: cDNA sequence BC013529	NM_145418	1.541762276	0.039387761
10410604	Ahrr: aryl-hydrocarbon receptor repressor	NM_009644	1.521134431	0.039432432
10574560	2310038E17Rik: RIKEN cDNA 2310038E17 gene	BC117742	1.506050091	0.039475784
10421774	Dgkh: diacylglycerol kinase, eta	NM_001081336	1.593329998	0.039650735
10478875	Rnf114: ring finger protein 114	NM_030743	1.882735716	0.039667244
10417415	Gm1973: predicted gene 1973	NM_029288	1.645276164	0.039672898
10480605	Cobra1: cofactor of BRCA1	NM_021393	1.570536294	0.03980066
10416421	Lrch1: leucine-rich repeats and calponin homology (CH) domain contai	NM_001033439	1.729667175	0.039840002
10466317	Zfp91-Cntf: Zfp91-Cntf readthrough transcript	NR_024093	2.24668913	0.039965021
10510574	Errfi1: ERBB receptor feedback inhibitor 1	NM_133753	1.609755423	0.039967353
10399908	Prkar2b: protein kinase, cAMP dependent regulatory, type II beta	NM_011158	1.850724638	0.040069994
10358607	Hmcn1: hemicentin 1	NM_001024720	1.688320104	0.040154345

10398455	Ppp2r5c: protein phosphatase 2, regulatory subunit B (B56), gamma is	NM_001135001	1.849016549	0.040198154
10550047	Zscan22: zinc finger and SCAN domain containing 22	NM_001001447	2.253535432	0.04027063
10521796	Mir218-1: microRNA 218-1	NR_029798	1.759662761	0.040337981
10582862	Arhgef12: Rho guanine nucleotide exchange factor (GEF) 12	NM_027144	1.53258019	0.040369878
10369911	1110038D17Rik: RIKEN cDNA 1110038D17 gene	BC118613	1.50247861	0.04038709
10546567	A130022J15Rik: RIKEN cDNA A130022J15 gene	BC048939	1.515530113	0.040434176
10479627	Tpd52l2: tumor protein D52-like 2	NM_025482	2.109752744	0.040459088
10424370	Trib1: tribbles homolog 1 (Drosophila)	NM_144549	2.109774268	0.040460854
10592154	Hyls1: hydrolethalus syndrome 1	NM_029762	1.696861711	0.040513277
10372417	4921506J03Rik: RIKEN cDNA 4921506J03 gene	NM_001033474	1.509189411	0.040553914
10600341	Emd: emerin	NM_007927	1.607193099	0.040581312
10544596	Tmem176b: transmembrane protein 176B	NM_023056	2.044987442	0.040649511
10472350	Gca: grancalcin	NM_145523	1.631053989	0.040653399
10361712	Shprh: SNF2 histone linker PHD RING helicase	NM_172937	1.934953144	0.040708055
10409240	Sema4d: sema domain, immunoglobulin domain (Ig), transmembrane	NM_013660	1.90513834	0.04082031
10384656	B3gnt2: UDP-GlcNAc:betaGal beta-1,3-N-acetylglucosaminyltransferas	NM_016888	1.541139955	0.040832804
10449839	Akap8: A kinase (PRKA) anchor protein 8	NM_019774	1.524546425	0.040947083
10494857	Nras: neuroblastoma ras oncogene	NM_010937	1.782397627	0.041005543
10607933	Hccs: holocytochrome c synthetase	NM_008222	1.678638726	0.041079885
10395198	Gm9359: tripartite motif-containing 13 pseudogene	XR_032641	1.660323089	0.041118808
10484307	Frzb: frizzled-related protein	NM_011356	1.626660979	0.041188466
10357562	Il20: interleukin 20	NM_021380	1.703284966	0.04122377
10494574	Prkab2: protein kinase, AMP-activated, beta 2 non-catalytic subunit	NM_182997	1.850166756	0.041247755
10422067	Fbxl3: F-box and leucine-rich repeat protein 3	NM_015822	1.622384956	0.041302159
10411751	Slc30a5: solute carrier family 30 (zinc transporter), member 5	NM_022885	1.949121042	0.041330778
10375634	Mapk9: mitogen-activated protein kinase 9	NM_001163672	1.743845878	0.041401839
10345411	Arhgef4: Rho guanine nucleotide exchange factor (GEF) 4	NM_183019	1.534456578	0.041446901
10573027	Otud4: OTU domain containing 4	NM_001081164	1.810716424	0.041477597
10374773	Mir216a: microRNA 216a	NR_029797	1.759188847	0.041490976
10557470	Gdpd3: glycerophosphodiester phosphodiesterase domain containing	NM_024228	1.634058564	0.041681043
10400089	Gm889: predicted gene 889	BC147387	1.659344728	0.041772019
10573160	4933434I20Rik: RIKEN cDNA 4933434I20 gene	NM_026233	1.512994295	0.041788605
10536527	Capza2: capping protein (actin filament) muscle Z-line, alpha 2	NM_007604	1.550691244	0.041826926
10532308	4930522L14Rik: RIKEN cDNA 4930522L14 gene	XM_001476915	1.510555675	0.041998554
10492045	Hnrnpa3: heterogeneous nuclear ribonucleoprotein A3	NM_053263	1.693700639	0.042024986
10345077	Khdc1a: KH domain containing 1A	NM_183322	1.71067685	0.042133094
10400350	Cfl2: cofilin 2, muscle	NM_007688	1.563275706	0.042342971
10368681	Rfpl4b: ret finger protein-like 4B	NM_001177783	1.878913289	0.042552571
10361995	Fam54a: family with sequence similarity 54, member A	NM_027930	1.619745777	0.042591512
10501676	Hiat1: hippocampus abundant gene transcript 1	NM_008246	1.683905016	0.042605206
10345079	Khdc1a: KH domain containing 1A	NM_183322	1.71053905	0.042606029
10411287	Btf3l4: basic transcription factor 3-like 4	NM_027453	1.543992787	0.042912961
10501489	Hectd1: HECT domain containing 1	NM_144788	1.681276805	0.043085507
10417281	Gm1973: predicted gene 1973	NM_029288	1.644660217	0.043307579
10582978	8430410K20Rik: RIKEN cDNA 8430410K20 gene	BC014729	1.514701157	0.043328892
10404848	Jarid2: jumonji, AT rich interactive domain 2	NM_021878	1.706544287	0.04332935
10447517	Pisd-ps2: phosphatidylserine decarboxylase, pseudogene 2	NR_003519	1.829465372	0.043581339
10576532	Tsnax: translin-associated factor X	NM_016909	2.128099602	0.043658296
10379998	Trim37: tripartite motif-containing 37	NM_197987	2.116111945	0.043831736
10573893	Fto: fat mass and obesity associated	NM_011936	1.630703875	0.043943685
10520544	Mapre3: microtubule-associated protein, RP/EB family, member 3	NM_133350	1.744706831	0.043998523
10410947	Xrcc4: X-ray repair complementing defective repair in Chinese hamste	NM_028012	2.194889946	0.044091237
10551011	Dmrtc2: doublesex and mab-3 related transcription factor like family C	NM_027732	1.595137615	0.044197581
10430536	Ddx17: DEAD (Asp-Glu-Ala-Asp) box polypeptide 17	NM_199080	1.58984101	0.044419502
10564791	Kif7: kinesin family member 7	NM_010626	1.712894072	0.044421451
10367600	Esr1: estrogen receptor 1 (alpha)	NM_007956	1.610145003	0.044455293
10431722	Gxylt1: glucoside xylosyltransferase 1	NM_001033275	1.673685916	0.044463602
10420470	Gm9022: glyceraldehyde-3-phosphate dehydrogenase pseudogene	XR_004746	1.659757983	0.0445021
10379363	Atad5: ATPase family, AAA domain containing 5	NM_001029856	1.536378471	0.04450846
10420390	Xpo4: exportin 4	NM_020506	2.194690595	0.044523408
10472047	Tas2r134: taste receptor, type 2, member 134	NM_199158	2.013531388	0.044597663
10525374	Pptc7: PTC7 protein phosphatase homolog (S. cerevisiae)	NM_177242	1.849680576	0.044651395
10458999	Fbn2: fibrillin 2	NM_010181	1.621950262	0.044919429
10594447	Map2k1: mitogen-activated protein kinase kinase 1	NM_008927	1.741128811	0.044962971
10415279	Fitm1: fat storage-inducing transmembrane protein 1	NM_026808	1.625074272	0.045021262

10373873	Sf3a1: splicing factor 3a, subunit 1	NM_026175	1.923376578	0.045098188
10367830	Grm1: glutamate receptor, metabotropic 1	NM_001114333	1.672459893	0.045175538
10476691	Polr3f: polymerase (RNA) III (DNA directed) polypeptide F	NM_029763	1.843475182	0.045355657
10577973	Adrb3: adrenergic receptor, beta 3	NM_013462	1.519845995	0.045412592
10390574	Fbxl20: F-box and leucine-rich repeat protein 20	NM_028149	1.622072956	0.04560764
10491060	Spin3: spindlin family, member 3	XR_031023	1.982563544	0.0456614
10594183	Senp8: SUMO/sentrin specific peptidase 8	NM_001172068	1.911256534	0.045721246
10471424	Fam102a: family with sequence similarity 102, member A	NM_153560	1.613905443	0.045793898
10558614	Cd163l1: CD163 molecule-like 1	NM_172909	1.556466232	0.045969219
10488441	Zfp120: zinc finger protein 120	NM_181266	2.212694215	0.046004654
10520483	Ept1: ethanolaminephosphotransferase 1 (CDP-ethanolamine-specific)	NM_027652	1.608054463	0.046010366
10357164	Epb4.1l5: erythrocyte protein band 4.1-like 5	NM_145506	1.608054463	0.046010366
10607395	Mageh1: melanoma antigen, family H, 1	NM_023788	1.739771863	0.046014907
10470283	Egfl7: EGF-like domain 7	NM_198724	1.604708068	0.046062152
10516046	Gm12877: predicted gene 12877	XM_987282	1.642229019	0.046125855
10562599	Gm16387: predicted gene 16387	ENSMUST00000098	1.643589192	0.046318938
10573998	Ogfod1: 2-oxoglutarate and iron-dependent oxygenase domain contai	NM_177767	1.789197176	0.046363664
10568536	Cpxm2: carboxypeptidase X 2 (M14 family)	NM_018867	1.575167153	0.046477616
10454310	Galnt1: UDP-N-acetyl-alpha-D-galactosamine:polypeptide N-acetylgala	NM_013814	1.630993813	0.046496116
10472923	Ak3l1: adenylate kinase 3-like 1	NM_001177602	1.523265782	0.046559851
10574976	Pla2g15: phospholipase A2, group XV	NM_133792	1.832353334	0.046620106
10530892	Cenpc1: centromere protein C1	NM_007683	1.563031516	0.046635193
10466314	Gm6545: predicted gene 6545	XM_889589	1.654064138	0.046640878
10533285	Ptpn11: protein tyrosine phosphatase, non-receptor type 11	NM_011202	1.8567211	0.046644985
10506269	Ak3l1: adenylate kinase 3-like 1	NM_001177602	1.523029696	0.046649142
10479087	Stx16: syntaxin 16	NM_172675	2.001889282	0.046660662
10420488	D14Ertd668e: DNA segment, Chr 14, ERATO Doi 668, expressed	NM_001164323	1.586943502	0.046698345
10499187	Ube2l3: ubiquitin-conjugating enzyme E2L 3	NM_009456	2.149932743	0.046731031
10529873	Rab2a: RAB2A, member RAS oncogene family	NM_021518	1.867241926	0.046799542
10540540	0610010K06Rik: RIKEN cDNA 0610010K06 gene	NM_027861	1.501462454	0.046801397
10568361	Yipf5: Yip1 domain family, member 5	NM_023311	2.198431628	0.046813082
10418053	Kcnma1: potassium large conductance calcium-activated channel, sub	NM_010610	1.708248921	0.046878091
10468980	Fam107b: family with sequence similarity 107, member B	BC021353	1.613921583	0.046879141
10364413	Olfr1355: olfactory receptor 1355	NM_207571	1.797384643	0.046879759
10354003	Mgat4a: mannoside acetylglucosaminyltransferase 4, isoenzyme A	NM_173870	1.748334848	0.046948425
10530641	Usp46: ubiquitin specific peptidase 46	NM_177561	2.167421869	0.047278288
10427814	Golph3: golgi phosphoprotein 3	NM_025673	1.664915526	0.047278866
10470200	Lcn11: lipocalin 11	NM_001100455	1.719286955	0.047368439
10605674	Pola1: polymerase (DNA directed), alpha 1	NM_008892	1.840040471	0.047371297
10522075	Klhl5: kelch-like 5 (Drosophila)	NM_175174	1.715860528	0.047532492
10567626	Gga2: golgi associated, gamma adaptin ear containing, ARF binding pr	NM_028758	1.636506736	0.047551256
10473008	Hnrnpa3: heterogeneous nuclear ribonucleoprotein A3	NM_146130	1.693171997	0.047579078
10565156	Homer2: homer homolog 2 (Drosophila)	NM_011983	1.695566379	0.047596748
10348817	Sept2: septin 2	NM_001159719	1.915477149	0.047629923
10597427	Trim71: tripartite motif-containing 71	NM_001042503	2.121157028	0.047649016
10522530	Kit: kit oncogene	NM_001122733	1.713163579	0.047689665
10425046	Apol7e: apolipoprotein L 7e	XR_004836	1.531742458	0.047699242
10456329	5330437I02Rik: RIKEN cDNA 5330437I02 gene	NM_177028	1.513251128	0.047769469
10499138	Dclk2: doublecortin-like kinase 2	NM_027539	1.58911063	0.04777149
10515090	Cdkn2c: cyclin-dependent kinase inhibitor 2C (p18, inhibits CDK4)	NM_007671	1.561943789	0.047796518
10496359	Emcn: endomucin	NM_001163522	1.606660275	0.047919873
10463121	Zfp518: zinc finger protein 518	NM_028319	2.237606361	0.047946661
10352735	Ints7: integrator complex subunit 7	NM_178632	1.706211106	0.047978402
10563745	Mrgprb5: MAS-related GPR, member B5	NM_207538	1.768995602	0.048045006
10565089	Cpeb1: cytoplasmic polyadenylation element binding protein 1	NM_007755	1.574531164	0.048175388
10570556	Mcph1: microcephaly, primary autosomal recessive 1	NM_173189	1.747120165	0.048304912
10518902	Hes3: hairy and enhancer of split 3 (Drosophila)	NM_008237	1.681741282	0.048399068
10579976	Elmod2: ELMO domain containing 2	NM_178736	1.606015811	0.04841545
10345869	Tmem182: transmembrane protein 182	NM_001081198	2.05563461	0.048433702
10374485	Peli1: pellino 1	NM_023324	1.816885473	0.048608672
10484758	Olfr1198: olfactory receptor 1198	NM_207567	1.792009868	0.048677064
10458663	Dpysl3: dihydropyrimidinase-like 3	NM_009468	1.598641622	0.048824705
10394593	Fam49a: family with sequence similarity 49, member A	NM_029758	1.619137099	0.048951437
10349016	2310035C23Rik: RIKEN cDNA 2310035C23 gene	NM_173187	1.504770525	0.048970844
10577226	2610019F03Rik: RIKEN cDNA 2610019F03 gene	NM_173744	1.507346197	0.048980231

10448062	Gm7177: predicted gene 7177	XR_032603	1.655076346	0.049144581
10548653	Prpmp5: proline-rich protein MP5	NM_001024705	1.855358457	0.049246104
10479063	Rab22a: RAB22A, member RAS oncogene family	NM_024436	1.859689517	0.049280314
10568521	2310057M21Rik: RIKEN cDNA 2310057M21 gene	BC044749	1.50624128	0.049308335
10579736	9130011J15Rik: RIKEN cDNA 9130011J15 gene	BC055692	1.514820499	0.049555213
10518847	Phf13: PHD finger protein 13	NM_172705	1.820049273	0.049572097
10437160	Ets2: E26 avian leukemia oncogene 2, 3' domain	NM_011809	1.612752286	0.04965514
10477924	Tgif2: TGFB-induced factor homeobox 2	NM_173396	2.030303224	0.049676108
10407809	Gpr137b: G protein-coupled receptor 137B	ENSMUST00000021	1.66730116	0.049708255
10344741	Hnrnpa3: heterogeneous nuclear ribonucleoprotein A3	NM_053263	1.694063539	0.049752522
10429524	D730001G18Rik: RIKEN cDNA D730001G18 gene	NR_027836	1.588511954	0.049768991
10565935	Arhgef17: Rho guanine nucleotide exchange factor (GEF) 17	NM_001081116	1.532856026	0.049963464
