# Supplementary material for: The RNA-binding protein DAZL functions as repressor and activator of mRNA translation during oocyte maturation
Source: Nat Commun. 2020 Mar 13;11:1399. doi: 10.1038/s41467-020-15209-9 (PMC7070028; doi:10.1038/s41467-020-15209-9)
Supplement: Supplementary file 5 — Reporting Summary [file 41467_2020_15209_MOESM5_ESM.pdf]

nature research

Corresponding author(s): Marco Conti, NCOMMS-19-10769

Last updated by author(s): Feb. 1st, 2020

Reporting Summary

Nature Research wishes to improve the reproducibility of the work that we publish. This form provides structure for consistency and transparency in reporting. For further information on Nature Research policies, see Authors & Referees and the Editorial Policy Checklist .

Statistics

For all statistical analyses, confirm that the following items are present in the figure legend, table legend, main text, or Methods section.

n/a	Confirmed	
☐	☒	The exact sample size (*n*) for each experimental group/condition, given as a discrete number and unit of measurement
☐	☒	A statement on whether measurements were taken from distinct samples or whether the same sample was measured repeatedly
☐	☒	The statistical test(s) used AND whether they are one- or two-sided<br>*Only common tests should be described solely by name; describe more complex techniques in the Methods section.*
☒	☐	A description of all covariates tested
☐	☒	A description of any assumptions or corrections, such as tests of normality and adjustment for multiple comparisons
☐	☒	A full description of the statistical parameters including central tendency (e.g. means) or other basic estimates (e.g. regression coefficient) AND variation (e.g. standard deviation) or associated estimates of uncertainty (e.g. confidence intervals)
☐	☒	For null hypothesis testing, the test statistic (e.g. *F*, *t*, *r*) with confidence intervals, effect sizes, degrees of freedom and *P* value noted<br>*Give P values as exact values whenever suitable.*
☒	☐	For Bayesian analysis, information on the choice of priors and Markov chain Monte Carlo settings
☒	☐	For hierarchical and complex designs, identification of the appropriate level for tests and full reporting of outcomes
☒	☐	Estimates of effect sizes (e.g. Cohen's *d*, Pearson's *r*), indicating how they were calculated

Our web collection on statistics for biologists contains articles on many of the points above.

Software and code

Policy information about availability of computer code

Data collection	Metamorph 7.8.13.0 software, ImageJ bundled with 64-bit Java 1.8.0_112 software, GraphPad Prism 8.0.2 software<br>RNASeq cDNA library preparation using the Ovation RNA-Seq System V2 (NuGen, 0344). Samples were sequenced using the HiSeq4000 platform.
Data analysis	RNASeq data: QC and Trim(Trimmomatic-0.33)-Align (Galaxy/RNA star, 2.4.2a)-Count aligned reads (Galaxy/htseq-count, Galaxy Version 0.9.1galaxy1)-Determine differentially expressed genes (Galaxy/DESeq2 Version 1.18)

For manuscripts utilizing custom algorithms or software that are central to the research but not yet described in published literature, software must be made available to editors/reviewers. We strongly encourage code deposition in a community repository (e.g. GitHub). See the Nature Research guidelines for submitting code & software for further information.

Data

Policy information about availability of data

All manuscripts must include a data availability statement. This statement should provide the following information, where applicable:

- Accession codes, unique identifiers, or web links for publicly available datasets
- A list of figures that have associated raw data
- A description of any restrictions on data availability

Some of data generated or analysed during this study are included in this published article (and its supplementary information files).
Raw data have been deposited in teh public SRA repository with accession number PRJNA603993

Field-specific reporting

Please select the one below that is the best fit for your research. If you are not sure, read the appropriate sections before making your selection.

- [x] Life sciences
- [ ] Behavioural & social sciences
- [ ] Ecological, evolutionary & environmental sciences

For a reference copy of the document with all sections, see nature.com/documents/nr-reporting-summary-flat.pdf

Life sciences study design

All studies must disclose on these points even when the disclosure is negative.

Sample size	No sample size calculation was performed. The major limitation for the experimental model used is the availability of samples. The RiboTag IP/RMAseq required microinjection of more than 3200 oocytes from wild type mice or Dazl+/- mice. This set an upper limit for the number and replicate (N=2) possible. However, the control group was compared to two additional RiboTag IP/RNAseq dataset and the data were qualitative similar.<br>RiboTag IP/qPCR was performed in three independent sets of samples (200 oocyte/each) and the data were consistent with the RiboTag IP/RNAseq data.<br>Reporter assays in microinjected oocytes was done on three independent oocyte preparations with oocyte samples size varying between 33 and 100 oocytes for wild type and Dazl+/- mice.<br>RIP-Chip was done on triplicate biological samples.<br>Because of the limitation of available mice and sample, we use all of our obtained samples for RiboTag IP/qPCR. In Fig.1 most were done three times The control CccnB1 2 times but the results were consistent with other experiments In Fig 3 some samples are done 3 times but others are done two times.
Data exclusions	In most experiments data were not excluded. In figure 2, one of three Btg4 data in RiboTag IP/qPCR is far away out of the other two, so we excluded that one. In some YFP reporter measurement, sometimes the microscope runs very long time so it would stopped to take picture at some point, which made the data is 0. But overall, the time recording during oocyte maturation is consistent for the idea of Dazl's dual functions.
Replication	Most of the experiments have been repeated at least 3 times. In some cases as in Fig.3 , given the number of mice required to do full 3 replicates, we have repeated the experiment only twice. However, the data are consistent with observations from independent experiments.
Randomization	All wildtype or Dazl +/- oocytes from multiple mice were randomly allocated to different experimental groups. Microinjection was also allocated in random groups for the following experiments.
Blinding	No blinding of data was performed. Because these molecular and cellular biology experiments are difficult to blind as the same individual sets up the experiments and does the measurements and analysis

Reporting for specific materials, systems and methods

We require information from authors about some types of materials, experimental systems and methods used in many studies. Here, indicate whether each material, system or method listed is relevant to your study. If you are not sure if a list item applies to your research, read the appropriate section before selecting a response.

Materials & experimental systems

n/a	Involved in the study	
[ ]	[x]	Antibodies
[ ]	[x]	Eukaryotic cell lines
[x]	[ ]	Palaeontology
[ ]	[x]	Animals and other organisms
[x]	[ ]	Human research participants
[x]	[ ]	Clinical data

Methods

n/a	Involved in the study	
[x]	[ ]	ChIP-seq
[x]	[ ]	Flow cytometry
[x]	[ ]	MRI-based neuroimaging

Antibodies

Antibodies used	RabMAb Anti-DAZL antibody (EPR21028, ab215718, Abcam); RabMAb Anti-DAZL antibody(EPR21028, ab228135, Abcam), Monoclonal Anti-α-Tubulin antibody produced in mouse (T6074, clone AA13, purified from hybridoma cell culture,Sigma-Aldrich); anti-HA.11 Epitope Tag antibody (clone:16B12, Cat: 901513,Lot:B220849, BioLegend), Normal mouse IgG antibody(Cat: 12-371, Lot:2757162, Sigma-Aldrich). HRP-conjugated secondary antibodies (anti rabbit IgG: LNA934V/AH, lot9761196, anti mouse IgG: LNA934V/AH, lot 9739640 Sigma aldrich), rabbit IgG (ab37415; Abcam). actin (ab14128, Abcam), V5 antibody (R960-25, Invitrogen)
Validation	1. https://www.abcam.com/dazl-antibody-epr21028-ab215718-references.html#top-684 (Our data supplied to the company)<br>2. https://www.abcam.com/dazl-antibody-epr21028-bsa-and-azide-free-ab228135.html<br>3. https://www.sigmaaldrich.com/catalog/product/sigma/t6074?lang=en&region=US<br>TUBA4A gene analysis in sporadic amyotrophic lateral sclerosis: identification of novel mutations. Pensato V, et.al. Journal of Neurology 262(5), 1376-1378, (2015)<br>Specific inhibition of NF-Y subunits triggers different cell proliferation defects. Benatti P, et.al. Nucleic Acids Research 39(13), 5356-5368, (2011)<br>ncreased expression of αTubulin is associated with poor prognosis in patients with pancreatic cancer after surgical resection. Chao L, et al. Oncotarget 7(37), 60657-60664, (2016)<br>4. https://www.biolegend.com/en-us/products/anti-ha-11-epitope-tag-antibody-11071<br>Hogarth C, et al. 2015. Biol Reprod. . PubMed<br>Görtz D, et al. 2015. Sci Rep. 5: 14685. PubMed<br>Wilson C, et al. 2015. PLoS One. 10: 0139579. PubMed

Smith B, et al. 2012. Genes Cancer. 3:550-563. PubMed
Liu Z, et al. 2016. Nature. 530:98-102. PubMed
Thoms M, et al. 2016. Nucleic Acids Res. 44: 926 - 939. PubMed
Kim Y, et al. 2016. Nat Commun. 7:10347. PubMed
Rodríguez-Escudero M, et al. 2016. PLoS One. 11: 0148032. PubMed
Lehmann W, et al. 2016. Nat Commun. 7:10498. PubMed
Testoni E, et al. 2016. EMBO Mol Med. 8: 105 - 116. PubMed
Padilla S, et al. 2016. Nat Neurosci. 10.1038/nn.4274. PubMed
Martins J, et al. 2016. J Cell Sci. 129: 1271 - 1282. PubMed
5. http://www.emdmillipore.com/US/en/product/Normal-Mouse-IgG,MM_NF-12-371?ReferrerURL=https%3A%2F%2Fwww.google.com%2F&bd=1
Histone monoubiquitination by Clock-Bmal1 complex marks Per1 and Per2 genes for circadian feedback.
Tamayo, AG; Duong, HA; Robles, MS; Mann, M; Weitz, CJ Nature structural & molecular biology 22 759-66 2015
Ezh2 mediated H3K27me3 activity facilitates somatic transition during human pluripotent reprogramming.
Rao, RA; Dhele, N; Cheemadan, S; Ketkar, A; Jayandharan, GR; Palakodeti, D; Rampalli, S Scientific reports 5 8229 2015
6.https://www.abcam.com/rabbit-igg-polyclonal-isotype-control-ab37415.html. Cioni JM et al. Late Endosomes Act as mRNA Translation Platforms and Sustain Mitochondria in Axons. Cell 176:56-72.e15 (2019).
7. https://www.abcam.com/mouse-igg-isotype-control-ab37355.html, Terré B et al. Defects in efferent duct multiciliogenesis underlie male infertility in GEMC1-, MCIDAS- or CCNO-deficient mice. Development 146:N/A (2019).
8. https://www.sigmaaldrich.com/catalog/product/sigma/gena9341ml?lang=en&region=US&cm_sp=Insite-_-recent_fixed-_-recent5-2
E proteins sharpen neurogenesis by modulating proneural bHLH transcription factors' activity in an E-box-dependent manner.
9. https://www.abcam.com/actin-antibody-c4-ab14128.html
Chen YL et al. Adiponectin receptor PAQR-2 signaling senses low temperature to promote C. elegans longevity by regulating autophagy. Nat Commun 10:2602 (2019).
10. https://www.thermofisher.com/antibody/product/V5-Tag-Antibody-Monoclonal/R960-25

Eukaryotic cell lines

Policy information about cell lines

Cell line source(s)	Mouse Embryonic Stem Cells E14 ES cells was provided by UCSF stem cell research labortary .
Authentication	The ES cell line used was not authenticated. E14 is the most widely used mouse ES cell line
Mycoplasma contamination	The cell lines were not tested for mycoplasma contamination.
Commonly misidentified lines (See ICLAC register)	No commoly misdentified cell lines were used in the study.

Animals and other organisms

Policy information about studies involving animals; ARRIVE guidelines recommended for reporting animal research

Laboratory animals	Pure C57BL/6 female mice (21-24 days old) carrying the DAZL TM1Hgu allele (ΔDAZL). Rpl22tm1.1Psam/J (RiboTag) mice, with a targeted mutation that provides conditional expression of the ribosomal protein L22 tagged with three copies of the HA epitope. Rpl22tm1.1Psam/J homozygous males were crossed with C57BL/6-TgN (Zp3-cre) 82Knw (Jackson Laboratories) females to produce C57BL/6-Zp3cre-Rpl22tm1. 1Psam (Zp3cre-RiboTag) mice. For breeding Zp3RiboTagDazl+/+ or Zp3RiboTagDazl+/-, C57BL/6-Zp3cre- RiboTag wild type or homozygous males were crossed with C57BL/6- RiboTag wild type or heterozygous ΔDAZL females to obtain C57BL/6-ΔDAZL-ZP3cre-RiboTag mice. All mice used in this study is C57BL/6 female mice at the age of 21-24 days.
Wild animals	No wild animals were used in the study.
Field-collected samples	No field collected samples were used in the study.
Ethics oversight	Institutional Animal Care and Use Committee of the University of California at San Francisco (protocol AN101432)

Note that full information on the approval of the study protocol must also be provided in the manuscript.
